# Supplementary material for: Mountain colonisation, miniaturisation and ecological evolution in a radiation of direct-developing New Guinea Frogs (Choerophryne, Microhylidae)
Source: PeerJ. 2017 Mar 30;5:e3077. doi: 10.7717/peerj.3077 (PMC5376113; doi:10.7717/peerj.3077)
Supplement: Appendix S1 — Supplementary tables and figures. Specimen numbers, locality information and GenBank accession numbers for Choerophryne specimens included in analyses (Table S1); GenBank accession details for outgroup samples (Table S2); genetic distance data for species and candidate lineages (Table S3); and summary data on body, elevational distribution and calling height for Choerophryne (Table S4). Bayesian tree for all samples (Figure S1); Trait evolution in the major lineages of Choerophryne estimated using BEAST (Figure S2); and summary of museum records for Choerophryne grouped by phenotype (Figure S3). [file peerj-05-3077-s001.docx]

**SUPPORTING INFORMATION**

**Mountain colonisation, miniaturisation and ecological evolution in New Guinea Frogs (*Choerophryne*, Microhylidae)**

Paul M. Oliver, Amy Iannella, Stephen J. Richards and Michael S.Y. Lee

**Appendix S1** Summary data for all ingroup and outgroup specimens included in phylogenetic and morphological analyses, and overall ecophenotypic data for major lineages.

**Table S1.**

Summary information for all *Choerophryne* specimens examined in this study.

| **Name** | ***Species*** | **Clade** | **Locality** | **Country** | **Province** | **Specimen** | **12S** | **16S** | **Latitude** | **Longitude** | **Altitude (m a.s.l)** |
| --- | --- | --- | --- | --- | --- | --- | --- | --- | --- | --- | --- |
| SJR9565 | *alpestris* | a | Kaijende Highlands | Papua New Guinea | Enga Province | SJR9565 | KY626072 | KY438136 | -5.54 | 143.05 | 3200 |
| SJR9553 | *alpestris* | a | Kaijende Highlands | Papua New Guinea | Enga Province | SJR9553 | KY626073 | KY438137 | -5.54 | 143.05 | 3200 |
| SJR9545 | *alpestris* | a | Kaijende Highlands | Papua New Guinea | Enga Province | SJR9545 | KY626074 | KY438138 | -5.54 | 143.05 | 3200 |
| SJR9070 | *brevicrus* | a | Spur off Muller Range | Papua New Guinea | Hela Province | SJR9070 |  | KY438119 | -5.95 | 142.75 | 2275 |
| SJR9069 | *brevicrus* | a | Spur off Muller Range | Papua New Guinea | Hela Province | SJR9069 | KY626107 | KY438172 | -5.95 | 142.75 | 2275 |
| UPNG10060 | *brevicrus* | a | Lake Tawa - Porgera | Papua New Guinea | Enga Province | UPNG10060 | KY626055 | KY438116 | -5.60 | 142.84 | 2300 |
| SAMAR66098 | *brevicrus* | a | Lake Tawa - Porgera | Papua New Guinea | Enga Province | SAMAR66098 |  | KY438115 | -5.60 | 142.84 | 2300 |
| ZMB76744 | *brevicrus* | a | Lake Tawa - Porgera | Papua New Guinea | Enga Province | ZMB76744 |  |  | -5.60 | 142.84 | 2300 |
| SJR9066 | *darlingtoni* | a | Spur off Muller Range | Papua New Guinea | Hela Province | SJR9066 | KY626108 | KY438173 | -5.95 | 142.75 | 2275 |
| SJR12008 | *darlingtoni* | a | Muller Range, Western Province | Papua New Guinea | Western Province | SJR12008 |  | KY438146 | -5.66 | 142.30 | 1630 |
| SJR12011 | *darlingtoni* | a | Muller Range, Western Province | Papua New Guinea | Western Province | SJR12011 |  | KY438125 | -5.66 | 142.30 | 1630 |
| SJR12014 | *darlingtoni* | a | Muller Range | Papua New Guinea | Western Province | SJR12014 | KY626059 |  | -5.66 | 142.30 | 1630 |
| SJR10536 | *fafniri* | a | Central Ranges | Papua New Guinea | Hela Province | SJR10536 | KY626097 | KY438163 | -6.10 | 143.10 | 2060 |
| SJR10545 | *fafniri* | a | Central Ranges | Papua New Guinea | Hela Province | SJR10545 | KY626068 |  | -6.10 | 143.10 | 2060 |
| SJR10548 | *fafniri* | a | Central Ranges | Papua New Guinea | Hela Province | SJR10548 |  | KY438131 | -6.10 | 143.10 | 2060 |
| SJR9622 | *fafniri* | a | Lake Tawa - Porgera | Papua New Guinea | Enga Province | SJR9622 | KY626122 | KY438182 | -5.60 | 142.84 | 2300 |
| SJR9638 | *fafniri* | a | Lake Tawa - Porgera | Papua New Guinea | Enga Province | SJR9638 | KY626056 | KY438117 | -5.60 | 142.84 | 2300 |
| ZMB70313 | *laurini* | a | Wandamen Peninsula | Indonesia | West Papua Province | ZMB70313 | EU100107 | EU100223 | -2.81 | 134.58 | 800-950 |
| ZMB61913 | *laurini* | a | Wandamen Peninsula | Indonesia | West Papua Province | ZMB61913 | EU100104 | EU100220 | -2.81 | 134.58 | 800-950 |
| ZMB70312 | *laurini* | a | Wandamen Peninsula | Indonesia | West Papua Province | ZMB70312 | EU100106 | EU100222 | -2.81 | 134.58 | 800-950 |
| ZMB70311 | *laurini* | a | Wandamen Peninsula | Indonesia | West Papua Province | ZMB70311 | EU100105 | EU100221 | -2.81 | 134.58 | 800-950 |
| ZMB76958 | *pandanicola* | a | Kaijende Highlands | Papua New Guinea | Enga Province | ZMB76958 | KY626121 | KY438181 | -5.50 | 143.08 | 2900 |
| SAMAR66101 | *pandanicola* | a | Kaijende Highlands | Papua New Guinea | Enga Province | SAMAR66101 |  | KY438180 | -5.50 | 143.08 | 2900 |
| SJR8644 | *siegfriedi* | a | Mt Elimbari | Papua New Guinea | Chimbu Province | SJR8644 |  | KY438166 | -6.19 | 145.16 | 2500 |
| SJR8649 | *siegfriedi* | a | Mt Elimbari | Papua New Guinea | Chimbu Province | SJR8649 |  | KY438129 | -6.19 | 145.16 | 2500 |
| SJR8658 | *siegfriedi* | a | Mt Elimbari | Papua New Guinea | Chimbu Province | SJR8658 |  | KY438128 | -6.19 | 145.16 | 2500 |
| SJR10537 | sp A1 | a | Central Ranges | Papua New Guinea | Hela Province | SJR10537 | KY626096 | KY438162 | -6.10 | 143.10 | 2060 |
| SJR10549 | sp A1 | a | Central Ranges | Papua New Guinea | Hela Province | SJR10549 | KY626095 | KY438161 | -6.10 | 143.10 | 2060 |
| SJR9091 | sp A1 | a | Spur off Muller Range | Papua New Guinea | Hela Province | SJR9091 |  | KY438118 | -5.95 | 142.75 | 2275 |
| SJR12109 | sp A2 | a | Muller Range | Papua New Guinea | Southern Highlands Province | SJR12109 | KY626077 | KY438142 | -5.49 | 142.30 | 2875 |
| SJR8500 | sp A3 | a | Moran | Papua New Guinea | Southern Highlands Province | SJR8500 | KY626093 | KY438159 | -6.24 | 143.13 | 1700 |
| SJR9834 | sp A4 | a | Foya Mountains | Indonesia | Papua Province | SJR9834 | KY626132 | KY438190 | -2.57 | 138.72 | 1600 |
| SJR9835 | sp A4 | a | Foya Mountains | Indonesia | Papua Province | SJR9835 | KY626131 | KY438189 | -2.57 | 138.72 | 1600 |
| SJR9924 | sp A5 | a | Foya Mountains | Indonesia | Papua Province | SJR9924 | KY626130 | KY438188 | -2.59 | 138.72 | 1220 |
| SJR9926 | sp A5 | a | Foya Mountains | Indonesia | Papua Province | SJR9926 | KY626129 | KY438187 | -2.59 | 138.72 | 1220 |
| SJR2325 | sp A6 | a | Iagifu Ridge Summit | Papua New Guinea | Southern Highlands Province | SJR2325 | KY626110 |  | -6.43 | 143.21 | 1300 |
| SJR12004 | sp A6 | a | Muller Range, Western Province | Papua New Guinea | Western Province | SJR12004 | KY626079 | KY438144 | -5.66 | 142.30 | 1630 |
| SJR12013 | sp A6 | a | Muller Range, Western Province | Papua New Guinea | Western Province | SJR12013 |  | KY438124 | -5.66 | 142.30 | 1630 |
| SJR12049 | sp A6 | a | Muller Range, Western Province | Papua New Guinea | Western Province | SJR12049 |  | KY438123 | -5.66 | 142.30 | 1630 |
| SJR12003 | sp A6 | a | Muller Range, Western Province | Papua New Guinea | Western Province | SJR12003 |  | KY438145 | -5.66 | 142.30 | 1630 |
| SJR8566 | sp A6 | a | Moran | Papua New Guinea | Southern Highlands Province | SJR8566 | KY626066 |  | -6.24 | 143.13 | 1700 |
| SJR8505 | sp A6 | a | Moran | Papua New Guinea | Southern Highlands Province | SJR8505 | KY626092 | KY438157 | -6.24 | 143.13 | 1700 |
| SJR10484 | sp A7 | a | Upper Strickland | Papua New Guinea | Western Province | SJR10484 | KY626069 | KY438132 | -5.81 | 142.31 | 213 |
| SJR10483 | sp A7 | a | Upper Strickland | Papua New Guinea | Western Province | SJR10483 | KY626098 | KY438164 | -5.81 | 142.31 | 213 |
| SJR10474 | sp A7 | a | Upper Strickland | Papua New Guinea | Western Province | SJR10474 | KY626094 | KY438160 | -5.81 | 142.31 | 213 |
| SJR10871 | sp A7 | a | Muller Range | Papua New Guinea | Western Province | SJR10871 | KY626082 |  | -5.73 | 142.26 | 515 |
| SJR10872 | sp A7 | a | Muller Range | Papua New Guinea | Western Province | SJR10872 | KY626081 | KY438148 | -5.73 | 142.26 | 515 |
| SJR10873 | sp A7 | a | Muller Range | Papua New Guinea | Western Province | SJR10873 | KY626061 | KY438127 | -5.73 | 142.26 | 515 |
| SJR10874 | sp A7 | a | Muller Range | Papua New Guinea | Western Province | SJR10874 | KY626060 | KY438126 | -5.73 | 142.26 | 515 |
| SJR8620 | sp A7 | a | Moro Seepage ~ 1 km from boom gate | Papua New Guinea | Southern Highlands Province | SJR8620 | KY626102 | KY438168 | -6.37 | 143.22 | 928 |
| SJR8619 | sp A7 | a | Moro Seepage ~ 1 km from boom gate | Papua New Guinea | Southern Highlands Province | SJR8619 | KY626065 |  | -6.37 | 143.22 | 928 |
| SJR10413 | sp A7 | a | Upper Strickland | Papua New Guinea | Western Province | SJR10413 |  | KY438133 | -5.90 | 142.44 | 953 |
| SJR10412 | sp A7 | a | Upper Strickland | Papua New Guinea | Western Province | SJR10412 | KY626070 | KY438134 | -5.90 | 142.44 | 953 |
| SJR2280 | sp A7 | a | Iagifu Ridge Summit | Papua New Guinea | Southern Highlands Province | SJR2280 | KY626112 |  | -6.43 | 143.21 | 1300 |
| SJR2279 | sp A7 | a | Iagifu Ridge Summit | Papua New Guinea | Southern Highlands Province | SJR2279 | KY626058 | KY438121 | -6.43 | 143.21 | 1300 |
| SJR2319 | sp A8 | a | Iagifu Ridge summit | Papua New Guinea | Southern Highlands Province | SJR2319 | KY626111 |  | -6.43 | 143.21 | 1300 |
| SJR10221 | sp A8 | a | Karius Range | Papua New Guinea | Hela Province | SJR10221 | KY626071 | KY438135 | -5.99 | 142.67 | 1368 |
| SJR8504 | sp A8 | a | Moran | Papua New Guinea | Southern Highlands Province | SJR8504 |  | KY438158 | -6.24 | 143.13 | 1700 |
| SJR8556 | sp A8 | a | Moran | Papua New Guinea | Southern Highlands Province | SJR8556 |  | KY438130 | -6.24 | 143.13 | 1700 |
| ZMB70342 | *amomani* | b | Amoman Mountains, Pulau Japen | Indonesia | Papua Province | ZMB70342 | EU100136 | EU100252 | -1.75 | 136.32 | 1050-1100 |
| ZMB70348 | *arndatorum* | b | Japen Island | Indonesia | Papua Province | ZMB70348 | EU100140 | EU100256 | -1.78 | 136.33 | 550-650 |
| ZMB70352 | *arndatorum* | b | Japen Island | Indonesia | Papua Province | ZMB70352 | EU100141 | EU100257 | -1.78 | 136.33 | 550-650 |
| ZMB70346 | *arndatorum* | b | Japen Island | Indonesia | Papua Province | ZMB70346 | EU100139 | EU100255 | -1.78 | 136.33 | 550-650 |
| SJR12022 | *burtoni* | b | Muller Range, Western Province | Papua New Guinea | Western province | SJR12022 | KY626078 | KY438143 | -5.66 | 142.30 | 1630 |
| SJR8511 | *burtoni* | b | Moran | Papua New Guinea | Southern Highlands Province | SJR8511 | KY626091 | KY438156 | -6.24 | 143.13 | 1700 |
| SJR3295 | *burtoni* | b | Moran | Papua New Guinea | Southern Highlands Province | SJR3295 | KY626123 |  | -6.24 | 143.13 | 1700 |
| SAMAR67920 | *epirrhina* | b | North Slope of Central Cordillera | Papua New Guinea | Sandaun Province | SAMAR67920 | KY626063 | KM362424 | -4.65 | 141.72 | 850 |
| PNGNM24045 | *epirrhina* | b | North Slope of Central Cordillera | Papua New Guinea | Sandaun Province | PNGNM24045 | KY626064 | KM362423 | -4.65 | 141.72 | 850 |
| SAMAR67519 | *gracilirostris* | b | Upper Strickland River | Papua New Guinea | Western Province | SAMAR67519 | KY626119 | KF561136 | -5.81 | 142.31 | 213 |
| SAMAR67520 | *gracilirostris* | b | Upper Strickland River | Papua New Guinea | Western Province | SAMAR67520 | KY626118 | KY438177 | -5.81 | 142.31 | 213 |
| SAMAR64986 | *gracilirostris* | b | Muller Range | Papua New Guinea | Western Province | SAMAR64986 | KY626080 | KY438147 | -5.73 | 142.26 | 515 |
| SAMAR67514 | *gracilirostris* | b | Karius Range | Papua New Guinea | Hela Province | SAMAR67514 | KY626120 | KY438179 | -5.99 | 142.67 | 1368 |
| SAMAR67517 | *gracilirostris* | b | Karius Range | Papua New Guinea | Hela Province | SAMAR67517 |  | KY438178 | -5.99 | 142.67 | 1368 |
| SAMAR67921 | *grylloides* | b | North Slope of Central Cordillera | Papua New Guinea | Sandaun Province | SAMAR67921 | KY626062 | KM362425 | -4.65 | 141.72 | 900 |
| SAMAR65929 | *longirostris* | b | Mt Menawa | Papua New Guinea | Sandaun Province | SAMAR65929 | KY626128 | KY438186 | -3.31 | 141.70 | 780 |
| SAMAR65930 | *longirostris* | b | Mt Menawa | Papua New Guinea | Sandaun Province | SAMAR65930 | KY626127 | KY438185 | -3.31 | 141.70 | 780 |
| SAMAR65931 | *longirostris* | b | Mt Menawa | Papua New Guinea | Sandaun Province | SAMAR65931 | KY626126 | KY438184 | -3.31 | 141.70 | 780 |
| SAMAR65932 | *longirostris* | b | Mt Menawa | Papua New Guinea | Sandaun Province | SAMAR65932 | KY626125 |  | -3.31 | 141.70 | 780 |
| ZMB0359 | *microps* | b | Wondowoi Mountains | Indonesia | West Papua Province | ZMB70359 | EU100137 | EU100253 | -2.97 | 134.63 | 380-900 |
| ZMB70360 | *microps* | b | Wondowoi Mountains | Indonesia | West Papua Province | ZMB70360 | EU100138 | EU100254 | -2.97 | 134.63 | 380-900 |
| SJR6052 | *nigrescens* | b | Foya Mountains | Indonesia | Papua Province | SJR6052 | KY626133 | KY438191 | -2.37 | 138.21 | 500 |
| ZMB70354 | *nigrescens* | b | Japen Island | Indonesia | Papua Province | ZMB70354 | EU100142 | EU100258 | -1.78 | 136.33 | 650 |
| SAMAR60661 | *proboscidea* | b | Wamangu | Papua New Guinea | East Sepik Province | SAMAR60661 |  |  | -3.79 | 143.65 | 110 |
| SAMAR60663 | *proboscidea* | b | Wamangu | Papua New Guinea | East Sepik Province | SAMAR60663 |  |  | -3.79 | 143.65 | 110 |
| SAMAR60662 | *proboscidea* | b | Wamangu | Papua New Guinea | East Sepik Province | SAMAR60662 |  |  | -3.79 | 143.65 | 110 |
| SJR8985 | *proboscidea* | b | Wamangu | Papua New Guinea | East Sepik Province | SJR8985 | KY626116 |  | -3.79 | 143.65 | 110 |
| SJR8673 | *proboscidea* | b | Wamangu | Papua New Guinea | East Sepik Province | SJR8673 | KY626100 | KY438165 | -3.79 | 143.65 | 110 |
| SJR8995 | *proboscidea* | b | Wamangu | Papua New Guinea | East Sepik Province | SJR8995 | KY626106 |  | -3.79 | 143.65 | 110 |
| SJR8994 | *proboscidea* | b | Wamangu | Papua New Guinea | East Sepik Province | SJR8994 | KY626115 | KY438176 | -3.79 | 143.65 | 110 |
| SAMAR60659 | *rostellifer* | b | Utai | Papua New Guinea | Sandaun Province | SAMAR60659 | KY626067 |  | -3.68 | 141.58 | 100 |
| SJR3766 | *rostellifer* | b | Utai | Papua New Guinea | Sandaun Province | SJR3766 | KY626117 |  | -3.68 | 141.58 | 100 |
| SAMAR60654 | *rostellifer* | b | Utai | Papua New Guinea | Sandaun Province | SAMAR60654 | KY626099 |  | -3.68 | 141.58 | 100 |
| SJR9852 | sp B1 | b | Foya Mountains | Indonesia | Papua Province | SJR9852 | KY626089 | KY438155 | -2.57 | 138.72 | 1600 |
| MZB11978 | sp B1 | b | Foya Mountains | Indonesia | Papua Province | MZB11978 | KY626088 | KY438154 | -2.57 | 138.72 | 1600 |
| SJR9858 | sp B1 | b | Foya Mountains | Indonesia | Papua Province | SJR9858 |  | KY438122 | -2.57 | 138.72 | 1600 |
| SJR13272 | sp B2 | b | Central Lowlands | Papua New Guinea | Gulf Province | SJR13272 | KY626054 | KY438114 | -7.03 | 144.93 | 65 |
| SJR13273 | sp B2 | b | Central Lowlands | Papua New Guinea | Gulf Province | SJR13273 | KY626053 | KY438113 | -7.03 | 144.93 | 65 |
| SJR9089 | sp B3 | b | Spur off Muller Range | Papua New Guinea | Hela Province | SJR9089 | KY626090 |  | -5.95 | 142.75 | 2275 |
| SJR9649 | sp B3 | b | Lake Tawa - Porgera | Papua New Guinea | Enga Province | SJR9649 | KY626076 | KY438141 | -5.60 | 142.84 | 2300 |
| SJR2232 | *exclamitans* | c | Huon Peninsula | Papua New Guinea | Morobe Province | SJR2232 | KY626124 | KY438183 | -6.10 | 146.56 | 1830 |
| SJR2231 | *exclamitans* | c | Huon Peninsula | Papua New Guinea | Morobe Province | SJR2231 |  | KY438140 | -6.10 | 146.56 | 1830 |
| SJR13320 | sp C1 | c | Central Lowlands | Papua New Guinea | Gulf Province | SJR13320 |  |  | -6.89 | 144.89 | 90 |
| SJR3266 | sp C1 | c | Libano | Papua New Guinea | Southern Highlands Province | SJR3266 | KY626057 | KY438120 | -6.40 | 142.98 | 253 |
| SJR3147 | sp C1 | c | Darai | Papua New Guinea | Gulf Province | SJR3147 | KY626109 |  | -7.13 | 143.61 | 440 |
| SJR3148 | sp C1 | c | Darai | Papua New Guinea | Gulf Province | SJR3148 | KY626075 | KY438139 | -7.13 | 143.61 | 440 |
| SJR8622 | sp C1 | c | Moro Seepage ~ 1 km from boom gate | Papua New Guinea | Southern Highlands Province | SJR8622 | KY626101 | KY438167 | -6.37 | 143.22 | 928 |
| SJR9581 | *fafniri* | a | Lake Tawa - Porgera | Papua New Guinea |  | SJR9581 |  |  | -5.60 | 142.84 | 2300 |
| SJR6028 | *arndtorum* | b | Foya Mountains | Indonesia | Papua Province | SJR6028 | KY626105 |  | -2.37 | 138.21 | 500 |
| SAMAR67518 | *gracilirostris* | b | Upper Strickland River | Papua New Guinea | Western Province | SAMAR67518 |  |  | -5.90 | 142.44 | 953 |
| SAMAR67515 | *gracilirostris* | b | Karius Range | Papua New Guinea | Hela Province | SAMAR67515 |  |  | -5.99 | 142.67 | 1368 |
| SAMAR67516 | *gracilirostris* | b | Karius Range | Papua New Guinea | Hela Province | SAMAR67516 |  |  | -5.99 | 142.67 | 1368 |
| SAMAR67928 | *grylloides* | b | North Slope of Central Cordillera | Papua New Guinea | Sandaun Province | SAMAR67928 |  |  | -4.62 | 141.69 | 440 |
| SJR6186 | *nigrescens* | b | Marina Valen Village | Indonesia | Papua Province | SJR6186 |  |  | -2.39 | 138.20 | 500 |
| SAMAR60664 | *proboscidea* | b | Wamangu | Papua New Guinea | East Sepik Province | SAMAR60664 |  |  | -3.79 | 143.65 | 110 |
| SAMAR60665 | *proboscidea* | b | Wamangu | Papua New Guinea | East Sepik Province | SAMAR60665 |  |  | -3.79 | 143.65 | 110 |
| SAMAR60666 | *proboscidea* | b | Wamangu | Papua New Guinea | East Sepik Province | SAMAR60666 |  |  | -3.79 | 143.65 | 110 |
| SAMAR60667 | *proboscidea* | b | Wamangu | Papua New Guinea | East Sepik Province | SAMAR60667 |  |  | -3.79 | 143.65 | 110 |
| SAMAR60668 | *proboscidea* | b | Wamangu | Papua New Guinea | East Sepik Province | SAMAR60668 |  |  | -3.79 | 143.65 | 110 |
| SAMAR60669 | *proboscidea* | b | Wamangu | Papua New Guinea | East Sepik Province | SAMAR60669 |  |  | -3.79 | 143.65 | 110 |
| SJR7302 | *proboscidea* | b | Wanang | Papua New Guinea | Madang Province | SJR7302 | KY626113 | KY438174 | -5.23 | 145.20 | 150 |
| SJR7300 | *proboscidea* | b | Wanang | Papua New Guinea | Madang Province | SJR7300 | KY626104 | KY438171 | -5.23 | 145.20 | 150 |
| SJR7301 | *proboscidea* | b | Wanang | Papua New Guinea | Madang Province | SJR7301 | KY626103 | KY438170 | -5.23 | 145.20 | 150 |
| SJR7272 | *proboscidea* | b | Wanang | Papua New Guinea | Madang Province | SJR7272 | KY626083 | KY438149 | -5.23 | 145.20 | 150 |
| SJR7145 | *proboscidea* | b | Yoro | Papua New Guinea | East Sepik Province | SJR7145 | KY626114 | KY438175 | -4.51 | 145.15 | 200 |
| SJR7184 | *proboscidea* | b | Yoro | Papua New Guinea | East Sepik Province | SJR7184 | KY626087 | KY438153 | -4.51 | 145.15 | 200 |
| SJR7211 | *proboscidea* | b | Yoro | Papua New Guinea | East Sepik Province | SJR7211 | KY626086 | KY438152 | -4.51 | 145.15 | 200 |
| SJR7217 | *proboscidea* | b | Yoro | Papua New Guinea | East Sepik Province | SJR7217 | KY626085 | KY438151 | -4.51 | 145.15 | 200 |
| SJR7224 | *proboscidea* | b | Yoro | Papua New Guinea | East Sepik Province | SJR7224 | KY626084 | KY438150 | -4.51 | 145.15 | 200 |
| SAMAR60653 | *rostellifer* | b | Utai | Papua New Guinea | Sandaun Province | SAMAR60653 |  |  | -3.68 | 141.58 | 100 |
| SAMAR60655 | *rostellifer* | b | Utai | Papua New Guinea | Sandaun Province | SAMAR60655 |  |  | -3.68 | 141.58 | 100 |
| SAMAR60657 | *rostellifer* | b | Utai | Papua New Guinea | Sandaun Province | SAMAR60657 |  |  | -3.68 | 141.58 | 100 |
| SAMAR60658 | *rostellifer* | b | Utai | Papua New Guinea | Sandaun Province | SAMAR60658 |  |  | -3.68 | 141.58 | 100 |
| SAMAR60660 | *rostellifer* | b | Utai | Papua New Guinea | Sandaun Province | SAMAR60660 |  |  | -3.68 | 141.58 | 100 |
| SJR13068 | sp. | b | Sepik Basin | Papua New Guinea | Sandaun Province | SJR13068 |  |  | -4.55 | 141.96 | 70 |
| MZB1200 | sp. | b | Foya Mountains | Indonesia | Papua Province | MZB1200 |  |  | -2.57 | 138.72 | 1600 |
| MZB11980 | sp. | b | Foya Mountains | Indonesia | Papua Province | MZB11980 |  |  | -2.57 | 138.72 | 1600 |
| SJR13069 | sp. | b | Sepik Basin | Papua New Guinea | Sandaun Province | SJR13069 |  |  | -4.55 | 141.96 | 70 |
| SJR3309 | *burtoni* | d | Moran | Papua New Guinea | Southern Highlands Province | SJR3309 |  |  | -6.24 | 143.13 | 1700 |
| SAMAR62475 | *burtoni* | d | Moran | Papua New Guinea | Southern Highlands Province | SAMAR62475 |  |  | -6.24 | 143.13 | 1700 |
| SAMAR62476 | *burtoni* | d | Moran | Papua New Guinea | Southern Highlands Province | SAMAR62476 |  |  | -6.24 | 143.13 | 1700 |
| SJR9650 | *porgera* | d | Lake Tawa - Porgera | Papua New Guinea | Enga Province | SJR9650 |  |  | -5.60 | 142.84 | 2300 |
| SJR14303 | sp. | d | Central Lowlands | Papua New Guinea | Gulf Province | SJR14303 |  |  | -6.94 | 145.10 | 90 |
| SJR14331 | sp. | d | Central Lowlands | Papua New Guinea | Gulf Province | SJR14331 |  |  | -6.98 | 144.99 | 100 |
| SJR14332 | sp. | d | Central Lowlands | Papua New Guinea | Gulf Province | SJR14332 |  |  | -6.98 | 144.99 | 100 |
| SJR14346 | sp. | d | Central Lowlands | Papua New Guinea | Gulf Province | SJR14346 |  |  | -6.98 | 144.99 | 100 |

**Table S2. Summary information for outgroup genetic samples**

| **Genus** | **Species** | **Specimen** | **12S** | **16S** |
| --- | --- | --- | --- | --- |
| *Cophixalus* | *balbus* | ZMB62594 | EU100143 | EU100259 |
| *Cophixalus* | *humicola* | ZMB69704 | EU100147 | EU100263 |
| *Cophixalus* | *tridactylus* | ZMB69696 | EU100149 | EU100265 |
| *Copiula* | *major* | ZMB62074 | EU100152 | EU100268 |
| *Copiula* | *obsti* | ZMB62555 | EU100154 | EU100270 |
| *Copiula* | *pipiens* | ZMB64112 | EU100158 | EU100274 |
| *Genyophryne* | *thomsoni* | BPBM20357 | EU100159 | EU100275 |
| *Liophryne* | *dentata* | BPBM15370 | EU100178 | EU100294 |
| *Liophryne* | *schlaginhaufeni* | BPBM22754 | EU100179 | EU100295 |
| *Oreophryne* | *atrigularis* | ZMB62216 | EU100182 | EU100298 |
| *Oreophryne* | *clamata* | ZMB67353 | EU100189 | EU100305 |
| *Oreophryne* | *sibilans* | RG6936 | EU100194 | EU100310 |
| *Oreophryne* | *unicolor* | ZMB70188 | EU100199 | EU100315 |
| *Oxydactyla* | *crassa* | BPBM17061 | EU100207 | EU100323 |

**Table S3. 16s genetic distances between recognised species and candidate species of *Choerophryne*.**

|  | *exclamitans* | spC1 | *longirostris* | *proboscidea* | spA7 | spA8 | *laurini* | spA5 | spA4 | spA3 | *alpestris* | spA2 | *brevicrus* | spA6 | spA1 | *pandanicola* | *darlingtoni* | *fafniri* | *siegfriedi* | *epirrhina* | *nigrescens* |
| --- | --- | --- | --- | --- | --- | --- | --- | --- | --- | --- | --- | --- | --- | --- | --- | --- | --- | --- | --- | --- | --- |
| *exclamitans* | _ |  |  |  |  |  |  |  |  |  |  |  |  |  |  |  |  |  |  |  |  |
| spC1 | 4.5 | _ |  |  |  |  |  |  |  |  |  |  |  |  |  |  |  |  |  |  |  |
| *longirostris* | 18.3 | 18.9 | _ |  |  |  |  |  |  |  |  |  |  |  |  |  |  |  |  |  |  |
| *proboscidea* | 18.3 | 19.1 | 6.0 | _ |  |  |  |  |  |  |  |  |  |  |  |  |  |  |  |  |  |
| spA7 | 17.7 | 19.6 | 17.6 | 17.9 | _ |  |  |  |  |  |  |  |  |  |  |  |  |  |  |  |  |
| spA8 | 17.6 | 19.4 | 16.4 | 16.4 | 9.8 | _ |  |  |  |  |  |  |  |  |  |  |  |  |  |  |  |
| *laurini* | 15.8 | 18.4 | 17.1 | 16.6 | 16.1 | 15.6 | _ |  |  |  |  |  |  |  |  |  |  |  |  |  |  |
| spA5 | 15.8 | 17.0 | 15.8 | 15.3 | 12.8 | 12.9 | 9.3 | _ |  |  |  |  |  |  |  |  |  |  |  |  |  |
| spA4 | 15.0 | 17.5 | 16.0 | 16.9 | 14.8 | 12.8 | 14.3 | 12.0 | _ |  |  |  |  |  |  |  |  |  |  |  |  |
| spA3 | 15.5 | 17.2 | 16.0 | 16.2 | 13.2 | 14.2 | 11.0 | 11.1 | 8.6 | _ |  |  |  |  |  |  |  |  |  |  |  |
| *alpestris* | 15.2 | 17.9 | 14.3 | 15.0 | 13.5 | 13.6 | 10.0 | 9.5 | 8.5 | 6.3 | _ |  |  |  |  |  |  |  |  |  |  |
| spA2 | 14.7 | 16.6 | 15.0 | 15.3 | 13.8 | 13.3 | 11.1 | 9.8 | 8.4 | 5.4 | 5.9 | _ |  |  |  |  |  |  |  |  |  |
| *brevicrus* | 14.5 | 16.3 | 15.1 | 15.1 | 13.6 | 12.8 | 10.9 | 9.9 | 8.2 | 5.2 | 5.6 | 2.6 | _ |  |  |  |  |  |  |  |  |
| spA6 | 13.7 | 15.7 | 13.5 | 13.8 | 12.4 | 12.0 | 10.6 | 9.1 | 9.0 | 7.8 | 8.0 | 7.5 | 7.2 | _ |  |  |  |  |  |  |  |
| spA1 | 14.2 | 16.5 | 15.1 | 14.8 | 12.3 | 11.4 | 10.3 | 9.8 | 9.9 | 8.3 | 7.2 | 7.1 | 6.6 | 6.6 | _ |  |  |  |  |  |  |
| *pandanicola* | 14.7 | 17.0 | 16.2 | 16.7 | 11.1 | 11.8 | 11.3 | 10.1 | 10.6 | 9.9 | 8.2 | 9.0 | 8.8 | 9.0 | 5.8 | _ |  |  |  |  |  |
| *darlingtoni* | 12.9 | 16.0 | 14.6 | 15.0 | 11.9 | 11.5 | 10.9 | 9.7 | 7.8 | 8.8 | 6.7 | 8.0 | 7.5 | 7.7 | 5.0 | 3.7 | _ |  |  |  |  |
| *fafniri* | 13.8 | 15.7 | 15.1 | 15.3 | 12.4 | 12.6 | 12.0 | 11.2 | 10.1 | 10.2 | 8.8 | 9.3 | 8.6 | 8.0 | 6.1 | 5.0 | 4.5 | _ |  |  |  |
| *siegfriedi* | 14.2 | 16.2 | 14.0 | 14.0 | 12.1 | 12.1 | 12.0 | 10.9 | 10.1 | 10.2 | 8.1 | 9.5 | 9.0 | 8.5 | 6.0 | 5.1 | 3.6 | 3.4 | _ |  |  |
| *epirrhina* | 16.0 | 18.1 | 15.4 | 14.6 | 18.5 | 18.8 | 18.1 | 16.7 | 16.4 | 15.6 | 16.4 | 15.7 | 15.7 | 15.2 | 15.1 | 14.9 | 14.6 | 14.7 | 13.8 | _ |  |
| *nigrescens* | 16.8 | 18.7 | 14.8 | 14.3 | 17.3 | 17.5 | 16.6 | 16.2 | 16.8 | 15.1 | 16.2 | 16.8 | 16.2 | 14.7 | 15.3 | 15.7 | 15.3 | 15.3 | 15.2 | 7.3 | _ |
| *microps* | 19.5 | 20.4 | 19.9 | 19.0 | 21.5 | 22.6 | 19.1 | 21.3 | 22.6 | 20.4 | 20.0 | 20.9 | 20.6 | 18.7 | 18.6 | 17.6 | 16.9 | 17.0 | 16.5 | 10.5 | 11.0 |
| *amomani* | 20.0 | 21.4 | 18.6 | 17.3 | 22.1 | 21.3 | 18.9 | 20.6 | 22.8 | 20.0 | 19.6 | 19.6 | 19.1 | 16.5 | 17.7 | 18.9 | 18.0 | 17.4 | 18.3 | 13.8 | 14.0 |
| spB1 | 16.9 | 18.9 | 15.3 | 15.0 | 18.8 | 19.2 | 17.8 | 17.3 | 17.2 | 17.0 | 16.7 | 16.2 | 15.7 | 14.5 | 14.8 | 15.0 | 14.4 | 13.9 | 13.7 | 9.0 | 10.9 |
| *arndtorum* | 20.0 | 21.4 | 18.7 | 17.8 | 21.4 | 22.0 | 18.9 | 20.0 | 21.8 | 19.6 | 19.1 | 18.9 | 18.7 | 16.6 | 18.0 | 16.9 | 16.4 | 16.0 | 16.0 | 12.2 | 12.7 |
| *grylloides* | 16.8 | 17.9 | 15.4 | 15.4 | 20.1 | 18.2 | 16.9 | 16.8 | 17.8 | 17.5 | 18.0 | 16.6 | 16.9 | 14.5 | 15.8 | 17.9 | 16.9 | 17.3 | 17.8 | 15.3 | 15.8 |
| *gracilirostris* | 16.6 | 18.3 | 13.5 | 13.8 | 17.0 | 18.1 | 16.5 | 14.7 | 17.1 | 15.8 | 15.5 | 14.7 | 15.2 | 13.6 | 13.8 | 14.9 | 14.1 | 14.4 | 14.8 | 14.0 | 13.3 |
| spB2 | 19.0 | 20.0 | 16.1 | 15.6 | 18.0 | 17.3 | 17.8 | 16.3 | 18.3 | 17.9 | 17.3 | 16.6 | 16.5 | 14.9 | 16.7 | 17.6 | 16.5 | 17.3 | 17.1 | 18.3 | 15.5 |
| spB3 | 16.1 | 18.6 | 13.8 | 13.8 | 13.5 | 14.5 | 14.5 | 12.6 | 13.3 | 13.4 | 13.3 | 12.6 | 13.3 | 12.1 | 12.2 | 12.6 | 12.3 | 13.6 | 13.8 | 13.9 | 13.8 |
| *burtoni* | 16.5 | 17.9 | 14.6 | 14.8 | 15.9 | 15.7 | 14.5 | 13.9 | 15.2 | 14.6 | 14.4 | 13.9 | 13.9 | 12.5 | 12.4 | 12.9 | 12.0 | 13.4 | 13.5 | 15.7 | 13.3 |

**Table S3. Cont’d**

|  | *nigrescens* | *microps* | *amomani* | spB1 | *arndtorum* | *grylloides* | *gracilirostris* | spB2 | spB3 | *burtoni* |
| --- | --- | --- | --- | --- | --- | --- | --- | --- | --- | --- |
| *exclamitans* |  |  |  |  |  |  |  |  |  |  |
| spC1 |  |  |  |  |  |  |  |  |  |  |
| *longirostris* |  |  |  |  |  |  |  |  |  |  |
| *proboscidea* |  |  |  |  |  |  |  |  |  |  |
| spA7 |  |  |  |  |  |  |  |  |  |  |
| spA8 |  |  |  |  |  |  |  |  |  |  |
| *laurini* |  |  |  |  |  |  |  |  |  |  |
| spA5 |  |  |  |  |  |  |  |  |  |  |
| spA4 |  |  |  |  |  |  |  |  |  |  |
| spA3 |  |  |  |  |  |  |  |  |  |  |
| *alpestris* |  |  |  |  |  |  |  |  |  |  |
| spA2 |  |  |  |  |  |  |  |  |  |  |
| *brevicrus* |  |  |  |  |  |  |  |  |  |  |
| spA6 |  |  |  |  |  |  |  |  |  |  |
| spA1 |  |  |  |  |  |  |  |  |  |  |
| *pandanicola* |  |  |  |  |  |  |  |  |  |  |
| *darlingtoni* |  |  |  |  |  |  |  |  |  |  |
| *fafniri* |  |  |  |  |  |  |  |  |  |  |
| *siegfriedi* |  |  |  |  |  |  |  |  |  |  |
| *epirrhina* |  |  |  |  |  |  |  |  |  |  |
| *nigrescens* | _ |  |  |  |  |  |  |  |  |  |
| *microps* | 11.0 | _ |  |  |  |  |  |  |  |  |
| *amomani* | 14.0 | 10.3 | _ |  |  |  |  |  |  |  |
| spB1 | 10.9 | 7.9 | 5.9 | _ |  |  |  |  |  |  |
| *arndtorum* | 12.7 | 7.7 | 6.6 | 4.8 | _ |  |  |  |  |  |
| *grylloides* | 15.8 | 17.6 | 17.2 | 15.4 | 17.1 | _ |  |  |  |  |
| *gracilirostris* | 13.3 | 14.8 | 16.1 | 13.3 | 15.1 | 12.5 | _ |  |  |  |
| spB2 | 15.5 | 20.8 | 19.5 | 16.9 | 20.0 | 16.9 | 16.0 | _ |  |  |
| spB3 | 13.8 | 17.6 | 18.0 | 14.5 | 16.9 | 15.0 | 12.5 | 12.8 | _ |  |
| *burtoni* | 13.3 | 17.1 | 16.9 | 14.5 | 17.1 | 12.3 | 12.5 | 11.7 | 9.3 | _ |

**Table S4.** Summary information of maximum male body size, elevational range and calling height for *Choerophryne* in clades A and B.

|  |  |  |  | Bayetraits | Bayestraits |  | BEAST | BEAST |  |
| --- | --- | --- | --- | --- | --- | --- | --- | --- | --- |
| **Species** | **SUL (mm)** | **log SUL** | **range (m)** | **elevation (m)** | **calling height (cm)** |  | **elevation** | **calling height** | **datasource** |
| *Choerophryne sp B3* | 9.3 | 0.968 | 2275-2300 | 2300 | 10 |  | M | t | This study |
| *Choerophryne grylloides* | 12.5 | 1.097 | 400-900 | 700 | 30 |  | H | t | Iannella et al. 2015 |
| *Choerophryne sp B1* | 12.7 | 1.104 | 1600-1800 | 1700 | 10 |  | L | t | This study |
| *Choerophryne sp A2* | 13.38 | 1.126 | 2500* | 2500 | 10 |  | M | t | This study |
| *Choerophryne burtoni* | 14.1 | 1.149 | 1630-2037 | 1800 | 50 |  | L | s | This study, Richards et al. 2007 |
| *Choerophryne gracilirostris* | 14.7 | 1.167 | 213-1368 | 800 | 20 |  | H | t | Iannella et al. 2014 |
| *Choerophryne sp A3* | 14.7 | 1.167 | 1700* | 1700 | 50 |  | L | s | This study |
| *Choerophryne arndtorum* | 14.8 | 1.167 | 550-650 | 600 | 120 |  | H | t | Günther 2008 |
| *Choerophryne eppirhina* | 15 | 1.176 | 850* | 800 | 20 |  | H | t | Iannella et al. 2015 |
| *Choerophryne amomani* | 15.1 | 1.179 | 1050-1100 | 1100 | 30 |  | L | t | Günther 2008 |
| *Choerophryne sp A6* | 15.49 | 1.190 | 1300-1700 | 1500 | 200 |  | L | s | This study |
| *Choerophryne rostellifer* | 15.6 | 1.193 | 100-1320 | 700 | 300 |  | H | s | This study |
| *Choerophryne sp A5* | 16.4 | 1.215 | 1200* | 1200 | 300 |  | L | s | This study |
| *Choerophryne sp A8* | 16.63 | 1.221 | 1300-1700 | 1500 | 200 |  | L | s | This study |
| *Choerophryne laurini* | 17.1 | 1.233 | 800-950 | 900 | 300 |  | L | s | Günther 2000 |
| *Choerophryne sp A7* | 17.1 | 1.233 | 213-1300 | 700 | 300 |  | H | s | This study |
| *Choerophryne microps* | 17.3 | 1.238 | 380-900 | 600 | 100 |  | H | t | Günther 2008 |
| *Choerophryne brevicrus* | 17.7 | 1.248 | 2117-2900 | 2500 | 30 |  | M | t | Günther and Richards 2011 |
| *Choerophryne sp B2* | 17.7 | 1.248 | 300* | 300 | 30 |  | H | s | This study |
| *Choerophryne nigrescens* | 17.9 | 1.253 | 5-820 | 400 | 250 |  | H | s | Günther 2008 |
| *Choerophryne sp A4* | 18.6 | 1.270 | 1600-1800* | 1700 | 200 |  | L | s | This study |
| *Choerophryne pandanicola* | 19.6 | 1.292 | 2900* | 2900 | 300 |  | M | s | Günther and Richards 2011 |
| *Choerophryne proboscidea* | 19.67 | 1.294 | 100-854 | 400 | 200 |  | H | s | This study |
| *Choerophryne darlingtoni* | 19.84 | 1.298 | 1600-2000 | 1800 | 200 |  | L | s | This study |
| *Choerophryne seigfreidi* | 20.7 | 1.315 | 2500* | 2500 | 200 |  | M | s | This study |
| *Choerophryne sp A1* | 20.89 | 1.320 | 2060-2275 | 2100 | 400 |  | M | s | This study |
| *Choerophryne alpestris* | 22.6 | 1.354 | 3100-3500 | 3300 | 30 |  | U | t | Günther and Richards 2011, Kraus 2010 |
| *Choerophryne longirostris* | 23.7 | 1.375 | 950-1200 | 1100 | 200 |  | L | s | This study, Kraus and Allison 2001 |
| *Choerophryne fafniri* | 25.6 | 1.409 | 2060-2300 | 2200 | 200 |  | M | s | This study |

Supplementary references used to generate table S4.

Günther R. (2000) *Albericus laurini* species nova, the first record of the genus Albercus (Anura, Microhylidae) from the west of New Guinea. *Mitteilungen aus dem Museum fur Naturkunde in Berlin Zoologische Reihe*, **76**, 167–174.

Günther R. (2008) Descriptions of four new species of *Choerophryne* (Anura, Microhylidae) from Papua Province, Indonesian New Guinea. *Acta Zoologica Sinica*, **54**, 653–674.

Gunther R. & Richards S.J. (2011) Five new microhylid frog species from Enga Province, Papua New Guinea, and remarks on *Albericus alpestris* (Anura, Microhylidae). *Vertebrate Zoology*, **61**, 343–372.

Iannella A., Oliver P., & Richards S. (2015) Two new species of *Choerophryne* (Anura, Microhylidae) from the northern versant of Papua New Guinea’s central cordillera. *Zootaxa*, **4058**, 332–340.

Iannella A., Richards S., & Oliver P. (2014) A new species of *Choerophryne* (Anura, Microhylidae) from the central cordillera of Papua New Guinea. *Zootaxa*, **3753**, 483–493.

Kraus F. & Allison A. (2010) A Review of the Endemic New Guinea Microhylid Frog Genus *Choerophryne*. *Herpetologica*, **57**, 214–232.

Richards S.J., Dahl C.S., & Hiaso J. (2007) Another new species of *Choerophryne* (Anura: Microhylidae) from Southern Highlands Province, Papua New Guinea. *Transactions of the Royal Society of South Australia*, **131**, 135–141.

Supplementary Figures


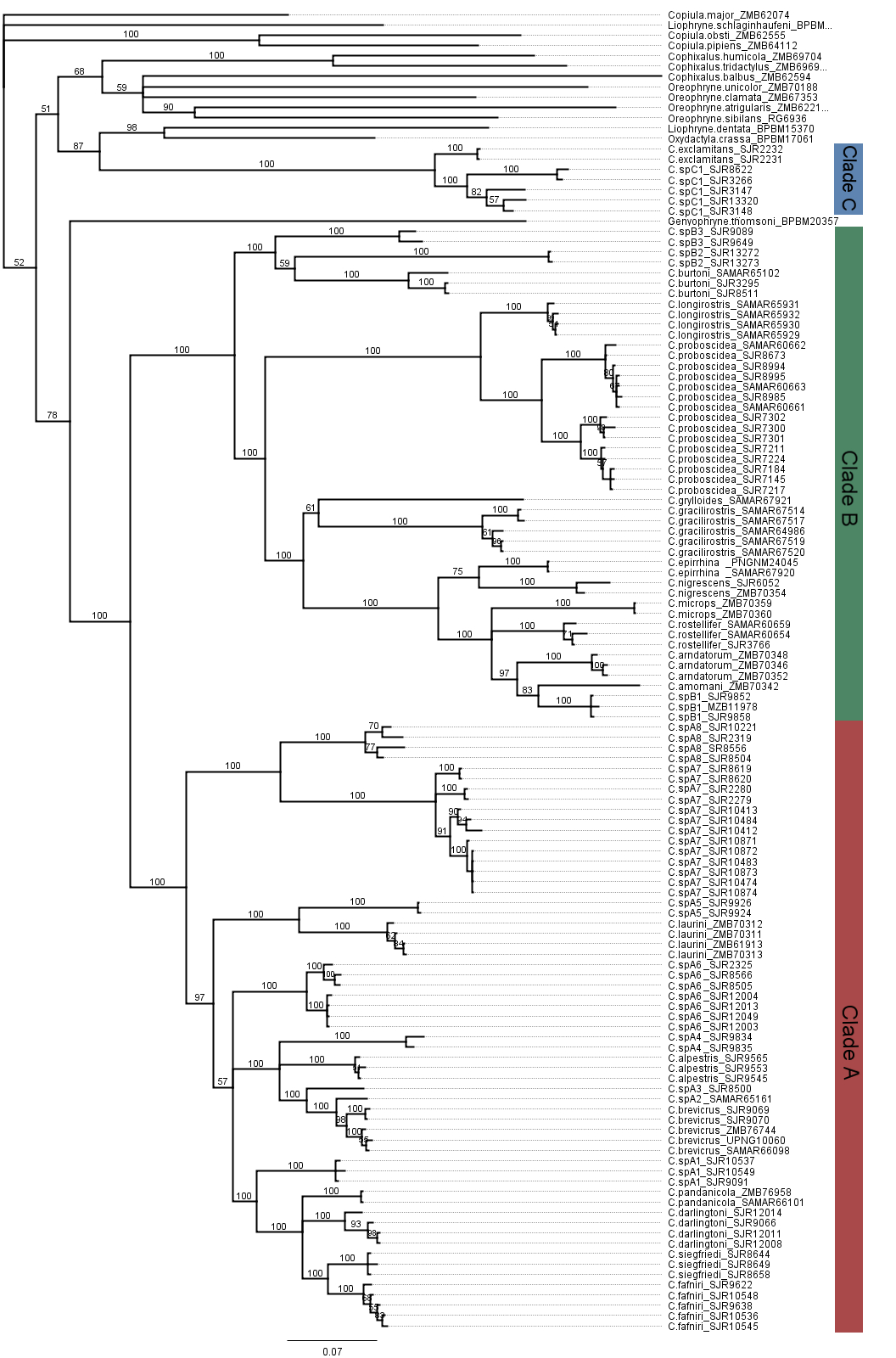


**Figure S1. Summary Bayesian Tree.**

posterior probabilities for all *Choerophryne* from which sequence was obtained, plus selected outgroups.


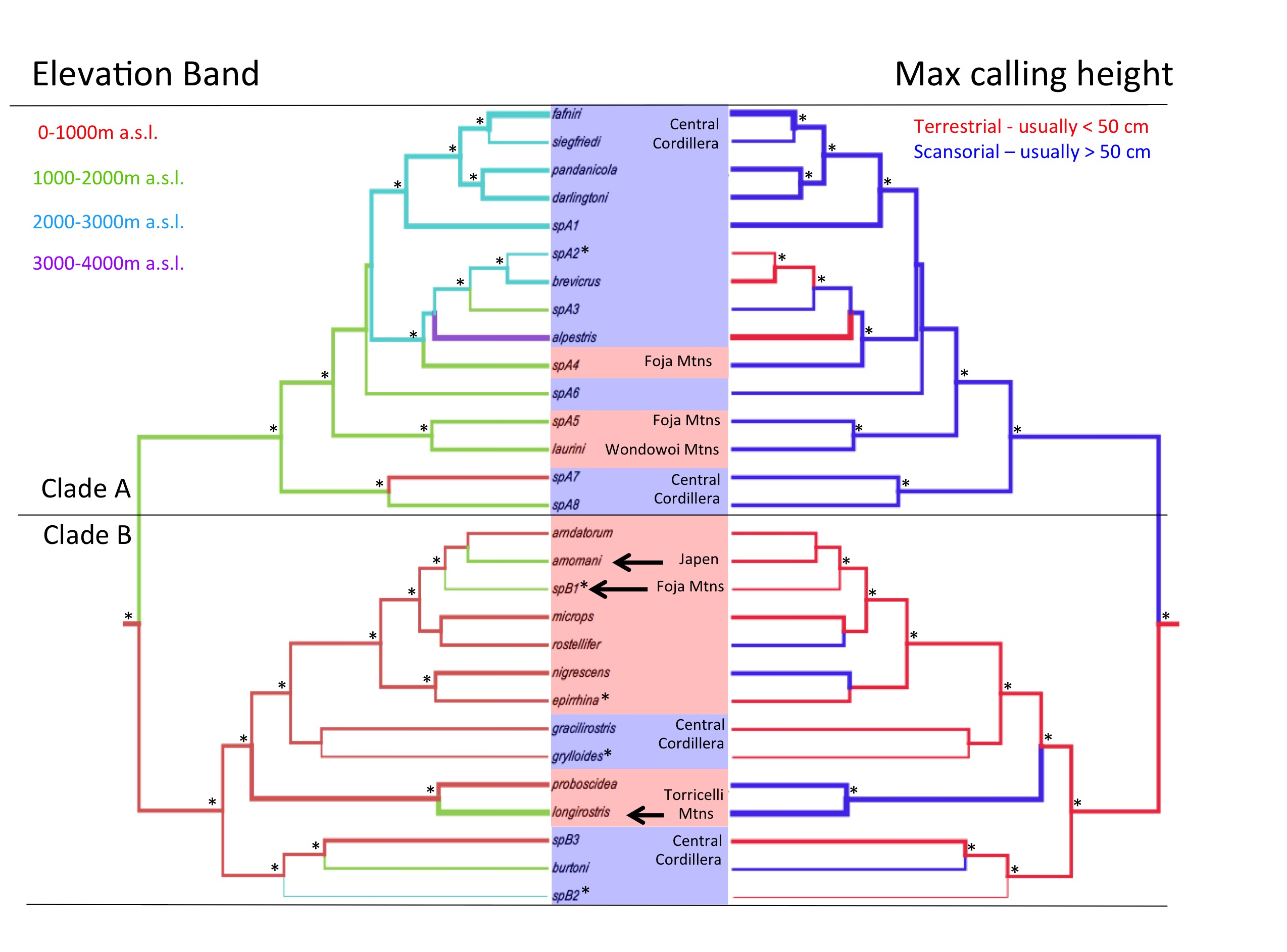


**Figure S2. Trait evolution in the major lineages of *Choerophryne.***

Estimated using BEAST based on discrete character codings. Ancestral states with a probability of greater than 60% are indicated with an asterisk. Branch widths on both trees are proportional to maximum recorded adult male SVL and very small taxa (<15mm) are indicated with bold and an asterisk. Taxa with distributions in the Central Cordillera are highlighted in blue, while those from northern New Guinea are in red. Three inferred upslope shifts in Clade B into lower montane forest in the North Papuan Mountains are indicated by arrows.


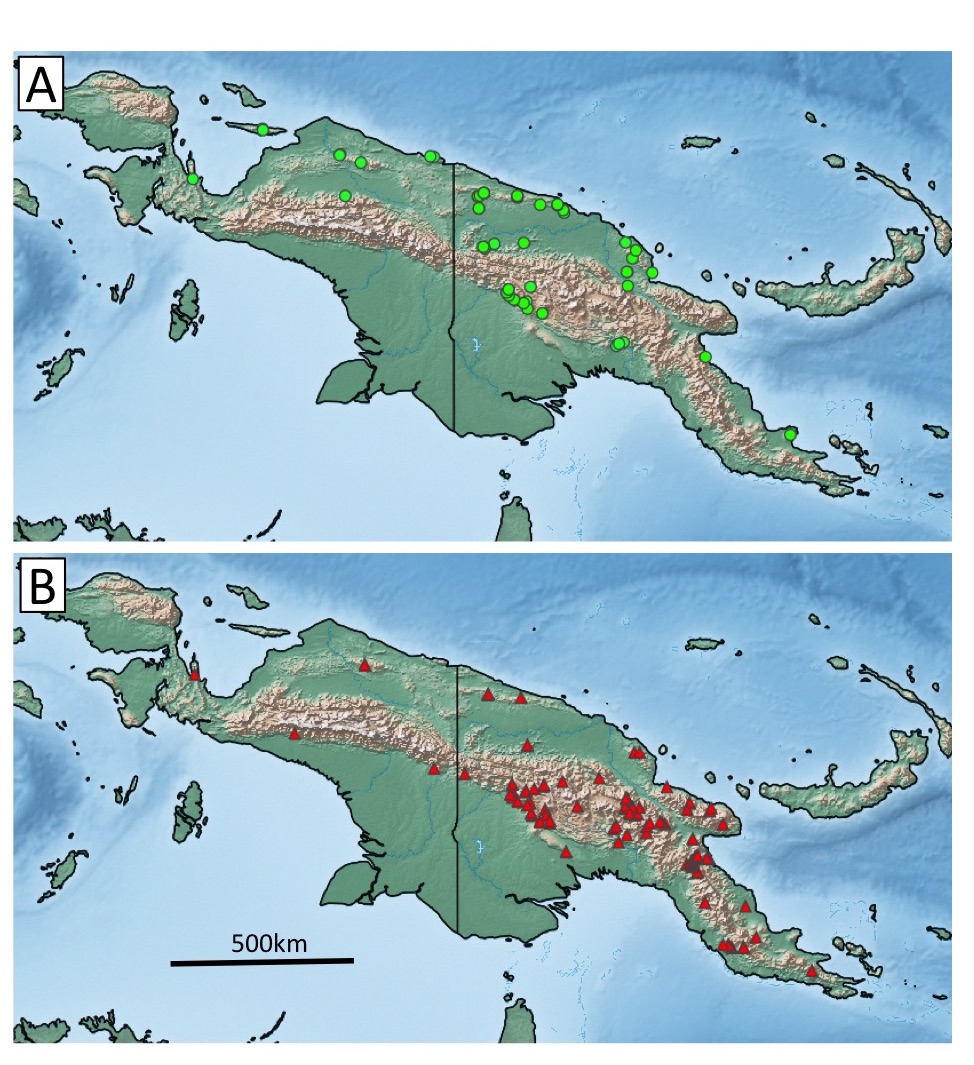


**Figure S3.** **Summary of museum records for *Choerophryne* grouped by phenotype.**

A) taxa with an extended maxillary process (in large part corresponding to Clade B, but see Kraus, 2013 for a probable exception from Milne Bay Province), and B) taxa lacking an extended maxillary process, rendering them morphologically consistent with genotyped samples in Clade A and C.
