# Supplementary material for: Mountain colonisation, miniaturisation and ecological evolution in a radiation of direct-developing New Guinea Frogs (Choerophryne, Microhylidae)
Source: PeerJ. 2017 Mar 30;5:e3077. doi: 10.7717/peerj.3077 (PMC5376113; doi:10.7717/peerj.3077)
Supplement: Appendix S2 — BEAST input file for ancestral state analyses [file peerj-05-3077-s002.docx]

*Journal of Biogeography*

**SUPPORTING INFORMATION**

**Mountain uplift, miniaturisation and diversification in New Guinea Frogs (Choerophryne, Microhylidae)**

Paul M. Oliver, Amy Iannella, Stephen J. Richards and Michael S.Y. Lee

**Appendix S.** BEAST xml file for state evolution analyses (convert to xml format to run)

<?xml version="1.0" standalone="yes"?>

<beast>

<!-- The list of taxa to be analysed (can also include dates/ages). -->

<!-- ntax=45 -->

<!-- All taxa MUST have size values ie no missing data -->

<taxa id="taxa">

<taxon id="Copiula_obsti">

<attr name="size">

1.342

</attr>

</taxon>

<taxon id="Copiula_pipiens">

<attr name="size">

1.398

</attr>

</taxon>

<taxon id="Cophixalus_balbus">

<attr name="size">

1.447

</attr>

</taxon>

<taxon id="Oreophryne_atrigularis">

<attr name="size">

1.477

</attr>

</taxon>

<taxon id="Liophryne_dentata">

<attr name="size">

1.602

</attr>

</taxon>

<taxon id="Oreophryne_sibilans">

<attr name="size">

1.362

</attr>

</taxon>

<taxon id="Liophryne_schlaginhaufeni">

<attr name="size">

1.580

</attr>

</taxon>

<taxon id="Oreophryne_unicolor">

<attr name="size">

1.398

</attr>

</taxon>

<taxon id="Genyophryne_thomsoni">

<attr name="size">

1.602

</attr>

</taxon>

<taxon id="Cophixalus_humicola">

<attr name="size">

1.210

</attr>

</taxon>

<taxon id="Oreophryne_clamata">

<attr name="size">

1.380

</attr>

</taxon>

<taxon id="A_exclamitans">

<attr name="size">

1.314

</attr>

</taxon>

<taxon id="C_sp_nov1">

<attr name="size">

1.204

</attr>

</taxon>

<taxon id="Cophixalus_tridactylus">

<attr name="size">

1.211

</attr>

</taxon>

<taxon id="Copiula_major">

<attr name="size">

1.633

</attr>

</taxon>

<taxon id="Oxydactyla_crassa">

<attr name="size">

1.477

</attr>

</taxon>

<taxon id="C_foyas_2">

<attr name="size">

1.215

</attr>

</taxon>

<taxon id="C_laurini">

<attr name="size">

1.233

</attr>

</taxon>

<taxon id="C_tuberculus">

<attr name="size">

1.233

</attr>

</taxon>

<taxon id="C_sp4">

<attr name="size">

1.221

</attr>

</taxon>

<taxon id="C_sp_juha">

<attr name="size">

1.320

</attr>

</taxon>

<taxon id="C_sp3">

<attr name="size">

1.190

</attr>

</taxon>

<taxon id="C_darlingtoni">

<attr name="size">

1.298

</attr>

</taxon>

<taxon id="C_pandanicolus">

<attr name="size">

1.292

</attr>

</taxon>

<taxon id="C_fafniri">

<attr name="size">

1.409

</attr>

</taxon>

<taxon id="C_foyas_1">

<attr name="size">

1.270

</attr>

</taxon>

<taxon id="C_alpestris">

<attr name="size">

1.354

</attr>

</taxon>

<taxon id="C_moranpeep">

<attr name="size">

1.167

</attr>

</taxon>

<taxon id="C_brevicrus">

<attr name="size">

1.248

</attr>

</taxon>

<taxon id="C_Muller_tiny">

<attr name="size">

1.126

</attr>

</taxon>

<taxon id="C_longirostris">

<attr name="size">

1.375

</attr>

</taxon>

<taxon id="C_proboscidea_1">

<attr name="size">

1.294

</attr>

</taxon>

<taxon id="C_gracilirostris">

<attr name="size">

1.167

</attr>

</taxon>

<taxon id="C_burtoni">

<attr name="size">

1.149

</attr>

</taxon>

<taxon id="C_purari">

<attr name="size">

1.248

</attr>

</taxon>

<taxon id="C_porgera">

<attr name="size">

0.968

</attr>

</taxon>

<taxon id="C_grylloides">

<attr name="size">

1.097

</attr>

</taxon>

<taxon id="C_microps">

<attr name="size">

1.238

</attr>

</taxon>

<taxon id="C_epirrhinos">

<attr name="size">

1.176

</attr>

</taxon>

<taxon id="C_nigrescens">

<attr name="size">

1.253

</attr>

</taxon>

<taxon id="C_sp_foyas">

<attr name="size">

1.104

</attr>

</taxon>

<taxon id="C_rostellifer">

<attr name="size">

1.193

</attr>

</taxon>

<taxon id="C_amomani">

<attr name="size">

1.179

</attr>

</taxon>

<taxon id="C_arndatorum">

<attr name="size">

1.167

</attr>

</taxon>

<taxon id="C_siegfriedi">

<attr name="size">

1.315

</attr>

</taxon>

</taxa>

<!-- Liophryne_schlaginhaufeni is sister to all other taxa according to Peloso et al. 201 -->

<taxa id="Ingroup">

<taxon idref="C_sp4"/>

<taxon idref="C_foyas_2"/>

<taxon idref="C_epirrhinos"/>

<taxon idref="C_grylloides"/>

<taxon idref="C_fafniri"/>

<taxon idref="C_purari"/>

<taxon idref="C_longirostris"/>

<taxon idref="A_exclamitans"/>

<taxon idref="C_pandanicolus"/>

<taxon idref="C_rostellifer"/>

<taxon idref="C_nigrescens"/>

<taxon idref="C_darlingtoni"/>

<taxon idref="C_brevicrus"/>

<taxon idref="C_siegfriedi"/>

<taxon idref="C_tuberculus"/>

<taxon idref="C_sp_juha"/>

<taxon idref="C_moranpeep"/>

<taxon idref="C_sp3"/>

<taxon idref="C_burtoni"/>

<taxon idref="C_sp_foyas"/>

<taxon idref="C_proboscidea_1"/>

<taxon idref="C_gracilirostris"/>

<taxon idref="C_foyas_1"/>

<taxon idref="C_Muller_tiny"/>

<taxon idref="C_porgera"/>

<taxon idref="C_sp_nov1"/>

<taxon idref="C_alpestris"/>

<taxon idref="Liophryne_dentata"/>

<taxon idref="Oxydactyla_crassa"/>

<taxon idref="Genyophryne_thomsoni"/>

<taxon idref="Oreophryne_sibilans"/>

<taxon idref="Copiula_major"/>

<taxon idref="Oreophryne_atrigularis"/>

<taxon idref="Copiula_obsti"/>

<taxon idref="Cophixalus_balbus"/>

<taxon idref="Copiula_pipiens"/>

<taxon idref="Oreophryne_clamata"/>

<taxon idref="Cophixalus_tridactylus"/>

<taxon idref="Cophixalus_humicola"/>

<taxon idref="Oreophryne_unicolor"/>

<taxon idref="C_laurini"/>

<taxon idref="C_amomani"/>

<taxon idref="C_arndatorum"/>

<taxon idref="C_microps"/>

</taxa>

<!-- core Albericus only -->

<taxa id="Albericus">

<taxon idref="C_sp4"/>

<taxon idref="C_foyas_2"/>

<taxon idref="C_fafniri"/>

<taxon idref="C_pandanicolus"/>

<taxon idref="C_darlingtoni"/>

<taxon idref="C_brevicrus"/>

<taxon idref="C_siegfriedi"/>

<taxon idref="C_tuberculus"/>

<taxon idref="C_sp_juha"/>

<taxon idref="C_moranpeep"/>

<taxon idref="C_sp3"/>

<taxon idref="C_foyas_1"/>

<taxon idref="C_Muller_tiny"/>

<taxon idref="C_alpestris"/>

<taxon idref="C_laurini"/>

</taxa>

<taxa id="Choerophryne">

<taxon idref="C_epirrhinos"/>

<taxon idref="C_grylloides"/>

<taxon idref="C_purari"/>

<taxon idref="C_longirostris"/>

<taxon idref="C_rostellifer"/>

<taxon idref="C_nigrescens"/>

<taxon idref="C_burtoni"/>

<taxon idref="C_sp_foyas"/>

<taxon idref="C_proboscidea_1"/>

<taxon idref="C_gracilirostris"/>

<taxon idref="C_porgera"/>

<taxon idref="C_amomani"/>

<taxon idref="C_arndatorum"/>

<taxon idref="C_microps"/>

</taxa>

<!-- ordered ULH -->

<generalDataType id="alt_1">

<state code="U"/> <!-- upper montane -->

<state code="M"/> <!-- mid montane -->

<state code="L"/> <!-- lower montane -->

<state code="H"/> <!-- hill forest -->

<ambiguity code="?" states="ULH"/>

</generalDataType>

<generalDataType id="ecology">

<state code="S"/> <!-- scansorial -->

<state code="T"/> <!-- terrestrial -->

<ambiguity code="?" states="ST"/>

</generalDataType>

<!-- The 12S-16S sequence alignment (each sequence refers to a taxon above). -->

<!-- ntax=45 nchar=1347 -->

<alignment id="alignment1" dataType="nucleotide">

<sequence>

<taxon idref="Copiula_obsti"/>

-------------------------------------GCCGGGAACTAC--CCAGCTTAAAACCCAAAGGACTTGACGGTGCCCCATCCCCCTAGAGGAGCCTGTTCTACAATCGATAACCCCCGATCAACCTCACCCCTTCTTGAATATCAGCCTGTATACCTCCGTCGCAAGCCCGTCATGTGAATGCATCAACGGACCCAACGATTCCATCACCACGTCAGGTCAAGGTGCAGCTTATGAAGCGGAATGAAATGGGCTACAATTTCTACTAGAACATACGGAACACTATGTGCAATATAGTCAC--AAGGCGGATTTAGCAGTAAAAAGAAAATAGAGAGTTCTTTTTAATTAGGCAATGAGGCGCGTACACACCGCCCGTCACCCTCTTCAAATAATGTCACTAACTTCTCCCTGTCCAAAGAAGAGGCAAGTCGTAACACGGTAAGCGTACTGGAAGGTGTGCTTGGAACAAAATGTAACTTAACAAAAGTATCTTGCTTACACCAAGAATATGTCCGTAAAACTCCGACCATTTTGAGCTCAAAATCTAGCCCTAACCCAAATAACAACACACCCCTAAAAATACTATAAAACATTTTATTAACTTAGTATAGGCGATCAAAAAGTTTCTAGGAG-----------------------------------------------------------------------------------------------------------------------------------------------------------------------------------------------------------------------------------------------------TAGAGATAGCTGGTTATTCAGGAAAAGGATATAGTCCAACTTTAATTCATACTATCAAATACACCCAGATTTAAAATTTATTCAAATAAGGTTCAGCCTATTTGAAACAGGATACAACCTACACTACTAGGTAATGATGTACCGTGATCAAGTAGGCCTAAAAGCAGCCATCTTCAAAAAAGCGTCACAGCTTCATCACTATAACTAATTTTAACCGAACCCCAAAACCTTTAATACTGAATGATTCCATACTACTATGGAAAACCTTATGCTAGAACTAGTAACAAGAATAATTCTCCTAACGTAAACGTAA-CCGGA-----CTTTTCCGTATCTACCGCTAATGAGCTCAAAGTAGTAACCACAAGAAAACCCTACTTTCTAAAGCGTTAACCTTACACTAGAACTTTTCTGGAAGATTAAAAAGAAGGGAAGGAACTCGGCAAAACTAACCCCGCCT

</sequence>

<sequence>

<taxon idref="Copiula_pipiens"/>

------------------------------------GCCAGGAAATTACGCCAAGCTTAAAACCCAAAGGACTTGACGGTGCCCCATCCCCCTAGAGGAGCCTGTTCTATAATCGATAATCCCCGATACACCTCACCACTTCTTGAACATCAGCCTGTATACCTCCGTCGCAAGCCCGCCATGTGAACGCATCAGCGGACCCAACGATTTCATCATTACGTCAGGTCAAGGTGCAGCCCATGAAGTGGAAAGAAATGGGCTACAATCTCTACTAGAACAAACGGAAAACTACATGCAACATAGTAAC--AAGGCGGATTTAGTAGTAAAAA-AAAATAGAGAATCTTTTTTAACCCGGCACTGAGGTGCGTACATACCGCCCGTCACCCTCTTCAAACAACGTTATTAACTTCTTTCCGTCCACAGAAGAGGCAAGTCGTAACACGGTAAGCGTACCGGAAGGTGCGCTTGGAACAAAATGTAACTTAACAAAAGTATCTTGCTTACACCAAGAGTATGTCCGTGAAACTCTGACCATTATGAGCTCAAAGCCTAGCCCTATCAAATTGAATATAACTACCTAACAAATATAATAAAACATTTTATTAACTCAGTATAGGCGATTAAAAAGTTTCTAGAAG-----------------------------------------------------------------------------------------------------------------------------------------------------------------------------------------------------------------------------------------------------TAGAGATAGCTGGTTATTCAGGAAAAGGATATAGTCCAACTTTAACCCATAATACCAAATACACCTAGATTTAAAATTTATTCAAATAAGGTTCAGCCTATTTGAAACAGGATACAACCTATACTATCAGGTAATGTTTTAACACGATCAAGTAGGCCTCACAGCAGCCACCTTTAAAAAAGCGTTAAAGCTTCAT-CCCTACCCTAATACCCATCAAAACCCAAAACCTATAATACTGAATGATTTTATA-TGTTATAAAAACCCTTATGTTAGAACTAGTAACAAGAATAATTCTCCCAAAGTAAGTGTAA-----------ACCACTCACACTTATCGACAATGAATATAAAGTAGTAACCACAAGAAAAACCTACTCTCTTAGCCGTTAACCTTACACTAGAACCTTTCCGGAAGATTAAAAAGAGGGGAAGGAACTCGGCAAAATTAACCCCGCCT

</sequence>

<sequence>

<taxon idref="Cophixalus_balbus"/>

------------------------------------GCCAGGGAATTACGCCAAGCTTAAAACCCAAAGGACCTGACGGTACCCCATCCCCCTAGAGGAGCCTGTTCTATAATCGATAATCCCCGATACACCTCACCCCCTCTTGAATTTCAGCCTGTATACCTCCGTCGCAAGCCTACCATGTGAATGCAACAGTAAGCCTAAAGACCACATACATACGTCAGGTCAAGGTGCAGCCCATGAGGCGGGAAGAAATGGGCTACAATTTCTAATAGAACAAACGGAAGACTGCATGCAACACAGTCAC--AAGGCGGATTTAGCAGTAAAAAGAAACCAGAGAGTTCTTTTTAACCCGGCCCTGGGGTGTGTACACACCGCCCGTCACCCTCTTCATGAATGTTCCCTAACCCCCCCAAA--AACAGAAGAGGCAAGTCGTAACACGGTAAGCGTACTGGAAAGTGCGCTTGGAACAAAATGTAACTTATAAAAAGTATCTTGCTTACACCGAGAAGATGTCTACGAAATTTAGACCATTTTGCGCTAACTGCCCAGCCCCTACTCATGTAAATCAAACAACAATT--TTATAATAAAACATTCTGTGACCCA-GTAAAGGCGATTAAAAAGTATGTAGGAG-----------------------------------------------------------------------------------------------------------------------------------------------------------------------------------------------------------------------------------------------------TAGAGATAGCTGGTTATTCAGGAAAAGGATAAAGTCCAACCTTAAATGATACTCTCAAATACAACTAGATTTAAGTTTCACTCAAATAGGGTACAGCCTATTTGAAACAGGGTACAACCTACAACACAGGGTAATGCCCCTGTGTGACAAAGTGGGCTTAATAGCAGCCACCTTTTAAAAAGCGTTACAGCCTTACCACCTTCACTAATCCGACCCTAAACCCAAAACCCACCGTACTGAATGACTCCATATAATTATGGAAGACCTTATGTTAGAACTAGTAATAAGAATAATTCTCAAAACGTAAGTGTAAGCCGGGTTGAACTCACCGGCACTCACCGCCAATGAACCTAAAGTAACAACTACAAGAAAACCTTACTCATTCATGCGTTAACCTTACACCAGAGCTTTTTCGGAAGATTATAAGAGTGGGAAGGAACTCGGCAAATTTAACCCCGCCT

</sequence>

<sequence>

<taxon idref="Oreophryne_atrigularis"/>

------------------------------------GCCAGGGAACTACGGCAAGCTTAAAACCCAAAGGACTTGACGGTGTCCCACCTACCTAGAGGAGCCTGTTCTATAATCGATAATCCCCGATACACCCCACCTCCCCTCGCAGCCCAGCCTGTATACCTCCGTCGCAAGCTTACCGCCTGAGCGCCCCAGTAAGCTAAAAGATCACATCCACACGTCAGGTCAAGGTGCAGCCTATGGGGCGGAAAGAAATTGGCTACAATTTCTATTAGAACACACGGAAAGCTGT-TGCAACACAGCTC-TGAAGGCGGATTTAGAAGTAAAAAGAAAATAGAGAGTTCTTTTTAAACTAGCACTGGGACATGTACACACCGCCCGTCACCCTCTTCAACATG--TCCTTAACACCTTAAACCTAGAAGAAGAGGCAAGTCGTAACATGGTAAGTGTACTGGAAAGTGCACTTGGGACAAAATGTAACTTAACAAAAGTACTTTGCTTACACCAAAGACATGTCTGTGAAACCCGGACCATTCTGAGCTTAAAACCCAGCCCCTCCAAACAAA---------------CAAACAAATAAAACATTTTCTCACCTA-GTAAAGGCGATTGAAAAGTACTTAGGAG-----------------------------------------------------------------------------------------------------------------------------------------------------------------------------------------------------------------------------------------------------TAGAGATAGCTGGTTACTTAGGAAAAGGATTTAGTCCAACTTTAAATCATGATACCAAACAAACTTAGATTTAAATTTTAATCAAATAAGGTACAGCTTATTTGACACAGGATACAGCCTATAATACCGGGTAATGTTTATTCATGACCAAGTGGGCCTAAGAGCAGCCACCTTTTAAAACGCGTTACAGCCTAATCACTCCTAATAATCCCAATTTAAACCCAAAACCCTCCACACTAAGTGACTCCATACCAATATGGAAGACCTTATGTTAGAACTAGTAACAAGAGCAAGCCTCTACAAGCAAGTGTAAGCCGGATCGAA-TCACCGGCATTTACCGCCAATGAACTACTAGTAGCAACCACAAGAAAACCCTACTCACCACAGCGTTAACCTTACACTAGAACTTCCCAGGAAGATTAAAAAAGAAGGAAGGAACTCGGCAAACTTAACCTCGCCT

</sequence>

<sequence>

<taxon idref="Liophryne_dentata"/>

------------------------------------GCCTGGAAATTACGTCAAGCTTAAAACCCAAAGGACTTGACGGTGTCCCATCCCCCTAGAGGAGCCTGTTCTATAATCGATTTTCCCCGATTTACCTCACCACCCCTTGAAACTCAGTCTGTATACCTCCGTCGCAAGCTTACCATGTGAACGCATTAGTGAGCTTAAAAATTTTATTTACACGTCAGGTCAAGGTGCAGCCCATGGGCTGGAAAGAAATGGGCTACAGTTTCTATTAGAACAAACGAAAAACTACATGCAATATAGCTATTGAAGGCGGATTTAGAAGTAAAAAGAAAACAGAGAGTTCTTTTTAATTGGGCTCTGGGACGCGTACACACCGCCCGTCATCCTCTTCATAATG--TTACTAACCTAATAAAATCTTCAGAAGAGGCAAGTCGTAACACGGTAAGCGTACTGGAAAGTGTGCTTGGAACAAAATGTAACTTAACAAAAGTATCCTGCTTACACCAAGAATATGTCTGTGAAACTCTGACCATTTTGAGCCAAAAACCTAGCCCTATCAAATGATTTAATTCTACCTGACAAAACTAATAAAACATTTTTAATTCCTAGTAAAGGTGATTAAAAAGTATTTAGGAG-----------------------------------------------------------------------------------------------------------------------------------------------------------------------------------------------------------------------------------------------------TAGAGATAGCTGGTTATTCAGAAAAAGGATACAGTCCAAATTTAAATTATACAACCAAATACCACAAGATTTAAAATTCATTCAAATAAGGTACAGCCTATTTGAAATAGGGTACAACCTTGAACATCGGGTAATACTTTTACGTGACCAAGTAGGCCTAAAAGCAGCCACCTTTAAAAAAGCGTTATAGCCTCATCACAATAATTAATCCCAATATAAAATTAAAACCCCTCTTACTGAATGACTCCATACTTTTATGGAAAACCTTATGTTAGAACTAGTAACAAGAATATTTCTCTCAAAGTAAGCATATGCCAGAATGAACTCACTGGCACTTACCGTCAATGAATATAAAGTAATAACTTCAAGAAAACATTACTCTTCTTA-CGTTAACCTTACACTAGAACTTTTCTGGAAGATTAAAAAAGGGGGAAGGAACTCGGCAAAACTGACTCCGCCT

</sequence>

<sequence>

<taxon idref="Oreophryne_sibilans"/>

-------------------------------------GCCGGGAATTACGCCAAGCTTAAAACCCAAAGGACTTGACGGTGTCCCATCCCCCTAGAGGAGCCTGTTCTATAATCGATACCCCCCGCTATACCTCACCGCCCCTTGCAACTCAGCCTGTATACCTCCGTCGCAAGCTTACTATGTGAGTATTCCAGTCAGCCTAAAGGTCTCACCAACACGTCAGGTCAAGGTGCAGCCCATGAGACGGAAAGAAATGGGCTACAATTTCTACTAGAACAAACGAAAGACTGCATGAAACACAGTCATTGAAGGCGGATTTAGCAGTAAAAAGAAAATAGAGAGTTCTTTTTAACTGGGCACTGGGACGCGTACACACCGCCCGTCACCCTCTTTGATAAACGTCATTAACACTATATAACTAAAAAAAGAGGCAAGTCGTAACATGGTAAGCGTACTGGAAAGTGCTCTTGGAACAAAATGTAGCTTAACTAAAGCATCCTGCTTACACCAAGAACATGCCTGT?AAACTCGGACCATTTTGAGCTCA?AACCTAGCCCCTCCCAATGAATCTTACAACATAAAT------AATAAAACATTTTTTTACTTA-GTAAAGGCGATCAAAAAGTACTTAAAAG-----------------------------------------------------------------------------------------------------------------------------------------------------------------------------------------------------------------------------------------------------------------------------------CAAGT-CAACTTTAAATAATAACACCAAATAAACTAAGATTTAAATTTAATTCAAGCAGGGCACAGCCTACTTGACACAGGATACAACCTCCAAGATAGGGTAATGTTTTCACGTGACCAAGTAGGCCTAAGAGCAGCCACCTTTCAAAAAGCGTTACAGCCTCATCACCCCCACCCATACCAACTAAAACCCAAAACCCATACTACTGAATGACTCCATAATAATATGGAAAACCCAATGTTAAAACTAGTAACAAGAACAATTCTTACAAAGTAAGTGTAAGCCGGACTGAACCCACCGGCACTCACCGTCAATGAATTCATAGTAGCAACCACAAGAAAACCCTACTTACTCCAACGTTAACCTTACACTAGAACTTTCCAGGAAGATTAAAAAAAGGGGAAGGAACTCGGCAAAATTAACCCCGCCT

</sequence>

<sequence>

<taxon idref="Liophryne_schlaginhaufeni"/>

------------------------------------GCCAGGGTATTACGCCAAGCTTAAAACCCAAAGGACTTGACGGTGTCCCATCCCTCTAGAGGAGCCTGTTCTATAATCGATTACCCACGATTTACCTTACCCCTTCTTGAAATTCAGTCTGTATACCTCCGTCGCAAGCTTACCCTGTGAACGCATTAGTGAGCCAAAAGATCTCATCCATACGTCAGGTCAAGGTGCAGCCCACGAAGGGGGAAGAAATGGGCTACAATCTCTACTAGAACAAACGGAAGATTACATGAAACACAAATCC-CAAGGTGGATTTAGTAGTAAAAAGAAAGCAGAGCATTCTTTTTAATCAGGCGCTGGGACGCGTACACACCGCCCGTCACCCTCCTCAAAATG--TTAATAACCCCATATAATTTATAGAAGAGGCAAGTCGTAACATGGTAAGCGTACTGGAAAGTGCGCTTGGAACAAAATGTAGCTTAATAAAAGCATCCTGCTTACACCAGGACTATGTCTGTGAAACTCTAACCATTTTGAGCTAAAAGTTTAGCCCATTCTAATAAAACAAGCCCATTAAACTAAACAAATAAAACATTTTCTTAACCCAGTAAAGGCGATTAAAAAGCATTTAGGAG-----------------------------------------------------------------------------------------------------------------------------------------------------------------------------------------------------------------------------------------------------TAGAGATAGCTGGTTATTTAGGAAACGAATCTAGTTCTACTTTAAACTATAA-TCTCAATATAATTAGATTTAAAATTTATTCAAATAAGGTACAGCCTATTTGAAAAAGGATACAACCTACAAGATTGGGTAATATAACTATATAACCAAGTGGGCCTAAAAGCAGCCACCTTTTAAAAAGCGTCATAGCTTTTCTACAATTACTAATACTGATTTAAACTCAAAACCCCCAATACTAAATGACTCCATA-CCATATGGAAAACCTTATGTTAGAACTAGTAACAAGAATTTTTCTCACAAAGTGAATGTAAGCCGAAACGAACTCACCGGCATTTATCGCCAACGAATCTACAGTAACAACTACAAGAAAATCTTACTCTTTCAAGCGTTAACCTTACACTAGAACTTTCCTGGAAGATTAAAAAAGAGGGAAGGAACTCGGCAAAATTAACTCCGCCT

</sequence>

<sequence>

<taxon idref="Oreophryne_unicolor"/>

----------------------------------------------TACGTCAAACTTAAAACCCAAAGGACTTGACGGTGTCTCACCCACCTAGAGGAGCCTGTTCTATAATCGATTATCCCCGATACACCCCACCGCTTCTTGCCACTCAGCCTGTATACCTCCGTCGCAAGCTTACCATGTGAATGCACCAGTAAGCCAAAAGATCCCATACAAACGTCAGGTCAAGGTGCAGCCTACGAAGCGGGAGATAATGGGCTACAATCTCTACTAGAACAAACGGAAGGCTGCATGAAACACAGCCCC--AAGGCGGATTTAGCAGTAAAAAGAAAATAGAGAGTTCTTTTTAATTAGGTA-TGGGACGCGTACACACCGCCCGTCACCCTCTTCA-ACAACGTTTCTAACACCACAAAACCGATAGAAGAGGCAAGTCGTAACACGGTAAGCGTACTGGAAAGTGCGCTTGGTACAATATGTAGCTTAACAAAAGCACCTTGCTTACACCAAGAACATGCCTGTTAAACTCAGACCATCTTGAGCTAAAATCCCAGCCCCATCCCATGCAACAACCCAACCCAACAACACCAATAAAACATTTTATTGACCCAGTAAAGGCGATCAAAAAGTCCTTAGGAG-----------------------------------------------------------------------------------------------------------------------------------------------------------------------------------------------------------------------------------------------------TAGAGATAGCTGGTTACTCGGGAAAAGGATCTAGTCCAACTTTAAAACATAACCCTTAATATACCTAGATTTAAATTTTATTCAAATAAGGTACAGCCTATTTGAAACAGGGTACAACCCCCCAGGTCGGGTAATGTTTTCACATAACAAAGTAGGCCTAAGAGCAGCCACCTTCCAAAAAGCGTCATAGCTTAACTATTTCCACCAATCCCAACCCCCGCTCAAACCCCCCCCCACCGAGTGACTCCATACTAATATGGATAACCCAATGTTAGAACTAGTAACAGGAACCATTCTCCCAAAGTAAGTGCAAGCCGGATTGAACTCACCGGCACCTACCGTCAATGAACCCCTAGTAATAACCACAAGAAAACCTTACTTACCCTGACGTTAACCTAACACCAGAACTTTTGAGGAAGATTTAAAAGGCAGGAAGGAACTCGGCAAAATAAACCCCGCCT

</sequence>

<sequence>

<taxon idref="Genyophryne_thomsoni"/>

------------------------------------GCCCGGGAATTACGCCAAGCTTAAAACCCAAAGGACTTGACGGTGTCCCACCCACCTAGAGGAGCCTGTTCTATAATCGATTATCCCCGATTTACCCCACCACTTCTCGATATTCAGTCTGTATACCTCCGTCGCAAGCTTACCATGTGAACGCACAAGTGAGCCAAAAGATTTCATCCATACGTCAGGTCAAGGTGCAACCTACGAAGCGGAAAGAAATGGGCTACAATCTCTATTAGAACAAACGAAAGATTACGTGAAATACACTCATCGAAGGCGGATTTAGTAGTAAAAAGAAACCAGAGAGTTCTTTTTAATTAGGCGCTGGGACGCGTACACACCGCCCGTCACCCTCTTCA-CACG--TTACTAACACCACATGTCTAGCAGAAGAGGCAAGTCGTAACACGGTAAGCGTACTGGAAAGTGCGCTTGGAACAAAATGTAACTTAACAAAAGTATCCTGCTTACACCAAGAATATGTTAGTAATATCCTAACCATTTTGAGCCTAAAATCTAGCCCTACTCAAATGATCTTTTTTCTCACTCAAA-----TAAAACATTCTCTCAACCTAGTAAAGGCGATTAAAAAGTTCTTAGGAG-----------------------------------------------------------------------------------------------------------------------------------------------------------------------------------------------------------------------------------------------------TAGAGATAGCTGGTTGTTCAGAAAACGGATATAGTCCAATTTTAAATTATAATAATAAATACATCCAGCTTTAAAATTCATTCAAATAAGGTACAGCCTATTTGAAACAGGATACAACCTAAAATATAGGGTAATGTCTTCACGTGATTAAGTCGGCTTAAAAGCAGCCACCTTCAAAAGAGCGTCACAGCTTTATCACCAATACTAATATCAATCTAAATACAAAACCTCTCCTACTGAATGACTTCATACATGTATGAAAAACCTCATGTTAAAACTAGTAACAAGAAAAATTTTCTATAAGCAAGTGTAAGCCGGAACGAATTCGCCGGCTCTTACCGTCAATGATCATACAGTAGTAACTACAAGAAAATTCTACTACCAACAACGTTAACCTTACACCAGAGCTTTTCAGGAAGATTAAAAAAGAGGGAAGGAACTCGGCAAAAGTAACCTCGCCT

</sequence>

<sequence>

<taxon idref="Cophixalus_humicola"/>

------------------------------------GCCCGGGAATTACGCCAAGCTTAAAACCCAAAGGACTTGACGGTGCCCCATCCTACTAGAGGAGCCTGTTCTATAACCGATTCTCCACGATACACCTCACCGCTTCTTG--ACACAGCCTGTATACCTCCGTCGCAAGCTTACCCTGTGAACGCACTAGTGAGCCTAAGGACCCTGTCAACACGTCAGGTCAAGGTGCAGCCTACGAAGCGGGAAGAAATGGGCTACAATCTCTACTAGAACAAACGAAAAACCACCTGCAACAGGGGTTTTGAAGGCGGATTTAGTAGTAAAAAGAAAGCAGAGAGTTCTTTTTAACCAGGCGCTGGGACGCGTACACACCGCCCGTCACCCTCTTCAAACTG--TTACTCACACCACACTACCAACAGAAGAGGCAAGTCGTAACACGGTAAGCGTACTGGAAAGTGCGCTTGGAACAAAGTGTAACTTAATAAAAGTCCCTTGCTTACACCAAGAATATGTCTGTAAAACTCCGACCACTCTGAGCCCAAAACCAAGCCCTACTTAATTAACCGCCTCCCCCA--------AAATAAACCATTCTACCACCCA-GTAAAGGCGATTAAAAAGTATCCAGGAG-----------------------------------------------------------------------------------------------------------------------------------------------------------------------------------------------------------------------------------------------------TAGAGATAGCTGGTTATTCAAGAAAAGGATGAAGTCCAACTTTAAAACATAAAACCAAATATTCCCAGATTTAAGCTTCATTCAAATAAGGCACAGCCTATTTGAAACAGGGTACAACCTATTACACAGGGTAATGGTTTCACGTGACTAAGTAGGCCTAAGAACAGCCACCTTTTAAAAAGCGTTACAGCTTCCTCACTACCACCAATCCCAACCAACACTCAAACCCCTTAATATTGAATGACTCCATGCCCCCATGGAAACCCCCATGTTAGAACTAGTAACAAGAATCATTCTCCATAAGCAAGTGTAAGCCGAACCGAACTCGCCGGCACTTATCGCCCATGAACTAACAGTAGTAACAACAAGAAAAGCCTACTTGTCCCAGCGTTAATCTTACACTAGAGTCTTTCAGGAAGATTAAAAAAAGAGGAAGGAACTCGGCAAAATTAACCCCGCCT

</sequence>

<sequence>

<taxon idref="Oreophryne_clamata"/>

----------------------------------------GGGAATTACGCCAAGCTT-AAACCCAAAGGACTTGACGGTGTCCCACCCCCCTAGAGGAGCCTGTTCTATAATCGATTCTCCCCGATCTACCTTACCGCCTCTTGCAATCCAGCCTGTATACCTCCGTCGTAAGCTTACCATGTGAACGCACCAGTGGGCCCAATGATCCCATACACACGTCAGGTCAAGGTGCAGCCTACGAAGCGGAAAGAAATGGGCTACAATTTCTATTAGAACAAACGGAAGACTACCTGAAACATAGTCATAGAAGGCGGATTTAGTAGTAAAAGGAAAATAGAGTGTTCCTTTTAAATAGGCACTGGGACGCGTACACACCGCCCGTCACCCTCTTCAAAGATTGTTACTGACACAACACGAACAACAGAAGAGGCAAGTCGTAACACGGTAAGTGTACTGGAAAGTGCACTTGGAACAAAATGTAACTTAATAAAAGTATCCTGCTTACACCAAGAACATGTCCGTAAAACTCGAACCATTTTGAGCTTAAAACCCAGCCCCTCCCCATGAAACAACTAACCCACAGAAAACAATAAAAACATTTT-TTAGCTTAGTAAAGGCGATTAAAAAGTACACAGGAG-----------------------------------------------------------------------------------------------------------------------------------------------------------------------------------------------------------------------------------------------------TAGAGATAGCTGGTTACTTAGGAAAAGGATGAAGTCCAACTTTAAAACACTACCATCAATAGCCCTAGATTTAAATTTCATTCAAATAAGGTACAGCCTATTTGAAACAGGATACAACCTACAATACAGGGTAATGCCCCCTTATGACCAAGTGGGCCTAAGAGCAGCCACCTTTCAAAGAGCGTTACAGCCTCATCACTTTCTCCAATCCCGATTCAAATCCAAAACCCTCGATACTGAGTGACTCCATATTAATATAGAAACCCTTATGTTAAAACTAGTAACAAGAATAATTCTACAAACGTAAATGTAAGCCGGACTGAACTCACCGGCACTTACCGCCGATGAGTCAACAGTAGCAACCACAAGAAAACCCCACTCATCCCAGCGTTAACCTTACACTAGAACTCTCCAGGAAGATTAAAAAAAGAGGAAGGAATTCAGCAAAATTAACCCCGCCT

</sequence>

<sequence>

<taxon idref="A_exclamitans"/>

CTATGCCTGACCGTAAAACA---ACTCACACACACCGCCAGGGGATTACGCCAAGCTTAAAACCCAAAGGACTTGACGGTGCCCCATCCCACTAGAGGAGCCTGTTCTATAATCGATTTTCCCCGATCCACCTCACCCCCTCTTGAACACCAGCCTGTATACCTCCGTCGCAAGCTTACCATGTGAACGCATTAGTGAGCTAAAAAATTTCATTCATACGTCAGGTCAAGGTGCAGCCCACGAGGCGGGAAGAAATGGGCTACAGTCTCTGCTAGAACAAACAGAAGGCTACATGAAACATAGCC--TGAAGGCGGATTTAGTAGTAAAAAGAAATTAGAGAGTTCTTTTTAACCCGGCCCTGGGACGCGTACACACCGCCCGTCACCCTCTTCA-----CGTTACTAACACCCTATGCACAATAGAAGAGGCAAGTCGTAACATGGTAAGCGTACTGGAAAGTGTGCTTGGAACAAAGTGTAACTTAATAAAAGTATCTTGCTTACACCAAGAATATGTCTGTGAAACTTAGACCGCTTTGAGCTAACAACCTAGCCCCTCCTCAAATGATATCCACCTTTCTCCCGTATAATAAAACATTTTTATTGCCAAGTAAAGGCGATTGAAAAGCACCTAAGAGCCTTAGCATTAGTACCGCAAGGGAAAAGTGAAATAGAAATGAAATAAATTTAAAGCCCAATAAAGTAAAGAATCCTTGTAGTACCTTTTGCATCATGGTCTAACTAGTCCCCCTAAGCAAAATGAACTTTAAGTTTAACACCCCGAAACTAGACGAGCTACTTCAAAACAGCCTTATGGGCCAACCCATCTCTGTTGCAAAAGAGTGGGAAGATTTTCAAGTAGAGGTGATATACCTATCGTGTCTAGAGATAGCTGGTTATTCAGGAAAAGGATATAGTCCAATTTTAAAACATAACATTAAATACTACTAGGTTTAATATTTATTCAAATAAGGTACAGCCTATTTGAAATAGGGCACAGCCTAAAATATAGGGTAATGTTTTTATGTGACTAAGTAGGCCTAAGAGCAGCCACCTTCTCAAAAGCGTTACAGCTTCACCACCATT-TTAATCCCAACTAAAACCCAAAACCCCCCCTACTGAATGACTCTATACTTATATAGAAAACCCTATGTTAGAACTAGTAATAAGAAATATTTTTCTAAAGCAAGTGTAAGCCGGAATGAATTCACCGGCACTTACCGCTAATGAATCCACAGTAATAACTACAAGAAAATCCTACTTAATCTCGCGTTAACCTTACACTAGACTTTTACCAGAAGATTAAAAAAGTAGGAAGGAACTCGGCAAAAGTAACCCCGCCT

</sequence>

<sequence>

<taxon idref="C_sp_nov1"/>

CTATGCCTGACCGTAAAACA---ACTCACACACACCGCCAGGGAATTACGCCAAGCTTAAAACCCAAAGGACTTGACGGTGTCCCATCCCACTAGAGGAGCCTGTTCTATAATCGATTCTCCCCGATCCACCTCACCGCCTCTTGAACACCAGCCTGTATACCTCCGTCGTAAGCTTACCATGTGAACGCATTAGTGAGCTAAAAAATTTCATTCATACGTCAGGTCAAGGTGCAGCCCACGATGCGGGAAGAAATGGGCTACAGTTTCTGCTAGAACAAACGGAAGGCTACATGAAACATAACC--TAAAGGCGGATTTAGTAGTAAAAAGAAAATAGAGAGTTCTTTTTAACCCGGCCCTGGGACGCGTACACACCGCCCGTCACCCTCTTCA-----CGTTACTAACACCCTATCCACAATAGAAGAGGCAAGTCGTAACATGGTAAGCGTACTGGAAAGTGTGCTTGGAACAAAATGTAACTTAATAAAAGTATCTTGCTTACACCAAGAACATGTCTGTTAAACTCAGACCACTTTGAGCTAACAACCTAGCCCC-TCAAATGAAACTTTTCTC------TAACATAATAAAACATTTTTATTGCTAAGTAAAGGCGATCGAAAAGCACCTAAGAGCTTTAGCATTAGTACCGCAAGGGAAAGGTGAAATAAAAATGAAATAAATTTAAAGCCCAATAAAGTAAAGAATCCTTGTAGTACCTTTTGCATCATGGTTTAACTAGTCCTCTTAAGCAAAATGAACTTTAAGTTTAACACCCCGAAACTAGACGAGCTACTTCAAAACAGCCTCATGGGCCAACCCGTCTCTGTTGCAAAAGAGTGGGAAGATTTTCAAGTAGAGGTGATATACCTACCGTGTCTAGAGATAGCTGGTTACTCAAGAAAAGGATATAGTCCTATTTTAAAACATAACATTAAATACTACTAGGTTTAATATTCATTCAAATAAGGTACAGCCTATTTGAAACAGGGCACAGCCTAAAATATAGGGTAATGTTTTCATGTGACTAAGTAGGCCTAAGAGCAGCCACCTTCTCAAAAGCGTTACAGCTTCACCACTATT-TTAATCCCAACCAAAACCCAAAACCCCCCCTACTGAGTGACTCTATACTTATATAGAAAACCCCATGTTAGAACTAGTAATAAGAAACATTCTTCTAAAGCAAGTGTAAGCCGGAATGAATTCACCGACACTTACCGCTAATGAACCCACAGTAATAACTACAAGAAAATCCTACTTAATTTCGCGTTAACCTTACACTAGACTTTTACCAGAAGATTAAAAAAACAGGAAGGAACTCGGCAAAAGTAACCCCGC--

</sequence>

<sequence>

<taxon idref="Cophixalus_tridactylus"/>

------------------------------------GGCCTGGAATTACGCCAAGCTT-AAACCCAAAGGACTTGACGGTGCCCCATCCTACTAGAGGAGCCTGTTCTATAATCGATTCCCCCCGATATACCTCACCGCCTCTTGCA-AACAGCCTGTATACCTCCGTCGCAAGCTTACCATGTGAACGCACTAGTGAGCTCAAAGGTCCCACCCACACGTCAGGTCAAGGTGCAGCCTACGGGACGGCAAGAAATGGGCTACAATCTCTACTAGAACAAACGAAAGACTACATGCAACACAGTCATTGAAGGCGGATTTAGCAGTAAAAAGGAAACAGAGAGTTCTTTTTAACCAGGCGCTGAGGCGCGTACACACCGCCCGTCACCCTCTTCAAACTG--TTACTAACACAATATAATACACAGAAGAGGCAAGTCGTAACACGGTAAGCGTACTGGAAAGTGTGCTTGGAACAAAGTGTAACTTAACGAAAGTCCCTTGCTTACACCAAGAACATGTCCGTGAAA-CCCGACCACTCTGAGCCCTAAACCCAGCCCTACTTAATGGA-CCCTCCCCACCCCCCCCCCCAGCAAATCATT-TGATAACCCAGTAGAGGCGATTAAAAAGTGACTAGGCG-----------------------------------------------------------------------------------------------------------------------------------------------------------------------------------------------------------------------------------------------------TAGAGATAGCTGGTTATTCAAGAAAAGGATCAAGTCCAACTTTAAGGCATAAAATCAAATACACTCAGACTTAAATTTCATTCAAATAAGGTACAGCCTATTTGAAATAGGGTACAACCTATCACACAGGGTAATGCCTCCACGTGACTAAGTAGGCCTAAGAGCAACCACCTTCTAAAAAGCGTCACAGCTTTCTCACTATTACCAATCCCAACCAACACTCAAACCCCTCCCCATTGAATGACTCCATGCCCCCATGGAAAACCCCATGCTAGAACTAGTAACAAGAATAATTCTCTACAAGTAAGTGTAAACCGGACTGAACTCACCGGCACCTACCGCCTATGAGCCAACAGTAGCAACCCCAAGAAAAGCCTACTTATCCCAGCGTTAATCTTACACTAGAACCTTTCAGGAAGATTAAAAAATGCGGAAGGAACTCGGCAAAAACAACCCCGCCT

</sequence>

<sequence>

<taxon idref="Copiula_major"/>

------------------------------------GCCAGGGGATTACGCCAAGCTTAAAACCCAAAGGACTTGACGGTGCCCCATCCACCTAGAGGAGCCTGTTCTATAATCGATTCTCCCCGATCTACCTCACCCCTTCTTGAACCCCAGTCTGTATACCTCCGTCGCAAGCTTACCATATGAACGTCCTAGTAAGCCTAAAGGTTTTGCCCCCACGTCAGGTCAAGGTGCAGCCCACGAAGCGGAAAGAAATGGGCTACAATCTCTACTAGAACAAACGGAAGACTTCATGAAACACAGTCA-TTAAGGCGGATCTAGTAGTAAAAAGAAAATAGAGAGTTCTTTTTAATAAGGCGCTGGGACGCGTACACACCGCCCGTCACCCTCTTCATAATG--TTATTAACTCTACACATCCTACAGAAGAGGCAAGTCGTAACACGGTAAGCGTACTGGAAAGTGCGCTTGGAACAAAATGTAGCTTAACCAAAGCCTCTTGCTTACACCAAGAACACGTCTGTGAAATTCTGACCATTTTGAGCTAAAAGCCTAGCCCTACCTTTAATACCACTTTTCCACATATAAACAAACAAAACATTTTCTTAACCCAGTAGAGGCGATTAAAAAGTTTACAGGCG-----------------------------------------------------------------------------------------------------------------------------------------------------------------------------------------------------------------------------------------------------TAGAGATAGCTGGTTATTCAGGAAAAGGATATAGTCCAACTTTAAAATGTACCATCAAATATGACTAGGTTTAAAATTTATTCAAATAAGGTTCAGCCTATTTGAAACAGGATACAACCTACCACATAGGGTAATGTTTATACATGACCAAGTAGGCCTAAGAGCAGCCATCTTTTAAAAAGCGTTATAGCCTCATCATCACAATTAATTCCAACCTTAACCCAAAACCCCCCATACTGAATGACTCCATATTTATATGGAAAACCTTATGTTAGAACTAGTAACAAGAACACTTCTACCAAAGTATGTGTAAGCCGGACTGAAATCACCGGCATCTATCGCCAATGAACCTGCAGTAGCAACCACAAGAAAACTCTACTCTTCAAAGCGTTAACCTTACACTAGAGCTTTTCTGGAAGATTAAAAAAAGAGGAAGGAACTCGGCAAAATGAGCCCCGCCT

</sequence>

<sequence>

<taxon idref="Oxydactyla_crassa"/>

------------------------------------GCCAGGGAATTACGCCAAGCTTAAAACCCAAAGGACTTGACGGTGTCCCACCCTCCTAGAGGAGCCTGTTCTCTAATCGATTCTCCCCGATCCACCTCACCGCCTCTTGAAACCCAGTCTGTATACCTCCGTCGCAAGCTTACCATGTGAACGCATTAGTGGGCTTAAAAATCCCATTCACACGTCAGGTCAAGGTGCAGCCCACGAGGCGGGAAGAAATGGGCTACAATCTCTATTAGAACAAACGAAAGACTGCATGCAATACAGTTAC-CAAGGCGGATTTAGAAGTAAAAAGAAAATAGAGAGTTCTTTTTAATTAGGCCCTGGGACGCGTACACACCGCCCGTCACCCTCTTCACAATG--TCACTAACACTAAAAAATTCACAGAAGAGGCAAGTCGTAACACGGTAAGCGTACTGGAAAGTGTGCTTGGAACAAAATGTAGCTTAATAAAAGCCCCTTGCTTACACCAAGGATATGTCTGTTAAACTCTGACCCTTTTGAGCCCAAAATCTAGCCCTATTCTAATGAACAATTTAATTAAACTAAACAAATAAAACATTTTTTTAACCAAGTAAAGGCGATTAAAAAGTACCTAGGAG-----------------------------------------------------------------------------------------------------------------------------------------------------------------------------------------------------------------------------------------------------TAGAGATAGCTGGTTATTCAGGAAAAGGATATAGTCCAATCTTAAATTAGAACCCTAAATACGCCAAGATTTAAAATTTATTCAAATAGGGTACAGCCTATTTGAAACAGGGTACAACCTATCACATTGGGTAATGTTTTAACCTGACCAAGTAGGCTTAAAAGCAGCCATCTTTAAAAAAGCGTTACAGCTTCATCATTGCTATCAATTTTAACCTAACCCCAAAACCCCTCATACTGAATGACTCCATATTAATATGGAAGACCTTATGTTAGAACTAGTAATCAGAATAATTCTCATAAAGTAAGTGTAAGCCGGAACGAACTCACCGGCACTTACCGCCAATGAATCCATAGTAACAACTACAAGAAAATATTACTTACTCTGACGTTAACCTTACACTAGAACTTTACTGGAAGATTAAAAAAGGAGGAAGGAACTCGGCAAAATTGATCCCGCCT

</sequence>

<sequence>

<taxon idref="C_foyas_2"/>

CTATGCTTAGCCATAAAACATTAACCCACACAACCCGCCAGGGAATTACGCCAAGCTTAAAACCCAAAGGACTTGACGGTGCCCCACCCACCTAGAGGAGCCTGTTCTATAATCGATTCTCCCCGATCTACCTCACCGCCTCTTGAAAACCAGTCTGTATACCTCCGTCGCAAGCTTACCATGTGAACGCACCAGTGAGCCAAAAGATACCACCCATACGTCAGGTCAAGGTGCAGCCCACGAAGCGGGAAGAAATGGGCTACAATCTCTACTAGAACAAACGGAAGACTGTATGAAACACAGTTA-TGAAGGCGGATTTAGTAGTAAAAAGAAAACAGAGTGTTCTTTTTAACTAGGCGCTGGGACGCGTACACACCGCCCGTCACCCTCTTCAAAAAG--TCACTAACACTATACTACTAATAGAAGAGGCAAGTCGTAACACGGTAAGTGTACTGGAAAGTGCACTTGGAACAAAATGTAACTTAACTAAAGTATCTTGCTTACACCAAGACTATGTCAGTGAAACTCTAACCACTTTGAGCCTAAATCTCAGCCCTATTCCACATGTTCCTTTTTCCTAAC------AACAAAACATTTTTTCAACTAAGTAAAGGCGATTAAAAAGTTACTAGGAGCCCCAACACCAGTACCGCAAGGGAAAAGTGAAATAAAAATGAAATAAATCTAAAGCACAACGCAGTAAAGACCCCTTGTAGTACCTTTTGCATCATGGTCTAACTAGTTTAACCAAGCAAAACGAATTTTTAGTTTGACCCCCCGAAACTAAGCGAGCTACTTCAAAACAGCCCCGCGGGCCAACCCATCTCTGTTGCAAAAGAGTGGGAAGATTTTCAAGTAGAGGTGACACACCTACCGAGCCTAGAGATAGCTGGTTATTCAGGAAAAGGATCTAGTCCAATTTTAAAACTTACCCTAAAGTATGCCCAGGTTTAAAGTTTATTCAAATAAGGTACAGCCTATTTGAAACAGGATACAACCTATGATGCAGGGTAATGTTAACATGTGACAAAGTAGGCCTAAAAGCAGCCACCTTACAAAAAGCGTTATAGCTTCATCACCTACATCAATTCCAATCAAAATCCAAAGCCCTCCGTACTGAATGACCCCATATTTATATGGGAGACCCTATGTTAGAACTAGTAACAAGAATAATTCTCAATAAGCACGTGTAAGCCGGAATGAATTCACCGGCACTTACCGTCAATGAATTAATAGTAGCAACTACAAGAAAACCCTACTTACCCAAACGTCAACCTTACACTAGAGCTTTTCAGGAAGATTAAAAAAAGAGGAAGGAACTCGGCAAAAGTAACCCCGCCT

</sequence>

<sequence>

<taxon idref="C_laurini"/>

------------------------------------GCCAGGGAATTACGCCAAGCTTAAAACCCAAAGGACTTGACGGTGCCCCACCCTCCTAGAGGAGCCTGTTCTATAATCGATTCTCCCCGATCTACCTCTCCGCCTCTTGAAAACCAGTCTGTATACCTCCGTCGCAAGCTTACCATGTGAACGCA-CAGTGAGCCAAAAGATCCCACCCAAACGTCAGGTCAAGGTGCAGCCCACGAAGCGGGAAGAAATGGGCTACAATCTCTACTAGAACAAACGGAAAACTGTATGAAACACAGTTA-TGAAGGCGGATTTAGTAGTAAAAAGAAAACAGAGTGTTCTTTTTAACTAGGCGCTGGGACGCGTACACACCGCCCGTCACCCTCTTCAAAAAG--TTACTGACACAATACCACTAACAGAAGAGGCAAGTCGTAACACGGTAAGTGTACTGGAAAGTGCACTTGGAACAAAATGTAACTTAATCAAAGTATCCTGCTTACACCAAGATTATGTCCGTGAAATTCTGACCATTTTGAGCCTAAATCTCAGCCCTACCCTATGAAAATTTTTTCCCCCCC-------CCAAAACATTTTTTCAACCCAGTATAGGCGATTAAAAAGTTACTAGGAG-----------------------------------------------------------------------------------------------------------------------------------------------------------------------------------------------------------------------------------------------------TAGAGATAGCTGGTTATTCAGGAAAAGGATTTAGTCCAATTTTAAAACATA-CCAAAAATATACCTAGCTTTAAAATTTATTCAAATAAGGTACAGCCTATTTGAAACAGGATACAACCTATAATGTCGGGTAATGTTAACATATGACAAAGTAGGCCTAAAAGCAGCCACCTTATAAAAAGCGTTATAGCTTAATCATCCTTATTAATCCTAACCAAAACCCAAAACCCTCCCTACTGAATGACCCCATATTTATATGGAAGCCAATATGTTAGAACTAGTAACAAGAATAATTCTCCAACAGCACGTGTAAGCCGGAATGAATTCACCGGCATTTACCGCCAATGAATCAATAGTAGCAACTACAAGAAAACCCTACCTGCCTAAACGTTAACCTTACACTAGAGCTTTTCAGGAAGATTAAAAAAGGGGGAAGGAACTCGGCAAAAGTAACTCCGCCT

</sequence>

<sequence>

<taxon idref="C_tuberculus"/>

-TATGCTTAGCTATAAAATATTAACCCACACAACCCGCCAGGGAATTACGCCAAGCTTAAAACCCAAAGGACTTGACGGTGCCCCACCCCTCTAGAGGAGCCTGTTCTATAATCGATTATCCACGATCTACCTCACCGCTTCTCGAAACCCAGTCTGTATACCTCCGTCGTAAGCTTACCATGTGAACGCATCAGTGAGCCTAATGATTTCATCAACACGTCAGGTCAAGGTGCAGCCCATGGAGCGGAAAGAAATGGGCTACAATCTCTATTAGAACAAACGAAAAACTGTGTGAAACACAGTTACTGAAGGCGGATTTAGTAGTAAAAAGAAAATAGAGAGTTCTTTTTAACTAGGCGCTGGGGCGCGTACACACCGCCCGTCACCCTCTTCA-AAAACCTACTTAACATTGCACAAT-AATAGAAGAGGCAAGTCGTAACATGGTAAGTGTACTGGAAAGTGCACTTGGAACAAAATGTAGCTTAATAAAAGTGTCCTGCTTACACCTAGATTATGTCCGTGAAACCCCGACCATTTTGAGCCTAAACCACAGCCCTACCCCATGAGACTTTACCAACA---AAATAAAATAAAACATTCTTTTAACCAAGTAAAGGCGATTAAAAAGTCATTAGGAGCCCCAGAGCCAGTACCGCAAGGGAAAAATGAAAT-AAAATGAAATAAACTAAAAGCACTACAAAGTAGAGACCCCTCGTAGTACCTTTTGCATCATGGTTTAACTAGTTTAACCAAGCAAAACGAACTTTAAGTTTGACCCCCCGAAACTAAGCGAGCTACTTCAGAACAGCCTCACGGGCCAACCCATCTCTGTTGCAAAAGAGCGGGAAGATTCTTAAGTAGAGGTGACACGCCTACCGAGCTTAGAGATAGCTGGTTATTCAAAAAAAGGATATAGTCCAACTTTAAAATATAACCCAAAATATACACAAGTTTAAATTTTATTCAAATAGGGTACAGCCTACTTGAAACAGGATACAACCTATAATGCAGGGTAATGTTTCCATGTAATCAAGTAGGCCTAAAAGCAGCCATCTTACAAAAAGCGTTACAGCTTCACTACCATCACTAATTTTAATCAAAACCCAAAACCCTCCATATTGAATGACTCCATATTTGTATGGAAAACCCTATGTTAAAACTAGTAACAAGAATAATTCTCACAAAGCACGTGTAAGCCGAAATGAACCCACCGGCACTTACCGCCAATGAATAAACAATAGCAACCACAAGAAAATCCTACTCCCCCCAGCGTCAACCTTACACTAGAGCTTCCCTGGAAGATTAAAAAAAGAGGAAGGAACTCGGCAAAAGCAACCCC----

</sequence>

<sequence>

<taxon idref="C_sp4"/>

CTATGCTTAGCCGTAAAATATTAACCCACACAACCCGCCAGGGAATTACACCAAGCTTAAAACCCAAAGGACTTGACGGTGCCCCATCCCTCTAGAGGAGCCTGTTCTATAATCGATTTTCCCCGATCCACCTCACCGTTCCTTGAAATCCAGTCTGTATACCTCCGTCGTAAGCTTACCATGTGAACGCACCAGTGAGCCTAAAGATTTCATCAACACGTCAGGTCAAGGTGCAGCCCACGGAACGGGAAGAAATGGGCTACAATCTCTACTAGAACAAACGGAAAACTGCGTGAAACACAGTTACTGAAGGCGGATTTAGTAGTAAAAAGAAAATAGAGAGTTCTTTTTAATCAGGCCCTGGGACGCGTACACACCGCCCGTCACCCTCTTCA-CAAG--TCTCTAACACCATACAATTGATAGAAGAGGCAAGTCGTAACATGGTAAGTGTACTGGAAAGTGCACTTGGAACAAAATGTAGCTTAACAAAAGTATCCTGCTTACACCTAGACCATGTCCGTGAAACCCTGACCATTTTGAGCCTAAATCTTAGCCCTATCTTATGAAACCCCCTCCCCCTCCAA-------AAAACATTCTTTTAACCAAGTAAAGGCGATTAAAAAGTTATTAGGAGCCCCAGAACCAGTACCGCAAGGGAAAATTGAAATAAAAATGAAATAAATTTAAAGCACCACAAAGTAGAGACCCCTCGTAGTACCTTTTGCATCATGGTTTGACTAGTTTAATCAAGCAAAACGAACTTTAAGTTTGACCCCCCGAAACTAAGCGAGCTACTTCAGAACAGTCTTATGGACTAACCCGTCTCTGTTGCAAAAGAGTGGGGAGATTCTCAAGTAGAGGTGATACACCTACCGAGCTTAGAGATAGCTGGTTATTCAAAAAAAGGATACAGTCCAACTTTAAAACCTAACCCAAAATATACTAAGGTTTAAAATTTATTCAAATAAGGTACAGCCTATTTGAAACAGGATACAACCTATAATGCAGGGTAATGCTTTCATGTAGCCAAGTAGGCCTAAGAGCAGCCACCTTATGAAAAGCGTTACAGCTTCACTACTATTATTAATTTTAACTAAAACCCAAAACCCTCCGTATTGAATGACTCCATGTTTATATGGAAGACCCTATGTTAAAACTAGTAACAAGAATAATTCTCCCAAAGCACGTGTAAGCCGGAATGGACCCACCGGCATTTATCGCTGATGAATAAATAGTAATAACTACAAGAAAATCCTACTCATCCAAGCGTCAACCTTACACCAGAGCTTTTCTGGAAGATTAAAAAAAGGGGAAGGAACTCGGCAAAAGTAACCCCGCC-

</sequence>

<sequence>

<taxon idref="C_sp_juha"/>

CTATGCTTAGCCGTAAAATATTAACCCACACAACCCGCCAGGGAATTACGCCAAGCTTAAAACCCAAAGGACTTGACGGTGTCCCATCCCCCTAGAGGAGCCTGTTCTATAATCGATTCTCCCCGATCTACCTTACCGTTTCTTGAAACCCAGTCTGTATACCTCCGTCGCAAGCTTACCATGTGAACGCA-CAGTGAGCCTAAAGATCTCATCCAAACGTCAGGTCAAGGTGCAGCCCACGGAACGGAAAGAAATGGGCTACAATCTCTACTAGAACAAACGGAAAGCTGTATGAAACACAGTTATTGAAGGCGGATTTAGTAGTAAAAAGAAAACAGAGTGTTCTTTTTAACTAGGCGCTGGGACGCGTACACACCGCCCGTCACCCTCTTCA-AAAG--TCATTAACACCATGCAATTAATAGAAGAGGCAAGTCGTAACATGGTAAGTGTACTGGAAAGTGCACTTGGAACAAAATGTAGCTTAATGAAAGCATCCTGTTTACACCAAGACCATGTCTGTGAAACCCTGACCATTTTGAGCCTAAATCTCAGCCCTACCCCATGAACCTTTCTTTTCCCCCCCCCATAATAAAACATTTTCTTAACCCAGTAAAGGCGATTAAAAAGTTACTAGGAGCTTCAACATCAGTACCGCAAGGGAAAATTGAAATAAAAATGAAATAAATTTAAAGCATTTCAAAGTAAAGACCCCTT------CCTTTTGCATCATGGTCTAACTAGTTTAACCAAGCAAAACGAACTTTAAGTTTGACCCCCCGAAACTAAGCGAGCTACTTCAAAACAGCCTTATGGGCCAACCCGTCTCTGTTGCAAAAGAGTGGGAAGATTTTCAAGTAGGGGTGACACACCTACCGAGCCTAGAGATAGCTGGTTATTCGGGAAAAGGATCTAGTCCAATTTTAAAACACAACCCACAATACACCAAGCTTTAAAATTAATTCAAATAAGGTACAGCCTATTTGAAACAGGATACAACCTATAACGCAGGGTAATGTTTTTACATGACAAAGTAGGCCTAAGAGCAGCCACCTTATAAAAAGCGTTACAGCTTCACCATCATCACCAATCCCAACCAAAACCCAAAACCCTCAATACTGAATGACCCCATACTTATATGGAAAACCTTATGTTAGAACTAGTAACAAGAATAATTCTCCAAAAGCACGTGTAAGCCGGAATGAATCCGCCGGCATTTATCGCCAATGAATAAATAGTAGCAACCACAAGAGAACCCTACTTACCCAAGCGTTAACCTCACACTAGAACTTTCCAGGAAGATTAAAAAAAGGGGAAGGAACTCGGCAAAAGTAACCCCGCCT

</sequence>

<sequence>

<taxon idref="C_sp3"/>

CTATGCTTAGCCGTAAAATATTAACCCACACAGCCCGCCAGGGAATTACACCAAGCTTAAAACCCAAAGGACTTGACGGTGTCCCACCCTTCTAGAGGAGCCTGTTCTATAATCGATTCTCCCCGATCTACCTCACCGTCTCTTGAAATCCAGTCTGTATACCTCCGTCGCAAGCTTACCATGTGAACGCACCAGTGAGCCTAAAGATTCCACCCACACGTCAGGTCAAGGTGCAGCCCACGGAACGGAAAGAAATGGGCTACAATCTCTACTAGAACAAACGGAAAACTGCATGAAACACAGTTACTGAAGGCGGATTTAGTAGTAAAAAGAAAATAGAGAGTTCTTTTTAACTAGGCACTGGGACGCGTACACACCGCCCGTCACCCTCTTCA-AAAG--TCACTAACACCATATAATTAATAGAAGAGGCAAGTCGTAACATGGTAAGTGTACTGGAAAGTGCACTTGGAACAAAATGTAACTTAATAAAAGTACCCTGCTTACACCAAGTCTATGTCTGTGAAACCCCGACCATTTTGAGCCTAAATCTCAGCCCTACCCAATGAATTCTCCAACCCACCC-------CTAAAACATTCTTTCAACCAAGTAAAGGCGATTAAAAAGTTACTAGGAGCCCCAACACCAGTACCGCAAGGGAAAGTTGAAATAAAAATGAAATAAATTTAAAGCACTACAAAGTAAAGACCACTTGTAGTACCTTTTGCATCATGGTTTAACTAGTTTAACCAAGCAAAACGAACTTTAAGTTTGACCCCCCGAAACTAAGCGAGCTACTTCAAAACAGCCTTATGGGCGAACCCATCTCTGTTGCAAAAGAGTGGGAAGATTTTCAAGTAGAGGTGACACACCTACCGAGCCTAGAGATAGCTGGTTATTTAGGAAAAGGATATAGTCCAATTTTAAAACACAACTTCAAATATACCTAGCTTTAAAATTTATTCAAATAAGGTACAGCCTATTTGAAACAGGATACAACCTATAACGCAGGGTAATGTTTTTACGTGACAAAGTAGGCCTAAGAGCAGCCACCTTATAAAAAGCGTTATAGCTTCATCACCATCATCAATCCCAATCAAGATCCAAAACCCTCTATACTGAATGACTCCATATTTATATGGAAGACCTTATGTTAGAACTAGTAACAAGAATAATTCTCTCAAAGCACGTGTAAGCCGGAATGAATTCACCGGCATTTACCGCCAATGAATAAATAGTAGTAACCACAAGAAAATTCTACTTACCTAAGCGTTAATCTAACACTAGAACTTTACAGGAAGATTAAAAAAAGAGGAAGGAACTCGGCAAAATTAACTCCGCCT

</sequence>

<sequence>

<taxon idref="C_darlingtoni"/>

CTATGCTTAGCCGTAAAATATTAACCCACACAGCCCGCCAGGGAATTACGCCAAGCTTAAAACCCAAAGGACTTGACGGTGTCCCATCCCTCTAGAGGAGCCTGTTCTATAATCGATTCTCCCCGATCTACCTTGCCGTCCCTTGAAACCCAGTCTGTATACCTCCGTCGCAAGCTTACCATGTGAACGCA-CAGTGAGCCCAAAGATCCCACCCATACGTCAGGTCAAGGTGCAGCCCACGGAACGGAAAGAAATGGGCTACAATCTCTACTAGAACAAACGGAAAACTGCATGAAATACAGTTATTGAAGGCGGATTTAGTAGTAAGAAGAAAATAGAGAGTTCTTTTTAACAAGGCGCTGGGACGCGTACACACCGCCCGTCACCCTCTTCAAAAAG--TCACTAACACCCAACAATTAATAGAAGAGGCAAGTCGTAACATGGTAAGTGTACTGGAAAGTGCACTTGGAACAAAATGTAACTTAATAAAAGTATCCTGCTTACACCAAGACTATATCTGTGAAACCCCGATCATTTTGAGCCTAAATTCCAGCCCTACCCCGCATGCCCCCCTCCCCTCCC------ACCAAAACATTCTTTCAACCAAGTAAAGGTGATTAAAAAGTTATTAGGAGCTCCAACACCAGTACCGCAAGGGAAAAATGAAATAAAAATGAAACAAATTTAAAGCACTACAAAGTAGAGATCCCTTGTAGTACCTTTTGCATCATGGTCTAACTAGTTTACCCAAGCAAAACGAATTTTAAGTTTGCCCCCCCGAAACTAAGCGAGCTACTTCAAAACAGCCTTATGGGCCAACCCATCTCTGTTGCAAAAGAGTGGGAAGATTTTCAAGTAGAGGTGACACACCTATCGAGCCTAGAGATAGCTGGTTATTCAAGAAAAGGATATAGTCCAATTTTAAAACACAACCTAAAATATACCTAGTTTTAAAATTTATTCAAATAAGGTACAGCCTATTTGAAACAGGATACAACCTATTACGCAGGGTAATGTTTTTATATGACAAAGTAGGCCTAAGAGCAGCCACCTTATAAAAAGCGTTACAGCTTCACCATCATCACCAATCCCAACTAAAACTCAAAACCCTCAATATTGAATGACCCCATACTTATATGGAAAACCTTATGTTAGAACTAGTAACAAGAATAATTCTCCAAAAGCACGTGTAAGCCGGAATGAATTCACCGGCACTTACCGCTAATGAATAAATGGTAGCAACCACAAGAAAACCCTACTCACCCAAGCGTTAACCTCACACCAGAGCTTTCCCGGAAGATTAAAAGAAGGGGAAGGAACTCGGCAAAAGTAACCCCGCCT

</sequence>

<sequence>

<taxon idref="C_pandanicolus"/>

-------------------------CCACACAGCCCGCCAGGGAATTACGCCAAGCTTAAAACCCAAAGGACTTGACGGTGTCCCATCCCCCTAGAGGAGCCTGTTCTATAATCGATTCTCCCCGATCTACCTTGCCGTCCCTTGAAATCCAGTCTGTATACCTCCGTCGCAAGCTTACCATGTGAACGCA-TAGTGAGCCCAAAGATCCCACCCATACGTCAGGTCAAGGTGCAGCCCACGGAACGGAAAGAAATGGGCTACAATCTCTATTAGAACAAACGGAAAACTGTGTGAAATACAGTTATTGAAGGCGGATTTAGTAGTAAAAAGAAAATAGAGAGTTCTTTTTAACTAGGCGCTGGGACGCGTACACACCGCCCGTCACCCTCTTCAAAAAAAGTCACTAACACCATATAATCAATAGAAGAGGCAAGTCGTAACATGGTAAGTGTACTGGAAAGTGCACTTGGAACAAAATGTAACTTAATAAAAGTACCCTGCTTACACCAAGACTATGTCTGTGAAACCCCGACCATTTTGAGCCTAAATCCCAGCCCTACCCCGCATGCTTTTTTCCCCCTCC---CCAAATAAAACATTCTTTCAACTAAGTAAAGGCGATTAAAAAGTTGCTAGGAGCCCCAACACCAGTACCGCAAGGGAAAAGTGAAATAAAAATGAAATAAATT-----------------------------------TTTTGCATCATGGTCTAACTAGTTTAACCAAGCAAAACGAATTTTAAGTTTGCCTCCCCGAAACTAAGCGAGCTACTTCAAAACAGCCTTACGAGCCAACCCGTCTCTGTTGCAAAAGAGTGGGAAGATTTTCAAGTAGAGGTGACACACCTATCGAGCCTAGAGATAGCTGGTTATTCAAGAAAAGGATATAGTCCAACTTTAAAACACAACCTAAAATATACCCAGGTTTAAAATTTATTCAAATAAGGTACAGCCTATTTGAAACAGGATACAACCTATTACGCAGGGTAATGTTTTGATATGACAAAGTAGGCCTAAGAGCAGCCACCTTATAAAAAGCGTTACAGCTTCACCATTATCACCAATCCCAACTAAAACTCAAAACCCTCGATATTGAATGACCCCATACTTATATGGAAAACCCTATGTTAGAACTAGTAACAAGAATAATTCTCCAAAAGCACGTGTAAACCGGAATGAATTCCCCGGTATTTACCGCCAATGAATAAATAGTAGCAACCACAAGAAAACCCTACTACCCCCAGCGTTAACCTCACACCAGAGCTTTCCAGGAAGATTAAAAAAAGGGGAAGGAACTCGGCAAAAGTAACCCCGCCT

</sequence>

<sequence>

<taxon idref="C_fafniri"/>

CTATGCTTAGCCATAAAATATTAACCCACACAACCCGCCAGGGAATTACACCAAGCTTAAAACCCAAAGGACTTGACGGTGTCCCATCCCCCTAGAGGAGCCTGTTCTATAATCGATTCTCCCCGATCTACCTTGCCGTCTCTTGAAATCCAGTCTGTATACCTCCGTCGCAAGCTTACCATGTGAACGCA-TAGTGAGCCCAAAGATCCCACCCATACGTCAGGTCAAGGTGCAGCCCACGGAACGGAAAGAAATGGGCTACAATCTCTACTAGAACAAACGGAAAGCTGTATGAAATACAGTCATTGAAGGCGGATTTAGTAGTAAAAAGAAAATAGAGAGTTCTTTTTAACTAGGCACTGGGACGCGTACACACCGCCCGTCACCCTCTTCAAAAAG--TCACTAACACCCCACAATTAATAGAAGAGGCAAGTCGTAACATGGTAAGTGTACTGGAAAGTGCACTTGGAACAAAATGTAACTTAATAAAAGTATCCTGCTTACACCAAGACTATGTCTGTGAAACCCCGACCATTTTGAGCCTAAACCCCAGCCCTACCACATGAATTTCCCCTCCCCCCC---CCAAATAAAACATTCTCTCAACCAAGTAAAGGCGATTAAAAAGTTACTAGGAGCCCCAACACCAGTACCGCAAGGGAAAGGTGAAATAAAAATGAAATAAATTTAAAGCACTACAAAGTAGAGACCCCTTG-----CCTTTTGCATCATGGTCTAACTAGTTTAACCAAGCAAAACGAATTTTAAGTTTGCCCCCCCGAAACTAAGCGAGCTACTTCAAAACAGCCTTATGGGCCAACCCGTCTCTGTTGCAAAAGAGTGGGAAGATTTTCAAGTAGAGGTGACACACCTACCGAGCCTAGAGATAGCTGGTTATTCAAGAAAAGGATATAGTCCAATTTTAAAACGCAACCTGAAATATACCTAGCTTTAAAATTTATTCAAATAAGGTACAGCTTATTTGAAACAGGATACAACCTATTACGTAGGGTAATGTTTTCATATGACAAAGTAGGCCTAAGAGCAGCCACCTTATAAAAAGCGTTACAGCTTCACCATCATCACCAATCCCAACTAAAACCCAAAACCCCCAATATTGAATGACCCCATACCCATATGGAAAACCCTATGTTAGAACTAGTAACAAGAATAATTCTCCCAAAGCACGTGTAAACCGGAATGAATCCACCGGCATTTACCGCCAATGAATAAACAGTAGTAACCACAAGAAAATCCTACTAATCTAAGCGTTAGCCTCACACCAGAGCTTTCCCGGAAGATTAAAAAAAGGGGAAGGAACTCGGCAAAAGTAACCCCGCCT

</sequence>

<sequence>

<taxon idref="C_foyas_1"/>

CTATGCTTAGCCGTAAAATATTAACCCACACAGCCCGCCAGGGAATTACA-TAAGCTTAAAACCCAAAGGACTTGACGGTGTCCCATCCCTCTAGAGGAGCCTGTTCTATAATCGATTCTCCCCGATATACCTCACCGTTTCTTGTAATCCAGTCTGTATACCTCCGTCGCAAGCTTACCATGTGAACGTACAAGTGAGCCTAAAGATTCCACCCACACGTCAGGTCAAGGTGCAGCCTACGAAACGGAAAGAAATGGGCTACAATCTCTATTAGAACAAACGGAAAACTGTATGAAACACAGTCATTGAAGGCGGATTTAGTAGTAAAATGAAAATAGAGAATTCTTTTTAACTAGGCGCTGGGACGCGTACACACCGCCCGTCACCCTCTTCAAGAAG--TCACTAACACTATATAATCAATAGAAGAGGCAAGTCGTAACATGGTAAGTGTACTGGAAAGTGCACTTGGAACAAAATGTAGCTTAACAAAAGTACCCTGCTTACACCAAGACCATGTCTGCGAAACCCAGACCATTTTGAGCTTAAACCTCAGCCCTACCCCATGAACTCCCATCCTCCCCA--------TAAAACATTTTCCTGACTAAGTAAAGGTGATTAAAAAGTCTCTAGGAGCCCCAGCACTAGTACCGCAAGGGAAAAGTGAAATAAAAATGAAATAGATTTAAAGCGCTACAAAGTAAAGATCCCTTGTAGTACCTTTTGCATCATGGTCTGACTAGTTTAACCAAGCAAAATGAATTTTAAGTTTGTCCCCCCGAAACTAGGTGAGCTACTTCAAAACAGCCTTATGGGCCAACCCATCTCTGTTGCAAAAGAGTGGGAAGATTTTCAAGTAGAGGTGACACACCTACCGAGCCTAGAGATAGCTGGTTATTCAAGAAACGGATATAGTCCAATTTTAAAACACAATCTTAAGTATACCTAGATTTAAAATTTATTCAAATAAGGTACAGCCTATTTGAAACAGGATACAACCTATAACGAAGGGTAATGTTTTTATGTGACAAAGTAGGCCTAAGAGCAGCCACCTTATGAAAAGCGTTACAGCTTCATCACGACCTCTAATCCCAGTCAAAACCCAAAACCCTTAATATTGAATGACTCCATATTAATATGGAAGACCTTATGTTAGAACTAGTAACAAGAATAATTCTCCACAAGCACGTGTAAGCCGGAATGAATTCACCGGCACTTATCGCCAATGAATAAATGGTAACAACTACAAGAAAACACTACCCATCCAAGCGTTAACCTAACACTAGAACTTTCCAGGAAGATTAAAAAAAGAGGAAGGAACTCGGCAAAAGTAACTCCGC--

</sequence>

<sequence>

<taxon idref="C_alpestris"/>

CTATGCTTAGCCGTAAAATATTAACCTACACAGCCCGCCAGGGAATTACGTCAAGCTTAAAACCCAAAGGACTTGACGGTGCCCCATCCCTCTAGAGGAGCCTGTTCTATAATCGATTCTCCCCGATCTACCTCGCCGTTTCTTGAAATCCAGTCTGTATACCTCCGTCGCAAGCTTACCATGTGAACGTACCAGTGAGCCTAACGATCCCATCCACACGTCAGGTCAAGGTGCAGCCTACGGAACGGAAAGAAATGGGCTACAATCTCTATTAGAACAAACGGAAGACTGTATGAAACACAGTCACTGAAGGCGGATTTAGTAGTAAAAAGAAAATAGAGAGTTCTTTTTAACTGGGCGCTGGGACGCGTACACACCGCCCGTCACCCTCTTCAAAAAG--TCACTAACACCACCCAATTAATAGAAGAGGCAAGTCGTAACATGGTAAGTGTACTGGAAAGTGCACTTGGAACAAAATGTAGCTTAATAAAAGTATCCTGCTTACACCAAGACCATGTCTGTGAAATCCAAACCATTTTGAGCCTAAACCCAAGCCCTACCACATGAATTTTTTCCCCCA-----------TAAAACATTCTTTTGACTAAGTAAAGGCGATTAAAAAGTTTCTAGGAGCCCTAGCACCAGTACCGCAAGGGAAAAGTGAAATAAAAATGAAATAAATTTAAAGCGCTACAAAGTAGAGACCCCTTGTAGTACCTTTTGCATCATGGTCTGACTAGTTTAACCAAGCAAAACGAATTTTAAGTTTGACCCCCCGAAACTAAGCGAGCTACTTCAAGACAGCCTTACGGGCCAACCCATCTCTGTTGCAAAAGAGTGGGAAGATCTTCAAGTAGAGGTGACACACCTATCGAGCCTAGAGATAGCTGGTTATTCAGGAAAAGGATACAGTCCAATTTTAAAACACAACCCTAAGTATATCTAGCTTTAAAATTTATTCAAATAAGGTACAGCCTATTTGAAACAGGATACAACCTACGACGAAGGGTAATGTTTTTATCTGACAAAGTAGGCCTAAGAGCAGCCATCTTATAAAAAGCGTTATAGCTTCATCACCATTACTAATCCTAATCAAAATACAAAACCCTCAATACTGAATGACCCCATACTAATATGGAAGACCTTATGTTAGAACTAGTAACAAGAATAATTCTCCACAAGCACGTGTCAGCCGGAATGAATTCACCGGCATTTACCGCCAATGAATATATGGTAGCAACCACAAGAAAACCCTACCTACCCAAACGTTAACCTCACACTAGAGCTTTCCAGGAAGATTAAAAAAAGGGGAAGGAACTCGGCAAAAGTAACCCCGC--

</sequence>

<sequence>

<taxon idref="C_moranpeep"/>

CTATGCTTAGCCGTAAAATATTAACCCACACAGCCCGCCAGGGAATTACGCCAAGCTTAAAACCCAAAGGACTTGACGGTGTCCCATCCCTCTAGAGGAGCCTGTTCTATAATCGATTCTCCCCGATATACCTCACCGTTTCTTGAAATCCAGTCTGTATACCTCCGTCGCAAGCTTACCATGTGAACGTACCAGTGAGCCTAAAGACCACGCCCACACGTCAGGTCAAGGTGCAGCCCACGGAACGGAAAGAAATGGGCTACAATCTCTACTAGAACAAACGAAAGACTGCATGAAACACAGTCACTGAAGGCGGATTTAGTAGTAAAAAGAAAATAGAGAGTTCTTTTTAACTGGGCGCTGGGACGCGTACACACCGCCCGTCACCCTCTTCAAAAAG--TCACTAACACCCTATAATTAATAGAAGAGGCAAGTCGTAACATGGTAAGTGTACTGGAAAGTGCACTTGGAACAAAATGTAGCTTAATAAAAGTATCTTGCTTACACCAAGACCATGTCTGTGAAACCCAAACCATTTTGAGCCTAAACCTCAGCCCTACTCCATAGATTTCTTTTTTCCCCC--------CAAAACATTCCTCTAACTAAGTAAAGGCGATTAAAAAGTTTCTAGGAGCCTCAGCACCAGTACCGCAAGGGAAAAATGAAATAAAAATGAAATAAATTTAAAGCACTATATA-----------------------------------------AGTTTAACCAAGCAAAACGAATTTTAAGTTTGACCCCCCGAAACTAAGCGAGCTACTTCAAGACAGCCTTATGGGCCAACCCGTCTCTGTTGCAAAAGAGTGGGAAGATTTTTAAGTAGAGGTGACACACCTACCGAGCCTAGAGATAGCTGGTTATTCAGGAAAAGGATATAGTCCAATTTTAAAACACAACCCTAAGTATACCTAGCTTTAAAATTTATTCAAATAAGGTACAGCCTATTTGAAACAGGATACAACCTATAAAGAAGGGTAATGTTTTTACGTGACAGAGTAGGCCTAAGAGCAGCCATCTTTTTAAAAGCGTTATAGCTTTATCACTATTACTAATCCCAACTAAAACCCAAAACCCTCCATACTGAATGACTCCATATTAATATGGAAGACCTTATGTTAGAACTAGTAACAAGAATAATTCTCCACAAGTACGTGTAAGCCGGAATGAATTCACCAGCATTTACCGCCAATGAATAGATGGTAGCAACTACAAGAAAACTCT-CCTACCCAGGCGTTAACCTTACACTAGAGCTTTCCAGGAAGATTAAAAAAAGAGGAAG-------------------------

</sequence>

<sequence>

<taxon idref="C_brevicrus"/>

CCTAGCTTAGCCGTAAAATATTAACCCACACAGCCCGCCAGGGAATTACGTCAAGCTTAAAACCCAAAGGACTTGACGGTGTCCCATCCCTCTAGAGGAGCCTGTTCTATAATCGATTCTCCCCGATATACCTCACCGTTTCTTGAAGTCCAGTCTGTATACCTCCGTCGCAAGCTTACCATGTGAACGTACCAGTGAGCCTAAAGACCCCGTCCACACGTCAGGTCAAGGTGCAGCCCACGAAACGGAAAGAAATGGGCTACAATCTCTACTAGAACAAACGAAAGACTGCATGAAACACAGTCATTGAAGGCGGATTTAGTAGTAAAAAGAAAATAGAGAGTTCTTTTTAACTGGGCGCTGGGACGCGTACACACCGCCCGTCACCCTCTTCAAAAAG--TCACTAACGTCATATAATTTATAGAAGAGGCAAGTCGTAACATGGTAAGTGTACTGGAAAGTGCACTTGGAACAAAATGTAGCTTAATAAAAGCATCCTGCTTACACCAAGACCATGTCTGTGAAACCCAAACCATTTTGAGCCTAAACCTCAGCCCTACTCTGTGAACCTTTTTTCCCCCCC-------CCAAAACATTTTTCTAACTAAGTAAAGGCGATTAAAAAGTTTCTAGGAGCCCTAACACCAGTACCGCRRGGGAAAAGTGAAATAAAAATGAAATAAATTTAAAGCATTATAAAGTAAAGACCCCTTG--GTACCTTTTGCATCATGGTCTGACTAGTTTAACCAAGCAAAACGAATTTTAAGTTTGACCCCCCGAAACTAAGCGAGCTACTTCAAGACAGCCTTATGGGCCAACCCGTCTCTGTCGCAAAAGAGTGGGAAGATTTTCAAGTAGAGGTGACACACCTACCGAGCCTAGAGATAGCTGGTTATTTAGGAAAAGGATACAGTCCAATTTTAAAACACAACCCTAAGTATATTTAGCTTTAAAATTTATTCAAATAAGGTACAGCCTATTTGAAACAGGATACAACCTATGACGAAGGGTAATGTTTTTACGTGACTAAGTAGGCCTAAGAGCAGCCACCTTTTAAAAAGCGTTACAGCTTCATCACCATTACTAATCCCAGCCAAAACCCAAAACCCTCAATACTGAATGACCCCATATTAATATGGAAGACCTTATGTTAGAACTAGTAACAAGAATAATTCTCCACAAGCACGTATAAGCCGGAACGAATTCGCCAGCATTTACCGCCAATGAATAAATGGTAGCAACCACAAGAAAACCCTACCTACCCAAGCGTTAACCTTACACTAGAGCTTTCCAGGAAGATTAAAAAAAGAGGAAGGAACTCGGCAAAAGTAACCCCGCCT

</sequence>

<sequence>

<taxon idref="C_Muller_tiny"/>

CTATGCTTAGCCGTAAAATATTAACCCACACAGCCCGCCAGGGGATTACACCAAGCTTAAAACCCAAAGGACTTGACGGTGTCCCATCCCTCTAGAGGAGCCTGTTCTATAATCGATTCTCCCCGATATACCTCACCGTTTCTTGAAATCCAGCCTGTATACCTCCGTCGCAAGCTTACCATGTGAACGTACCAGTGAGCCTAAAGACCCCGTCCACACGTCAGGTCAAGGTGCAGCCCACGGAACGGAAAGAAATGGGCTACAATCTCTACTAGAACAAACGAAAGACTGCATGAAACACAGTCATTGAAGGCGGATTTAGTAGTAAAAAGAAAATAGAGAGTTCTTTTTAACTGGGCGCTGGGACGCGTACACACCGCCCGTCACCCTCTTCA-AAAAAGTCACTAACATCATATAATTTATAGAAGAGGCAAGTCGTAACATGGTAAGTGTACTGGAAAGTGCACTTGGAACAAAATGTAACTTAATAAAAGTATCCTGCTTACACCAAGACTATGTCTGTGAAACCCAGACCATTTTGAGCCTAAACCTCAGCCCTACTCCATAAACTTTTTTCCCCCCCC-------CAAAAACATTTTTCTGACTAAGTAAAGGCGATTAAAAAGTTTCTAGGAGCCCTAACACCAGTACCGCAAGGGAAAGGTGAAATAAAAATGAAATAAATTTAAAGCATTATAAAGTAAAGACCCCTTGTAGTACCTTTTGCATCATGGTCTGACTAGTTTAACCAAGCAAAACGAATTTTAAGTTTGACCCCCCGAAACTAAGCGAGCTACTTCAAAACAGCCTTATGGGCCAACCCGTCTCTGTCGCAAAAGAGTGGGAAGATTTTCTAGTAGAGGTGACACACCTACCGAGCCTAGAGATAGCTGGTTATTTAGGAAAAGGATATAGTCCAATTTTAAAACACAACCCCAAGTATATTTAGCTTTAAAATTTATTCAAATAAGGTACAGCCTATTTGAAACAGGATACAACCTATGACGAAGGGTAATGTTTTTACGTGACTAAGTAGGCCTAAGAGCAGCCACCTTTTAAAAAGCGTTACAGCTTCATCACCATTACTAATCCCAACCAAAACCCAAAACCCTCGATACTAAATGACCCCATATTAATATGGGAGACCTTATGTTAGAACTAGTAACAAGAATAATTCTCTACAAGCACGTATAAGCCGGAATGAATTCGCCAGCATTTACCGCCAATGAATAAATGGTAACAACTACAAGAAAACCCTACCTACCCAAGCGTTAACCTTACACTAGAGCTTTCCAGGAAGATTAAAAAAAGAGGAAGGAACTCGGCAAAAGTAACCCCGCC-

</sequence>

<sequence>

<taxon idref="C_longirostris"/>

CTATGCTTAGTTGTAAAATATAAACTCACACAAAACGCCAGGGAATTACGCCCTACTTAAAACCCAAAGGACTTGACGGTGTCCCATCCCACTAGAGGAGCCTGTTCTATAATCGATTACCCCCGATTTACCCTACCGCCTCTTGAATCCCAGTCTGTATACCTCCGTCGCAAGCTTACCGTGTGAACGCACAAGTAAGCCAAAAGATATTACCCACACGTCAGGTCAAGGTGCAGCCCACGAAGCGGAAAGAAATGGGCTACAATTTCTATTAGAACAAACGGAAAACTGTATGAAACACAGTTACTGAAGGCGGATTTAGTAGTAAAAAAAAAGCAGAGCGTTCTTTTTAATTAGGCACTGGGACGCGTACACACCGCCCGTCACCCTCTTCAAAAAGATTTCTTAACAACATGCAATAAATAGAAGAGGCAAGTCGTAACATGGTAAGCGTACTGGAAAGTGCACTTGGAATAAAATGTAGCTTAATTAAAGCACCTTGCTTACACCAAGTATATGTCCGTGAAACCCCGACCATTTTAAGCTTAACCCACAGCCCCACCTAAAATGATCTCCAACACAAACAAATTAACTAAAACATTCTATTAACCAAGTAAAGGCGATTAAAAAGTTTATAGGAGCCTCAACAATAGTACCGCAAGGGAAAAATGAAATAAAAATTAAATAAATTTAAAGCACTACAAAGTAGAGATCACTCGTAGTACCTTTTGCATCATGGTCTAACTAGTAAAATCAAGCAAAATGAAACTTAAGTTTGACCCCCCGAAACTAAGCGAGCTACTTCAAAATAGCCTTATGGGCCAACCCATCTCTGTTGCAAAAGAGTGGGGTGATTTTCAAGTAGAGGTGACACACCTACCGAGCCTAGAGATAGCTGGTTATTCAGGAAAAGGATTCAGTCCAATTTTAAATTACATCACTAAATATATTTAGCTTTAAAATTTATTCAAATAAGGTACAGCCTATTTGAAACAGGATACAACCTATTTAGCAGGGTAATGGCCCTGTGCGACCAAGTCGGCCTAATAGCAGCCACCTTATAAAAAGCGTCATAGCTTTATCACAATCATTAATTTGTACCAAAATCCAAAACCCTTTCTACTGAATGACTCCATACCCATATGGAGGACCTAATGTTAGAACTAGTAATAAGAATTATTCTTATAAAGCATGCTTAAGCCGAAATGAACTCACCGGCAGTTACCGCCAATGAATAAACAGTAACAACCACAAGAAAACCCTACTCACCTAAACGTTAACAACACACCAAAGCTTTTTTAGAAGATTAAAAAAGGAGGAAGGAACTCGGCAAAAATAACTTC----

</sequence>

<sequence>

<taxon idref="C_proboscidea_1"/>

CTATGCTTAGCTGTAAAATATAAACTTACACAAAACGCCTGGGAATTACGCCCCACTTAAAACCCAAAGGACTTGACGGTGTCCCATCCAACTAGAGGAGCCTGTTCTATAATCGATTTTCCCCGATTTACCTTACCGCCTCTTGAATCACAGTCTGTATACCTCCGTCGCAAGCTTACCGTGTGAACGCACTAGTAAGCTAAAAGATACTACCAACACGTCAGGTCAAGGTGCAGCCCACGAAGCGGGAAGAAATGGGCTACAATTTCTATTAGAACAAACGAAAAACTGTATGAAACACAGCCACTGAAGGCGGATTTAGTAGTAAAAAAAAAGCAGAGCGTTCTTTTTAACTAGGCACTGGGACGCGTACACACCGCCCGTCACCCTCTTCAAAAAGATTTCTTAACAACACGTAATAAACAGAAGAGGCAAGTCGTAACACGGTAAGCGTACTGGAAAGTGCACTTGGAATAAAATGTAGCTTAATAAAAGCATCTTGCTTACACCAAGCCCATGTCCGTGAAACCCAGACCATTTTAAGCTTAACCCATAGCCCCACCCCGTATAACCTAAAACACAAACAAACCAACCAAAACATTTTAACAACCTAGTAAAGGCGATTAAAAAGTTTATAGGAGCCTCAACAACAGTACCGCAAGGGAAAAGTGAAAT-AAAATTAAAT--AATTAAAGCACCACAAAGTAGAGACCCCTCGTAGTACCTTTTGCATCATGGTCTAACTAGTAAAATCAAGCAAAATGAAATTTAAGTTTGACCCCCCGAAACTAAGCGAGCTACTTCAAAATAGCCTTATGGGCCAACCCATCTCTGTCGCAAAAGAGTGGGATGATTTTCAAGTAGGGGTGACACACCTACCGAGCCTAGAGATAGCTGGTTATTCAGGAAAAGGATCCAGTCCAATTTTAAATTTATACACTAAATATACTTAGCTTTAAAATTTATTCAAATAAGGTACAGCCTATTTGAAACAGGATACAACCTATTTAGCAGGGTAATGGTCCTGTCCGACTAAGTGGGCCTAAAAGCAGCCACCTTATAAAGAGCGTCATAGCTTTATCGCAATCATTAATTTACACCAAAACCCAAAACCCTCACTACTGAATGACTCCATATCCATATGGAAGACCTAATGTTAGAACTAGTAATAAGAACTATTCTTACAAAGCATGCTTAAGCCAGAATGAACTCACTGGCACTCACCGCCAATGAATAAACAGTAGTAACCACAAGAAAACCCTACTTACCCAAACGTTAATAATACACCAAGGCTTTTTTAGAAGATTAAAAAAGGGGGAAGGAACTCGGCAAAAATAACTTC----

</sequence>

<sequence>

<taxon idref="C_gracilirostris"/>

CTATGCCTAGCTGTAAAATATTTACCCACACAAACCACCAGGGAATTACGCCAAGCTTAAAACCCAAAGGACTTGACGGTGTCCCATCCCCCTAGAGGAGCCTGTTCTATAATCGATTATCCCCGATTTACCTCACCACCTCTTGAACCCCAGTCTGTATACCTCCGTCGCAAGCTTACCTTGTGAACGCACCAGTAAGCTAAAAGATATTACCCACACGTCAGGTCAAGGTGCAGCCAACGAGGCGGAAAGAAATGGGCTACAATTTCTACTAGAACAAACGAAAGACTGCATGAAACACAGCCACTGAAGGCGGATTTAGAAGTAAAAAGAAAATAGAGAGTTCTTTTTAAATAGGCACTGGGACGCGTACACACCGCCCGTCACCCTCTTCAAAAAAAGTTATTAACACCACACAATTAACAGAAGAGGCAAGTCGTAACATGGTAAGCGTACTGGAAAGTGCACTTGGAACAAAATATAGCTTAATAAAAGTATCTTGCTTACACCAAGAATATGCCTGTGAAACCCTGACTATTTTGAGCCTGACCCACAGCCCCCTTTGATAAGATTACCCATGTTAATAAATTAATTAAACCATTTTTTTAGCTAAGTAAAGGCGATTAAAAAGCTTCTAGGAGCCCTAACAATAGTACCGCAAGGGAAACTTGAAATAAAACTGAAAC-AATTTAAAGCACTATAAAGTAGAGACCCCTCGTA--ACCTTTTGCATCATGGTCTAACTAGTATACCCAAGCAAAATGAAATTTAAGTTTGACCCCCCGAAACTAGGCGAGCTACTTCAAAATAGCCTCATGGGCCAACCCGTCTCTGTTACAAAAGAGTGGGAAAATTTTCAAGTAGAGGTGACACGCCTACCGAGCCTAGAGATAGCTGGTTATTCAGGAAAAGGATCTAGTCCAATTTTAAACCATATTACTAAATATACTCAGCTTTAAAATTTATTCAAATAAGGTACAGCCTATTTGAAACAGGATACAACCTATTTAGTAGGGTAATGGTTTTATCTGACAAAGTAGGCCTAAAAGCAGCCACCTTTCAAAAAGCGTCACAGCTTAATCATCATCATTGATTTTTAC-AAAATCCAAAACCCTTCTTACTGAATGACTCTATATCTGTATAGAAAACCCTATGTTAGAACTAGTAATAAGAATCATTCTCTCAAAGCCAGTGTAAGCCAAAATGAACTCATCGGCACTTACCGCC-ATGAACAAATAGTAATAACCACAAGAAAACCTTACTTTCCCAAGCGTCAACCTCACGCTAGCGCTTTACAGGAAGATTTAAAAAGGAGGAAGGAACTCGGCAAAAATAACCCC----

</sequence>

<sequence>

<taxon idref="C_burtoni"/>

CTATGCTTAGCTGTAAAATATTAACTTACACAAACCGCCAGGGAATAACGCCAAGCTTAAAACCCAAAGGACTTGACGGTGTCCCACCCTCCTAGAGGAGCCTGTTCTATAATCGATTCTCCCCGATTTACCTCACCACTTCTTGAAACCCAGTCTGTATACCTCCGTCGCAAGCTTACCGTATGAACGCGCTAGTGGGCCAAAAGATACTATCAACACGTCAGGTCAAGGTGCAGCCCACGAAGTGGAAAGAAATGGGCTACAATCTCTATTAGAACAAACGGAAGACTGTATGAAATACAACTACTAAAGGCGGATTTAGCAGTAAAAAGAAACTAGAGAGTTCTTTTTAACTAGGCACTGGAACGCGTACACACCGCCCGTCACCCTCTTCAAAAAG--TTTATAACATTATAC-ATTAATAGAAGAGGCAAGTCGTAACATGGTAAGTGTACTGGAAAGTGCACTTGGAACAAAATGTAGCTTAATTAAAGCATCTTGCTTACACCTAGAACATGCCCGTGAAATCCTGACCATTTTGAGCCTAACCCAAAGCCCTTCCCCCAATAATCTCTCTTATAAACAAACTAACTAAAACATTTTCTTAACCCAGTAAAGGCGATTAAAAAGTTTCTAGGAGCCTTAACTCTAGTACCGCAAGGGAAAAGTGAAATAAAATTGAAAT-AA-TTAAAGCACTGTAAAGTAGAGACCCCTCGTAGTACCTTTTGCATCATGGTTTAACTAGCAAAACCAAGCAAAATGAAATTTAAGTTTGCCCCCCCGAAACTAAGCGAGCTACTTCAAAATAGCCTCACGGGCCAACCCGTCTCTGTTGCAAAAGAGTGGGAAAATTTTTAAGTAGAGGTGATACACCTACCGAGCCTAGAGATAGCTGGTTATTCAGGAAAAGGATCTAGTCCAATTTTAAATCTATACATCAAATACACTTAGTTTTAAAATTTATTCAAATAAGGTACAGCCTATTTGAAACAGGATACAACCTATTAAGCAGGGTAATGCTACTAAATGACCAAGTTGGCCTAAGAGCAGCCACCTTTCAAAAAACGTTACAGCTTCACTATTATCTTTAATATTTACAAAAA-TCAAAACCCTCCTCACTGAATGACTCCATATTTATATGGAAGACCTTATGTTAAAACTAGTAATAAGAATAATTCTCCTAAAGCATGTGTAAGCCGGAATGAATTCACCGGCACTTACCGCCAATGAATAAATAGTAATAACCACAAGAAAACCTTACTTTACTAAACGTTAACCTTACGCTAGAACTTTCCAGGAAGATTAAAAGAGAGGGAAGGAACTCGGCAAAAATAACCCCGCCT

</sequence>

<sequence>

<taxon idref="C_purari"/>

-TATGCCTAGCTGTAAAATATTTACTTACACAAACCGCCAGGGAATTACACCAAGCTTAAAACCCAAAGGACTTGACGGTGTCCCATCCCCCTAGAGGAGCCTGTTCTATAATCGATTCTCCCCGATTTACCCCACCACTTCTTGAAACCCAGTCTGTATACCTCCGTCGCAAGCTTACCGTATGAACGCACTAGTAGGCAAAAAGATTTTATCAACACGTCAGGTCAAGGTGCAGCCTACGAAGTGGAAAGAAATGGGCTACAATCTCTATTAGAATAAACGGAAGGCTGTATGAAACACAGCCAC-TAAGGCGGATTTAGAAGTAAAAAGAAAATAGAGAGTTCTTTTTAACTAGGCCCTGGGACGCGTACACACCGCCCGTCACCCTCTTTAAAAAG--TTAATAACACACTATAATTAATAGAAGAGGCAAGTCGTAACATGGTAAGCGTACTGGAAAGTGCACTTGGAGTAAAATGTAGTTTAACTAAAATTTCTTGCTTACACCAAGAACATGTCTGTGTAACTCAGACCATTTTAAGCCTAACTCACAGCCCTTCTTTATAAA--TTTTACTTCAAACAAACTAACTAAAACATTCTTTCAACTAAGTAAAGGCGATTAAAAAGTTTATAGGAGCCCTAACACTAGTACCGCAAGGGAAAAATGAAAT-AACATGAAAT-AAATTTAAGCACTGTGAAGTA-------------GTACCTTTTGCATCATGGTTTAATTAGTAAAATCAAACAAAATGAAATTTAAGTTTGACCCCCCGAAACTAAGTGAGCTACTTCAAAATAGCCTTAAGGGCCAACCCATCCCTGTTGCAAAAGAGTGGGAAAATTTTCAAGTAGAGGTGATACACCTACCGAACTTAGAGATAGCTGGTTATTCAGGAAAAGGATTTAGTCCAACTTTAAATTATACCACCAGATATCTTTTCTTTTAAAGTTTATTCAAATAAGGTACAGCCTATTTGAAACAGGGTACAACCTATTAAGCAGGGTAATGCTAATACATGACTAAGTTGGCCTAAGAACAGCCACCTTTCAAAAAACGTTACAGCTTCATCATTAATATTTAATTATGTTAAAACCCCAAACCCGAACTACTGAATGACTCCATAATTATATGGAAGACTTTATATTAAAACTAGTAATAAGAATAATTCTCACAAAGTATGTGTAAGCCGGAATGAACTCACCGGCACTTACCGTCAATGAATAATTAGTAATAACCACAAGAAAATCTTACTTTACAAAACGTTAACCTTACACTAGATCCTTACTGGAAGATTAAAAAAAAGGGAAGGAACTCGGCAAAATTAACTCCGCCT

</sequence>

<sequence>

<taxon idref="C_porgera"/>

CTATGCCTAGCTATAAAATATTAACCCACACAAACCGCCAGGGAATTACGCCAAACTTAAAACCCAAAGGACTTGACGGTGTCCCATCCCCCTAGAGGAGCCTGTTCTATAATCGATTCTCCCCGATCTACCTCACCACTTCTTGAACCACAGTCTGTATACCTCCGTCGCAAGCTTACCGCATGAACGTACCAGTAAGCCAAAAGATTTTATCCATACGTCAGGTCAAGGTGCAGCCCACGAAGCGGAAAGAAATGGGCTACAATCTCTACTAGAACAAACGGAAGACTGTATGAAACACAGCTACTAAAGGCGGATTTAGAAGTAAAAAGAAAATAGAGAGTTCTTTTTAACTAGGCACTGGGACGCGTACACACCGCCCGTCACCCTCTTCAAAAAG--TTACTAACACTACATAATTATTAGAAGAGGCAAGTCGTAACATGGTAAGCGTACTGGAAAGTGCACTTGGAACAAAATATAGCTTAATAAAAGTATCTTGCTTACACTAAGAACATGCCAGCAAC-CCCTGGCTATTTTGAGCCTGACCCACAGCCCTTCGCTATAAAATTATCCCCATAA--TGACTAACTAAAACATTCTTTCAACCAAGTAAAGGCGATTAAAAAGTTTTTAGGAGCCCTAACACCAGTACCGCAAGGGAAAAATGAAATAAAAATGAAATAAATTTAAAGCACCATA-AGTAGAGAGCCCTCGTAGTACCTTTTGCATCATGGTCTAACTAGTAAAACCAAGCAAAATGAAATTTAAGTTTGACCCCCCGAAACTAAGCGAGCTACTTAAAAATAGCCTCACGGGCCAACCCATCTCTGTTACAAAAGAGTGGGAAAATTTTTAAGTAGCGGTGACACACCTATCGAGCCTAGAGATAGCTGGTTATTCAAGAAAAGGATCTAGTCCAATTTTAAAACATATCCCCAAATATACTCAGATTTAAAGTTTATTCAAATAAGGTACAGCCTATTTGAAACAGGATACAACCTATTAAGCAGGGTAATGCTACTACGTGACCAAGTCGGCCTAAGAGCAGCCACCTTATAAAAAACGTTACAGCTTCATCATTATCTCTAATATT-ATTAAAACCCAAAACCCTACCTACTGAATGACTCCATATTTATATGGAAGACCTTATGTTAGAACTAGTAATAAGAACACTTCTCATTAAGCACGTGTAAGCCGGAATGAACTCACCGGCATTTGCCGCCAATAAATAAATAGTAATAACCACAAGAAAACCCTACTTTTTCAAGCGTTAACCTTACACTAGACCTTACCTGGAAGATTAAAAAAGAAGGAAGGAACTCGGCAAAAATAACCCCGCCT

</sequence>

<sequence>

<taxon idref="C_grylloides"/>

------TCAGCTGTAAAATATTAACCCACACAAACCACCAGGGAATTACGCCAAGCTTAAAACCCAAAGGACTTGACGGTGTTCCATCCCCCTAGAGGAGCCTGTTCTATAATCGATTTTCCCCGATCTACCTTACCGCCTTTTGAACACCAGTCTGTATACCTCCGTCGTAAGCTTACCGTGTGAACGCACCAGTAAGCCAAAAGATACTACCCACACGTCAGGTCAAGGTGCAGCCTACAAAGCGGGAAGAAATGGGCTACAATCTCTACTAGAACAAACGGAAGACTGCATGAAACACAGCC--TGAAGGCGGATTTAGCAGTAAAAAGAAAACAGAGAGTTCTTTTTAACCGGGCACTGGAACGCGTACACACCGCCCGTCACCCTCTTTAAAAAG--TTACTAACACTACATAATAAACAGAAGAGGCAAGTCGTAACACGGTAAGCGTACTGGAAAGTGCACTTGGAACAAAATGTAGCTTAATTAAAGCATCTTGCTTACACCAAGAACATGTCCG-GAAA-CCTGACCATTTTGAGCCCAGCCCACAGCCCTTCTTAATATAATTCCCCTTATTTACAAATTAACTAAAACATTTTCTTGGCTAAGTAAAGGCGATCAAAAAGCTTCTAGGAGCCCTAACAACAGTACCGCAAGGGAAAAATGAAATAAAAATGAAATAAATTTTAAGCACAATAAAGTAGAGACCCCTCGTAGTACCTTTTGCATCATGGTCTAACTAGCAAAATTAGGCAAAATGAAATTTAAGTCTAACCCCCCGAAACTGGGCGAGCTACTTCAAAACAGCCTCATGGGCTAACCCGTCTCTGTTACAAAAGAGTGGGAAGATTTTCAAGTAGAGGTGATACACCTACCGAGCCCAGAGATAGCTGGTTATTCAGGAAAAGGATCAAGTCCAATTTTAAATC-ACACATCAAATATAACTAGCTTTAAAATTAATTCAAATAAGGTACAGCCTATTTGAAACAGGATACAACCTATATAATAGGGTAATGACCATACACGACCAAGTTGGCCTAAAAGCAGCCACCTTTTAAAAAGCGTCACAGCTTAATCGTTATAGTTAATCTCTATAAAACCCCAAAACCCTCCCTACTGAATGACTCTATATTTATATAGAAAACCTTATGTTAGAACTAGTAATAAGAATTATTCTCTTAAAGCATGTGTGAGCCGGAATGAACTCACCGGCACTTATCGTC-ACGAACAAATAGTAATAACTACAAGAAAACTTTACTTTCCTAAACGTCAATCTCACGCTAGGACTTTTCTGGAAGATTAAAAAAGTAGGAAGGAACTCGGCAAAAATAACCCCGCCT

</sequence>

<sequence>

<taxon idref="C_microps"/>

------------------------------------ACCAGGGAATTACGCCAAGCTTAAAACCCAAAGGACTTGACGGTATCCTATCCCCCTAGAGGAGCCTGTTCTATAATCGATTCCCCCCGATCTACCTCACCACTTCTTGAT-CCCAGTCTATATACCTCCGTCGTAAGCTTACCGTGTGAACGCACTAGTAGGCTAAAAGATTTCACCCATACGTCAGGTCAAGGTGTAGCCTACGAAGCGGAAAGAAATGGGCTACAATCTCTACTAGAACAAACGGAAAGCTGCATGAAACACAGCTTCTGAAGGCGGATTTAGTAGTAAAAAGAAAATAGAGCGTTCTTTTTAACCAGGCACTGGGACGCGTACACACCGCCCGTCACCCTCTTCATAAAG--TTACTAACACCACTTAATCAACAGAAGAGGCAAGTCGTAACATGGTAAGTGTACTGGAAAGTGTACTTGGAACAAAATGTAGCTTAATTAAAGCATCTTGCTTACACCAAGAGTATGTCCGTGAAACTCCAACCATTTTGAGCCTAACTTACAGCCCCCCCCAATAACTCTTTCTTCCCTAACAGACTAATTAAAACATTTAATGAATTTAGTAAAGGTGATTAAAAAACTTCTAGGAG-----------------------------------------------------------------------------------------------------------------------------------------------------------------------------------------------------------------------------------------------------TAGAGATAGCTGGTTATTCAAGAAACGGATATAGTCCAATTTTAAAAAATATCACTAAATACACCCAGCTTTAAAATTTATTCAAATAAGGTACAGCCTATTTGAAACAGGATACAACCTACTCAACAGGGTAATGCTCTAACATGACTAAGTTGGCCTAAAAGCAGCCATCTTTCAAAAAGCGTCATAGCTTAACCATCATCACCAATTATTACTAAAACTCAAAACCCTCCCTATTGAATGAATCTATACCCTTATAGAAAACCTTATGTTAGAACTAGTAATAAGAAAAATTTTCTTAAAGCACGTGTAAGCCAGAATGAACTCCCTGACACTTACCGCC-----ACAAACAGTATTAACTGCAAGAAAAATTTACTTTTCTAATCGTTAACCTCACGCCAGAGCTTTACCGGAAGATTAAAAAAGAGGGAAGGAACTCGGCAAAAATAACTCCGCCT

</sequence>

<sequence>

<taxon idref="C_epirrhinos"/>

------TTAGCTGTAAAATATTAACCTACACAAACCACCCGGGAATTACACCAAGCTTAAAACCCAAAGGACTTGACGGTGTCCTATCCCCCTAGAGGAGCCTGTTCTATTATCGATTCTCCCCGATTTACCTCACCGCCTCTTGAC-TCCAGTCTGTATACCTCCGTCGCAAGCTTACCGTGTGAACGCACCAGTAAGCCAAAAGATCCCACCCACACGTCAGGTCAAGGTGCAGCCCACGAAGCGGAAAGAAATGGGCTACAATTTCTACTAGAACAAACGGAAAACTGTATGAAACACAGTTACTGAAGGCGGATTTAGTAGTAAAAAGAAAGTAGAGTGTTCTTTTTAATCAGGCGCTGGGACGCGTACACACCGCCCGTCACCCTCTTCAAAAAG--TTACTAACACCACTTAATCAACAGAAGAGGCAAGTCGTAACATGGTAAGTGTACTGGAAAGTGCACTTGGAACAAAATGTAGCTTAACAAAAGCATCTTGCTTACACCAAGAACATGCCTGTGAAACCCTGGCCATTTTGAGCCTAACTTATAGCCCAT---CATATATTTTCCCCCACTAACAAATTAACTAAAACATTTTCTTAGTTTAGTAAAGGCGATTAAAAAACTTCTAGGAGCCACAACAACAGTACCGCAAGGGAAAATTGAAATAAAATTGAAATAAATTTAAAGCACTACAAAGTA-------------GTACCTTTTGCATCATGGTCTGACTAGTAAAACCAAGCAAAGTGAAATTTAAGTTTGACTTCCCGAAACTAGACGAGCTACTTCAAAACAGCCTTATGGGCCAACCCCTCCCTGTTGCAAAAGGGTGGGAAGATTTTCAAGTAGAGGTGACACACCTACCGAGCCTAGAGATAGCTGGTTATTCAAGAAAAGGATCTAGTCCAATTTTAAATTACATCACTAAATACACCCAGCTTTAAAATTTATTCAAATAAGGTACAGCCTATTTGAAATAGGATACAACCTATCCAACAGGGTAATGATACCACATGACTAAGTTGGCTTAAAAGCAGCCACCTT-TAAAAAGCGTTACAGCTTAATCACTATCACTAATTACCACTAAAACTCAAAACCCTCCCTATTGAATGAATCTATACCCATATAGAAGACCTTATGTTAGAACTAGTAATAAGAAATTTTCTCTTAAAGCACGTGTAAGCCAGAATGAACTCACTGGCACTTACCGCC-ACAAATGTATAGTATTAACTGCAAGAAAAATCTACTTCTCCAGTCGTTAACCTCACACCAGAGCTTTACCGGAAGATTTAAAAAGGAGGAAGGAACTCGGCAAAAATAACCCCGC--

</sequence>

<sequence>

<taxon idref="C_nigrescens"/>

----GCTCAGCTGTAAAACATTAACCTACACAAACCACCCGGGAATTACGCCAAGCTTAAAACCCAAAGGACTTGACGGTGTCCCATCCCCCTAGAGGAGCCTGTTCTATAATCGATTCTCCCCGATTTACCTCACCGCCTCTTGAC-TCCAGTCTGTATACCTCCGTCGCAAGCTTACCGTGTGAACGCACAAGTGGGCTAAAAGATCTTATCCACACGTCAGGTCAAGGTGCAGCCCACGAAGCGGAAAGAAATGGGCTACAATTTCTACTAGAACAAACGGAAAACTGTATGAAACACAGTTGCTGAAGGCGGATTTAGTAGTAAAAAGAAAGTAGAGCGTTCTTTTTAACCAGACCCTGGGACACGTACACACCGCCCGTCACCCTCTTCAAAAAAAGTTATTAACACCACTTACT-AACAGAAGAGGCAAGTCGTAACATGGTAAGCGTACTGGAAAGTGCACTTGGAACAAAATGTAGCTTAACAAAAGCATCTTGCTTACACCAAGAACATGCCTGTGAAATCCTGGCCATTTTGAGCCTAACTTACAGCCCGC----CTGTATTCTTTTTTCCACAGGAATTAACTAAAACATTTTACTAGTTTAGTAAAGGCGATTAAAAAACTTTCAGGAGCCCTAACAACAGTACCGCAAGGGAAAGATGAAATAAAAATGAAATAAATTTAAAGCACCACAAAGTAGAGACCCCTCG------------CATCATGGTTTAACTAGTAAAACCAAGCAAAATGAGATTTAAGTTTGACTTCCCGAAACTAAGTGAGCTACTTCAAAACAGCCTTACGGGCCAACCCATCCCTGTTGCAAAAGAGTGGGAAGATTTTTAAGTAGAGGTGACACACCTACCGAGCCTAGAGATAGCTGGTTATTCAAGAAAAGGATCTAGTCCAATTTTAAATTATAGTACTAAATACACCCAGCTTTAAAATTTATTCAAATAAGGTACAGCCTATTTGAAACAGGATACAACCTATCCAGCAGGGTAATGATACTACATGTCCAAGTTGGCCTAAAAGCAGCCACCTTTAAAAAAGCGTTATAGCTTAATCACTATTATCAATTTCTACTAAAACCCAAAACCCTCCCTACTGAATGAATCTATACCAATATAGAAGACCTTATGTTAAAACTAGTAATAAGAAATTTTCTCCTAAAGCACGTGTAAGCCAGAATGAACTCACCGGCACTTACCGCCAA-GAATCTACAGTATTAACTGCAAGAAAAATCTACTTCTCAAATCGTTAACCTCACGCCAGAGCTTTACCGGAAGATTTAAAAAGAGGGAAGGAACTCGGC----------------

</sequence>

<sequence>

<taxon idref="C_sp_foyas"/>

--------------------TTAACCTACACAAACCACCCGGGAATTACGCCAAGCTTAAAACCCAAAGGACTTGACGGTGTCCTATCCCCCTAGAGGAGCCTGTTCTATAATCGATACCCCCCGATCTACCTCACCGCTTCTTGAC-ATCAGTCTGTATACCTCCGTCGCAAGCTTACCGTGTGAACGCACCAGTAAGCTAAAAGATTCCACCTACACGTCAGGTCAAGGTGCAACCTACGAAACGGAAAGAAATGGGCTACAATCTCTACTAGAACAAACGGAAAACTGTATGAAATACAGTTACTGAAGGCGGATTTAGTAGTAAAAAGAAAATAGAGTGTTCTTTTTAACCAGGCACTGGGACGCGTACACACCGCCCGTCACCCTCTTCAAAAAG--TTATTAACACCCCCCAATCAACAGAAGAGGCAAGTCGTAACATGGTAAGTGTACTGGAAAGTGCGCTTGGAACAAAATGTAGCTTAACTAAAGCATCTTGCTTACACCAAGAATATGCCTGCGAAATCCTGACCATTTTGAGCCTAACCTACAGCCCC-CCCCATATATTTTTCCCCCCCAAAAGACTAACTAAAACATTCTCTTGGTTAAGTAAAGGCGATTAAAAACCCACTAGGAGCCCCAACAACAGTACCGCAAGGGAATTATGAAATAAAAATGAAATAAATTTAAAGCACTCTTAAAGT-------------GTACCTTTTGCATCATGGTCTAACTAGTAAAACCAAGCAAAGTGAAACTTAAGTTTGACTTCCCGAAACTAGACGAGCTACTTTAAAACAGCCTTATGGGCCAACCCATCCCTGTTGCAAAAGAGTGGGAAGATTTTCAAGTAGAGGTGACACGCCTACCGAGCCTAGAGATAGCTGGTTATTCAAGAAAAGGATATAGTCCAATTTTAAGCCAAATCACTAAATACACCCAGCTTTAAAATTTATTCAAATAAGGTACAGCCTATTTGAAACAGGATACAACCTATTCAGCAGGGTAATGTTCTCACATGATTAAGTCGGCCTAAAAGCAGCCACCTTTCAAAAAGCGTCACAGCTTAATCATCATCACTAATTACTATTAAAACTCAAAACCCTCTCTACTGAATGAATCTATACCCATATAGAAAACCTTATGTTAGAACTAGTAATGAGAAAATTTCTCCTAAAGCATGTATAAGCCAGAATGAACTCCCTGGCACTTACCGCC-----ATAAACAGTATTAACCACAAGAAAAATTTACTTTTCTAATCGTTAACCTTACACTAGAGCTTTACCGGAAGATTAAAAAAGGGGGAAGGAACTCGGCAAAAATAACTCCGCCT

</sequence>

<sequence>

<taxon idref="C_rostellifer"/>

CTATGCTTAGCTGTAAAATATTAACCTACACCAACCACCCGGGAATTACGCCAAGCTTAAAACCCAAAGGACTTGACGGTGTCCTATCCCCCTAGAGGAGCCTGTTCTATAATCGATTCCCCCCGATCTACCTCACCACTTCTTGACACCCAGCCTGTATACCTCCGTCGCAAGCTTACCGTGTGAGCGCACCAGTGAGCTAAAAGATATCACCCATACGTCAGGTCAAGGTGCAGCCTACGAAGCGGAAAGAAATGGGCTACAATCTCTACTAGAACAAACGGAAAACTGTGTAAAACACAGTTACTGAAGGCGGATTTAGTAGTAAAAAGAAAATAGAGTGTTCTTTTTAACCAGGCACTGGGACGCGTACACACCGCCCGTCACCCTCTTCAAAAAG--TTACTAACACCACTTAATCAACAGAAGAGGCAAGTCGTAACATGGTAAGCGTACTGGAAAGTGCACTTGGAACAAAATGTAGCTTAACTAAAGCATCTTGCTTACACCAAGAATATGCCTGCAAAACCCTGACCATTTTGAGCTTAACTTACAGCCCCCCCCCATAATCTTCTTTTCCCATAAAAACTAACTAAAACATTTTCTTAGTCAAGTAAAGGCGATCAAAAAACTTCTAGGAGCCCCAACAACAGTACCGCAAGGGAATTATGAAATAAAAATGAAATAAATTTAAAGCACTATAAAGTAGAGACCCCTCGTA--------------------------------------------------------------------------------------------------------------------------------------------------------------------------------------------------------------------------------------------------------------------------------------------------------------------------------------------------------------------------------------------------------------------------------------------------------------------------------------------------------------------------------------------------------------------------------------------------------------------------------------------------

</sequence>

<sequence>

<taxon idref="C_amomani"/>

------------------------------------ACCAGGGAATTACGCCAAGCTTAAAACCCAAAGGACTTGACGGTGTCCCATCCCCCTAGAGGAGCCTGTTCTATAATCGATTTCCCCCGATCTACCTCACCGCTTCTCGAACCCCAGTCTGTATACCTCCGTCGCAAGCTTACCGTGTGAGCGCACTAGTAAGCTAAAAGATTCCACCTACACGTCAGGTCAAGGTGCAGCCTATGGAGCGGAAAGAAATGGGCTACAATCTCTACTAGAACAAACGGAAAGCTGTATGAAACACAGCTACTGAAGGCGGATTTAGTAGTAAAAAGAAAACAGAGTGTTCTTTTTAACCAGGCACTGGGACGCGTACACACCGCCCGTCACCCTCTTCAAAAAG--TTATTAACACCACTTAATCAACAGAAGAGGCAAGTCGTAACATGGTAAGTGTACTGGAAAGTGCACTTGGAACAAAATGTAGCTTAACTAAAGCATCTTGCTTACACCAAGAATATGCCTGCGAAATCCTAGCCATCTTGAGCCTAATCTACAGCCCCTCCCACAATGATTTCCCCCCCAAATAAACTAACTAAAACATTCTCTTGGTTAAGTAAAGGCGATTAAAAAACCTCTAGGAG-----------------------------------------------------------------------------------------------------------------------------------------------------------------------------------------------------------------------------------------------------TAGAGATAGCTGGTTATTCAGGAAAAGGATATAGTCCAATTTTAAGCCCCCACACTAAATGCACC-CGCTTTAAAATTTATTCAAATAAGGTACAGCCTATTTGAAACAGGATACAACCTATTCAACAGGGTAATGTTCTTACATAACTAAGTTGGCCTAAAAGCAGCCACCTTCCAAAAAGCGTCATAGCTTAATTATAATCACCAATTTATATAAAAACCCAAAACCCTCTCTACTGAATGAATCTATACCCATATAGAAAACCTTATGTTAGAACTAGTAATAAGAAAATTTCTCGCAAAGCATGTATAAGCCAGAATGAACTCCCTGGCATTTACCGCT-----ATTAACAGTATTAACCACAAGAAAAGTTTACTTTTCTAATCGTTAACCTTACACTAGAGCTTTACCGGAAGATTAAAAAAGGGGGAAGGAACTCGGCAAAAATAACTCCGCCT

</sequence>

<sequence>

<taxon idref="C_arndatorum"/>

-------------------------------------------AATTACGCCAAGCTTAAAACCCAAAGGACTTGACGGTGTCCCATCCCCCTAGAGGAGCCTGTTCTATAATCGATTCCCCCCGATCCACCCCACCGCTTCTTGACACCCAGTCTGTATACCTCCGTCGCAAGCTTACCGTGTGAACGCACTAGTAAGCTAAAAGATTCCACCCACACGTCAGGTCAAGGTGCAGCCTACGAAGCGGAAAGAAATGGGCTACAATCTCTACTAGAACAAACGAAAAACTGTATGAAACACAGTTACTGAAGGCGGATTTAGTAGTAAAAAGAAAGCAGAGCGTTCTTTTTAACCAGGCACTGGGACGCGTACACACCGCCCGTCACCCTCTTCAAAAAAAGTTACTAACACTACTTAATCAACAGAAGAGGCAAGTCGTAACACGGTAAGTGTACTGGAAAGTGCACTTGGAACAAAATGTAGCTTAACTAAAGCATCTTGCTTACACCAAGAATATGCCTGAGAAACCCTGACCATTTTGAGCCTGACCCACAGCCCCACTCTATATAAACCCCCCCATTAATAAACTGACTAAAGCATTCTCTCAGTTAAGTAAAGGCGATTAAAAAACTTCTAGGAG--------------------------------------------------------------------------------------------------------------------------------------------------------------------------------------------------------------------------------------------------------------------------------------GTCCAATTTTAAACCCCAACACTAAATATAACCAGCTTTAAAATTTATTCAAATAAGGTACAGCCTATTTGAAACAGGATACAACCTATTCAGCAGGGTAATGTTCTCACATGACTAAGTTGGCCTAAAAGCAGCCACCTTTCAAAAAGCGTCATAGCTTAATTATCATCACCAATTAATATTAAAACTCAAAACCCTCTCTACTGAATGAATCTATACCCATATAGAAAACCTTATGTTAGAACTAGTAATAAGAAGACTTCTCTTAAAGCACGTATAAGCCAGAATGAACTCCCTGGCACTTACCGCC-----ATAAACAGTATTAACCACAAGAAAAGTTTACTTTTTTTATCGTTAACCTTACGCCAGAGCTTTACCGGAAGATTAAAAAGGGGGGAAGGAACTCGGCAAAAATAACTCCGCCT

</sequence>

<sequence>

<taxon idref="C_siegfriedi"/>

-------------------------------------------------------------------------------------------------------------------------------------------------------------------------------------------------------------------------------------------------------------------------------------------------------------------------------------------------------------------------------------------------------------------------------------------------------------------------------------------------------------------------------------------------------------------------------------------------------------------------------------------------------------------------------------------------------------------------------------------------GTACCTTTTGCATCATGGTCTAACTAGTTTAACCAAGCAAAACGAATTTTAAGTTTGCCCCCCCGAAACTAAGCGAGCTACTTCAAAACAGCCTTATGGGCCAACCCATCTCTGTTGCAAAAGAGTGGGAAGATTTTCAAGTAGAGGTGACACACCTACCGAGCCTAGAGATAGCTGGTTATTCAAGAAAAGGATACAGTCCAATTTTAAAATACAACCTGAAATATACCTAGTTTTAAAATTTATTCAAATAAGGTACAGCCTATTTGAAACAGGATACAACCTATTACGTAGGGTAATGTTTTCATATGACAAAGTAGGCCTAAGAGCAGCCACCTTATAAAAAGCGTTACAGCTTCACCATCATCACCAATCCCAACTAAAACTCAAAACCCTCAATATTGAATGACCCCATACCTATATGGAAAACCCTATGTTAGAACTAGTAACAAGAATAATTCTCCTAAAGCACGTGTAAGCCAGAATGAATCCGCTGGCATTTACCGCCAATGAATAAACAGTAGCAACCACAAGAAAATCCTACTCACCCAGACGTTAACCTCACACCAGAGCTTTCCCGGAAGATTAAAAAAAGGGGAAGGAACTCGGCAAAA------------

</sequence>

</alignment>

<!-- Altitude 1 (each sequence refers to a taxon above). -->

<alignment id="alignment3_alt_1">

<dataType idref="alt_1"/>

<sequence>

<taxon idref="Copiula_obsti"/>

?

</sequence>

<sequence>

<taxon idref="Copiula_pipiens"/>

?

</sequence>

<sequence>

<taxon idref="Cophixalus_balbus"/>

?

</sequence>

<sequence>

<taxon idref="Oreophryne_atrigularis"/>

?

</sequence>

<sequence>

<taxon idref="Liophryne_dentata"/>

?

</sequence>

<sequence>

<taxon idref="Oreophryne_sibilans"/>

?

</sequence>

<sequence>

<taxon idref="Liophryne_schlaginhaufeni"/>

?

</sequence>

<sequence>

<taxon idref="Oreophryne_unicolor"/>

?

</sequence>

<sequence>

<taxon idref="Genyophryne_thomsoni"/>

?

</sequence>

<sequence>

<taxon idref="Cophixalus_humicola"/>

?

</sequence>

<sequence>

<taxon idref="Oreophryne_clamata"/>

?

</sequence>

<sequence>

<taxon idref="A_exclamitans"/>

L

</sequence>

<sequence>

<taxon idref="C_sp_nov1"/>

H

</sequence>

<sequence>

<taxon idref="Cophixalus_tridactylus"/>

?

</sequence>

<sequence>

<taxon idref="Copiula_major"/>

?

</sequence>

<sequence>

<taxon idref="Oxydactyla_crassa"/>

?

</sequence>

<sequence>

<taxon idref="C_foyas_2"/>

L

</sequence>

<sequence>

<taxon idref="C_laurini"/>

L

</sequence>

<sequence>

<taxon idref="C_tuberculus"/>

H

</sequence>

<sequence>

<taxon idref="C_sp4"/>

L

</sequence>

<sequence>

<taxon idref="C_sp_juha"/>

?

</sequence>

<sequence>

<taxon idref="C_sp3"/>

L

</sequence>

<sequence>

<taxon idref="C_darlingtoni"/>

M

</sequence>

<sequence>

<taxon idref="C_pandanicolus"/>

M

</sequence>

<sequence>

<taxon idref="C_fafniri"/>

M

</sequence>

<sequence>

<taxon idref="C_foyas_1"/>

L

</sequence>

<sequence>

<taxon idref="C_alpestris"/>

U

</sequence>

<sequence>

<taxon idref="C_moranpeep"/>

L

</sequence>

<sequence>

<taxon idref="C_brevicrus"/>

M

</sequence>

<sequence>

<taxon idref="C_Muller_tiny"/>

M

</sequence>

<sequence>

<taxon idref="C_longirostris"/>

L

</sequence>

<sequence>

<taxon idref="C_proboscidea_1"/>

H

</sequence>

<sequence>

<taxon idref="C_gracilirostris"/>

H

</sequence>

<sequence>

<taxon idref="C_burtoni"/>

L

</sequence>

<sequence>

<taxon idref="C_purari"/>

H

</sequence>

<sequence>

<taxon idref="C_porgera"/>

M

</sequence>

<sequence>

<taxon idref="C_grylloides"/>

H

</sequence>

<sequence>

<taxon idref="C_microps"/>

H

</sequence>

<sequence>

<taxon idref="C_epirrhinos"/>

H

</sequence>

<sequence>

<taxon idref="C_nigrescens"/>

H

</sequence>

<sequence>

<taxon idref="C_sp_foyas"/>

L

</sequence>

<sequence>

<taxon idref="C_rostellifer"/>

H

</sequence>

<sequence>

<taxon idref="C_amomani"/>

L

</sequence>

<sequence>

<taxon idref="C_arndatorum"/>

H

</sequence>

<sequence>

<taxon idref="C_siegfriedi"/>

M

</sequence>

</alignment>

<!-- ecology (each sequence refers to a taxon above). -->

<alignment id="alignment4_ecology">

<dataType idref="ecology"/>

<sequence>

<taxon idref="Copiula_obsti"/>

T

</sequence>

<sequence>

<taxon idref="Copiula_pipiens"/>

T

</sequence>

<sequence>

<taxon idref="Cophixalus_balbus"/>

S

</sequence>

<sequence>

<taxon idref="Oreophryne_atrigularis"/>

S

</sequence>

<sequence>

<taxon idref="Liophryne_dentata"/>

T

</sequence>

<sequence>

<taxon idref="Oreophryne_sibilans"/>

S

</sequence>

<sequence>

<taxon idref="Liophryne_schlaginhaufeni"/>

T

</sequence>

<sequence>

<taxon idref="Oreophryne_unicolor"/>

S

</sequence>

<sequence>

<taxon idref="Genyophryne_thomsoni"/>

T

</sequence>

<sequence>

<taxon idref="Cophixalus_humicola"/>

T

</sequence>

<sequence>

<taxon idref="Oreophryne_clamata"/>

S

</sequence>

<sequence>

<taxon idref="A_exclamitans"/>

S

</sequence>

<sequence>

<taxon idref="C_sp_nov1"/>

S

</sequence>

<sequence>

<taxon idref="Cophixalus_tridactylus"/>

T

</sequence>

<sequence>

<taxon idref="Copiula_major"/>

T

</sequence>

<sequence>

<taxon idref="Oxydactyla_crassa"/>

T

</sequence>

<sequence>

<taxon idref="C_foyas_2"/>

S

</sequence>

<sequence>

<taxon idref="C_laurini"/>

S

</sequence>

<sequence>

<taxon idref="C_tuberculus"/>

S

</sequence>

<sequence>

<taxon idref="C_sp4"/>

S

</sequence>

<sequence>

<taxon idref="C_sp_juha"/>

S

</sequence>

<sequence>

<taxon idref="C_sp3"/>

S

</sequence>

<sequence>

<taxon idref="C_darlingtoni"/>

S

</sequence>

<sequence>

<taxon idref="C_pandanicolus"/>

S

</sequence>

<sequence>

<taxon idref="C_fafniri"/>

S

</sequence>

<sequence>

<taxon idref="C_foyas_1"/>

S

</sequence>

<sequence>

<taxon idref="C_alpestris"/>

T

</sequence>

<sequence>

<taxon idref="C_moranpeep"/>

S

</sequence>

<sequence>

<taxon idref="C_brevicrus"/>

T

</sequence>

<sequence>

<taxon idref="C_Muller_tiny"/>

T

</sequence>

<sequence>

<taxon idref="C_longirostris"/>

S

</sequence>

<sequence>

<taxon idref="C_proboscidea_1"/>

S

</sequence>

<sequence>

<taxon idref="C_gracilirostris"/>

T

</sequence>

<sequence>

<taxon idref="C_burtoni"/>

S

</sequence>

<sequence>

<taxon idref="C_purari"/>

T

</sequence>

<sequence>

<taxon idref="C_porgera"/>

T

</sequence>

<sequence>

<taxon idref="C_grylloides"/>

T

</sequence>

<sequence>

<taxon idref="C_microps"/>

T

</sequence>

<sequence>

<taxon idref="C_epirrhinos"/>

T

</sequence>

<sequence>

<taxon idref="C_nigrescens"/>

S

</sequence>

<sequence>

<taxon idref="C_sp_foyas"/>

T

</sequence>

<sequence>

<taxon idref="C_rostellifer"/>

S

</sequence>

<sequence>

<taxon idref="C_amomani"/>

T

</sequence>

<sequence>

<taxon idref="C_arndatorum"/>

T

</sequence>

<sequence>

<taxon idref="C_siegfriedi"/>

S

</sequence>

</alignment>

<!-- The unique patterns from 1 to end -->

<!-- npatterns=755 -->

<patterns id="molec.patterns" from="1" strip="false">

<alignment idref="alignment1"/>

</patterns>

<!-- The unique patterns from 1 to end -->

<!-- npatterns=1 -->

<patterns id="alt_1.patterns" from="1" strip="false">

<alignment idref="alignment3_alt_1"/>

</patterns>

<!-- The unique patterns from 1 to end -->

<!-- npatterns=1 -->

<patterns id="ecology.patterns" from="1" strip="false">

<alignment idref="alignment4_ecology"/>

</patterns>

<!-- A prior on the distribution node heights defined given -->

<!-- Using birth-death model on tree: Gernhard T (2008) J Theor Biol, Volume 253, Issue 4, Pages 769-778 In press-->

<birthDeathModel id="birthDeath" units="substitutions">

<birthMinusDeathRate>

<parameter id="birthDeath.meanGrowthRate" value="270.0" lower="0.0"/>

</birthMinusDeathRate>

<relativeDeathRate>

<parameter id="birthDeath.relativeDeathRate" value="0.5" lower="0.0"/>

</relativeDeathRate>

</birthDeathModel>

<!-- This is a simple constant population size coalescent model -->

<!-- that is used to generate an initial tree for the chain. -->

<constantSize id="initialDemo" units="substitutions">

<populationSize>

<parameter id="initialDemo.popSize" value="100.0"/>

</populationSize>

</constantSize>

<!-- Generate a random starting tree under the coalescent process -->

<coalescentSimulator id="startingTree">

<coalescentSimulator>

<taxa idref="Ingroup"/>

<constantSize idref="initialDemo"/>

</coalescentSimulator>

<taxa idref="taxa"/>

<constantSize idref="initialDemo"/>

</coalescentSimulator>

<!-- Generate a tree model -->

<treeModel id="treeModel">

<coalescentTree idref="startingTree"/>

<rootHeight>

<parameter id="treeModel.rootHeight"/>

</rootHeight>

<nodeHeights internalNodes="true">

<parameter id="treeModel.internalNodeHeights"/>

</nodeHeights>

<nodeHeights internalNodes="true" rootNode="true">

<parameter id="treeModel.allInternalNodeHeights"/>

</nodeHeights>

</treeModel>

<!-- Taxon Sets -->

<tmrcaStatistic id="tmrca(Albericus)" includeStem="false">

<mrca>

<taxa idref="Albericus"/>

</mrca>

<treeModel idref="treeModel"/>

</tmrcaStatistic>

<tmrcaStatistic id="tmrca(Choerophryne)" includeStem="false">

<mrca>

<taxa idref="Choerophryne"/>

</mrca>

<treeModel idref="treeModel"/>

</tmrcaStatistic>

<tmrcaStatistic id="tmrca(Ingroup)" includeStem="false">

<mrca>

<taxa idref="Ingroup"/>

</mrca>

<treeModel idref="treeModel"/>

</tmrcaStatistic>

<monophylyStatistic id="monophyly(Ingroup)">

<mrca>

<taxa idref="Ingroup"/>

</mrca>

<treeModel idref="treeModel"/>

</monophylyStatistic>

<!-- Generate a speciation likelihood for Yule or Birth Death -->

<speciationLikelihood id="speciation">

<model>

<birthDeathModel idref="birthDeath"/>

</model>

<speciesTree>

<treeModel idref="treeModel"/>

</speciesTree>

</speciationLikelihood>

<!-- The uncorrelated relaxed clock (Drummond, Ho, Phillips & Rambaut (2006) PLoS Biology 4, e88 ) for molecular data-->

<discretizedBranchRates id="molec.branchRates">

<treeModel idref="treeModel"/>

<distribution>

<logNormalDistributionModel meanInRealSpace="true">

<!-- set the mean to some sensible value, e.g. 1% per lineage per million years-->

<mean>

<parameter id="ucld.mean" value="0.01"/>

</mean>

<stdev>

<parameter id="ucld.stdev" value="0.3333333333333333" lower="0.0"/>

</stdev>

</logNormalDistributionModel>

</distribution>

<rateCategories>

<parameter id="branchRates.categories"/>

</rateCategories>

</discretizedBranchRates>

<rateStatistic id="meanRate" name="meanRate" mode="mean" internal="true" external="true">

<treeModel idref="treeModel"/>

<discretizedBranchRates idref="molec.branchRates"/>

</rateStatistic>

<rateStatistic id="coefficientOfVariation" name="coefficientOfVariation" mode="coefficientOfVariation" internal="true" external="true">

<treeModel idref="treeModel"/>

<discretizedBranchRates idref="molec.branchRates"/>

</rateStatistic>

<rateCovarianceStatistic id="covariance" name="covariance">

<treeModel idref="treeModel"/>

<discretizedBranchRates idref="molec.branchRates"/>

</rateCovarianceStatistic>

<!-- the strict clock for alt_1 (Uniform rates across branches) -->

<strictClockBranchRates id="alt_1.branchRates">

<rate>

<parameter id="alt_1.clock.rate" value="0.01" lower="0.0" upper="1.0"/>

</rate>

</strictClockBranchRates>

<!-- the strict clock for ecology (Uniform rates across branches) -->

<strictClockBranchRates id="ecology.branchRates">

<rate>

<parameter id="ecology.clock.rate" value="0.01" lower="0.0" upper="1.0"/>

</rate>

</strictClockBranchRates>

<!-- The general time reversible (GTR) substitution model and 1 partition - selected by Partitionfinder BIC -->

<gtrModel id="gtr">

<frequencies>

<frequencyModel dataType="nucleotide">

<frequencies>

<parameter id="frequencies" value="0.25 0.25 0.25 0.25"/>

</frequencies>

</frequencyModel>

</frequencies>

<rateAC>

<parameter id="ac" value="1.0" lower="0.0"/>

</rateAC>

<rateAG>

<parameter id="ag" value="1.0" lower="0.0"/>

</rateAG>

<rateAT>

<parameter id="at" value="1.0" lower="0.0"/>

</rateAT>

<rateCG>

<parameter id="cg" value="1.0" lower="0.0"/>

</rateCG>

<rateGT>

<parameter id="gt" value="1.0" lower="0.0"/>

</rateGT>

</gtrModel>

<!-- site model -->

<siteModel id="molec.siteModel">

<substitutionModel>

<gtrModel idref="gtr"/>

</substitutionModel>

<gammaShape gammaCategories="4">

<parameter id="alpha" value="0.5" lower="0.0"/>

</gammaShape>

<proportionInvariant>

<parameter id="pInv" value="0.5" lower="0.0" upper="1.0"/>

</proportionInvariant>

</siteModel>

<frequencyModel id="alt_1.frequencyModel">

<kStateType idref="alt_1"/>

<frequencies>

<parameter id="alt_1.frequencies" dimension="4" value="0.250 0.250 0.250 0.250"/>

</frequencies>

</frequencyModel>

<generalSubstitutionModel id="alt_1.ord" >

<kStateType idref="alt_1"/>

<frequencies>

<frequencyModel idref="alt_1.frequencyModel"/>

</frequencies>

<rates>

<parameter id="ForeAndHind.RateMatrix" value="1.0 0.0 0.0 1.0 0.0 1.0" dimension="6" lower="0" upper="Infinity" />

<!--

As there is no operator on the starting values in this rate matrix, this enforces an ordered character.

The 3 elements above correspond to the 3 entries in the matrix, in left-right order per row.

U M L H

U - 1 0 0

M - 1 0

L - 1

H -

-->

</rates>

</generalSubstitutionModel>

<siteModel id="alt_1.siteModel">

<substitutionModel>

<generalSubstitutionModel idref="alt_1.ord"/>

</substitutionModel>

</siteModel>

<!-- ecology: The general Substitution Model for binary data -->

<!-- Note: Lewis model assumes equal rates thus same equilibrium frequencies, hence no operator-->

<frequencyModel id="ecology.frequencyModel">

<dataType idref="ecology"/>

<frequencies>

<parameter id="ecology.freqs" value="0.5 0.5"/>

</frequencies>

</frequencyModel>

<lewisMk totalOrder="false" id="ecology.unord">

<frequencies>

<frequencyModel idref="ecology.frequencyModel"/>

</frequencies>

</lewisMk>

<siteModel id="ecology.siteModel">

<substitutionModel>

<generalSubstitutionModel idref="ecology.unord"/>

</substitutionModel>

</siteModel>

<!-- START Multivariate diffusion model -->

<multivariateDiffusionModel id="size.diffusionModel">

<precisionMatrix>

<matrixParameter id="size.precision">

<parameter id="col1" value="0.05"/>

</matrixParameter>

</precisionMatrix>

</multivariateDiffusionModel>

<multivariateWishartPrior id="size.precisionPrior" df="1">

<scaleMatrix>

<matrixParameter>

<parameter value="1.0"/>

</matrixParameter>

</scaleMatrix>

<data>

<parameter idref="size.precision"/>

</data>

</multivariateWishartPrior>

<!-- END Multivariate diffusion model -->

<!-- Likelihood for tree given sequence data -->

<treeLikelihood id="molec.treeLikelihood" useAmbiguities="false">

<patterns idref="molec.patterns"/>

<treeModel idref="treeModel"/>

<siteModel idref="molec.siteModel"/>

<discretizedBranchRates idref="molec.branchRates"/>

</treeLikelihood>

<ancestraltreeLikelihood id="alt_1.treeLikelihood" useAmbiguities="false" stateTagName="alt_1">

<patterns idref="alt_1.patterns"/>

<treeModel idref="treeModel"/>

<siteModel idref="alt_1.siteModel"/>

<strictClockBranchRates idref="alt_1.branchRates"/>

<lewisMk idref="alt_1.ord"/>

</ancestraltreeLikelihood>

<ancestraltreeLikelihood id="ecology.treeLikelihood" useAmbiguities="false" stateTagName="ecology">

<patterns idref="ecology.patterns"/>

<treeModel idref="treeModel"/>

<siteModel idref="ecology.siteModel"/>

<strictClockBranchRates idref="ecology.branchRates"/>

<lewisMk idref="ecology.unord"/>

</ancestraltreeLikelihood>

<!-- START Multivariate diffusion model -->

<multivariateTraitLikelihood id="size.traitLikelihood" traitName="size" useTreeLength="true" scaleByTime="true" reportAsMultivariate="true" reciprocalRates="true" integrateInternalTraits="true">

<multivariateDiffusionModel idref="size.diffusionModel"/>

<treeModel idref="treeModel"/>

<traitParameter>

<parameter id="leaf.size"/>

</traitParameter>

<conjugateRootPrior>

<meanParameter>

<parameter value="0.0"/>

</meanParameter>

<priorSampleSize>

<parameter value="0.001"/>

</priorSampleSize>

</conjugateRootPrior>

<!-- Jitter points with identical values -->

<jitter window="0.001 0.001" duplicatesOnly="true">

<parameter idref="leaf.size"/>

</jitter>

</multivariateTraitLikelihood>

<!-- END Multivariate diffusion model -->

<!-- Define operators -->

<operators id="operators" optimizationSchedule="default">

<scaleOperator scaleFactor="0.75" weight="0.1">

<parameter idref="ac"/>

</scaleOperator>

<scaleOperator scaleFactor="0.75" weight="0.1">

<parameter idref="ag"/>

</scaleOperator>

<scaleOperator scaleFactor="0.75" weight="0.1">

<parameter idref="at"/>

</scaleOperator>

<scaleOperator scaleFactor="0.75" weight="0.1">

<parameter idref="cg"/>

</scaleOperator>

<scaleOperator scaleFactor="0.75" weight="0.1">

<parameter idref="gt"/>

</scaleOperator>

<deltaExchange delta="0.01" weight="0.1">

<parameter idref="frequencies"/>

</deltaExchange>

<scaleOperator scaleFactor="0.75" weight="0.1">

<parameter idref="alpha"/>

</scaleOperator>

<scaleOperator scaleFactor="0.75" weight="0.1">

<parameter idref="pInv"/>

</scaleOperator>

<scaleOperator scaleFactor="0.75" weight="3">

<parameter idref="ucld.stdev"/>

</scaleOperator>

<scaleOperator scaleFactor="0.75" weight="10">

<parameter idref="alt_1.clock.rate"/>

</scaleOperator>

<scaleOperator scaleFactor="0.75" weight="10">

<parameter idref="ecology.clock.rate"/>

</scaleOperator>

<subtreeSlide size="0.1" gaussian="true" weight="15">

<treeModel idref="treeModel"/>

</subtreeSlide>

<narrowExchange weight="15">

<treeModel idref="treeModel"/>

</narrowExchange>

<wideExchange weight="5">

<treeModel idref="treeModel"/>

</wideExchange>

<wilsonBalding weight="5">

<treeModel idref="treeModel"/>

</wilsonBalding>

<FixedNodeheightSubtreePruneRegraft weight="5">

<treeModel idref="treeModel"/>

</FixedNodeheightSubtreePruneRegraft>

<NearestNeighborInterchange weight="5">

<treeModel idref="treeModel"/>

</NearestNeighborInterchange>

<scaleOperator scaleFactor="0.75" weight="3">

<parameter idref="treeModel.rootHeight"/>

</scaleOperator>

<uniformOperator weight="30">

<parameter idref="treeModel.internalNodeHeights"/>

</uniformOperator>

<scaleOperator scaleFactor="0.75" weight="3">

<parameter idref="birthDeath.meanGrowthRate"/>

</scaleOperator>

<scaleOperator scaleFactor="0.75" weight="3">

<parameter idref="birthDeath.relativeDeathRate"/>

</scaleOperator>

<upDownOperator scaleFactor="0.75" weight="3">

<up>

<!--rate fixed <parameter idref="ucld.mean"/> -->

</up>

<down>

<parameter idref="treeModel.allInternalNodeHeights"/>

</down>

</upDownOperator>

<swapOperator size="1" weight="10" autoOptimize="false">

<parameter idref="branchRates.categories"/>

</swapOperator>

<uniformIntegerOperator weight="10">

<parameter idref="branchRates.categories"/>

</uniformIntegerOperator>

<upDownOperator scaleFactor="0.75" weight="3">

<up>

<parameter idref="alt_1.clock.rate"/>

</up>

<down>

<parameter idref="treeModel.allInternalNodeHeights"/>

</down>

</upDownOperator>

<upDownOperator scaleFactor="0.75" weight="3">

<up>

<parameter idref="ecology.clock.rate"/>

</up>

<down>

<parameter idref="treeModel.allInternalNodeHeights"/>

</down>

</upDownOperator>

<!-- START Multivariate diffusion model -->

<precisionGibbsOperator weight="1">

<multivariateTraitLikelihood idref="size.traitLikelihood"/>

<multivariateWishartPrior idref="size.precisionPrior"/>

</precisionGibbsOperator>

<!-- END Multivariate diffusion model -->

</operators>

<!-- Define MCMC -->

<mcmc id="mcmc" chainLength="100000000" autoOptimize="true" operatorAnalysis="Exemplar_ConcMuscGB_new.ops">

<posterior id="posterior">

<prior id="prior">

<booleanLikelihood>

<monophylyStatistic idref="monophyly(Ingroup)"/>

</booleanLikelihood>

<gammaPrior shape="0.05" scale="10.0" offset="0.0">

<parameter idref="ac"/>

</gammaPrior>

<gammaPrior shape="0.05" scale="20.0" offset="0.0">

<parameter idref="ag"/>

</gammaPrior>

<gammaPrior shape="0.05" scale="10.0" offset="0.0">

<parameter idref="at"/>

</gammaPrior>

<gammaPrior shape="0.05" scale="10.0" offset="0.0">

<parameter idref="cg"/>

</gammaPrior>

<gammaPrior shape="0.05" scale="10.0" offset="0.0">

<parameter idref="gt"/>

</gammaPrior>

<uniformPrior lower="0.0" upper="1.0">

<parameter idref="frequencies"/>

</uniformPrior>

<exponentialPrior mean="0.5" offset="0.0">

<parameter idref="alpha"/>

</exponentialPrior>

<uniformPrior lower="0.0" upper="1.0">

<parameter idref="pInv"/>

</uniformPrior>

<exponentialPrior mean="0.3333333333333333" offset="0.0">

<parameter idref="ucld.stdev"/>

</exponentialPrior>

<uniformPrior lower="0.0" upper="100000.0">

<parameter idref="birthDeath.meanGrowthRate"/>

</uniformPrior>

<uniformPrior lower="0.0" upper="1.0">

<parameter idref="birthDeath.relativeDeathRate"/>

</uniformPrior>

<speciationLikelihood idref="speciation"/>

<!-- START Multivariate diffusion model -->

<multivariateWishartPrior idref="size.precisionPrior"/>

<!-- END Multivariate diffusion model -->

</prior>

<likelihood id="likelihood">

<treeLikelihood idref="molec.treeLikelihood"/>

<treeLikelihood idref="alt_1.treeLikelihood"/>

<treeLikelihood idref="ecology.treeLikelihood"/>

<!-- START Multivariate diffusion model -->

<multivariateTraitLikelihood idref="size.traitLikelihood"/>

<!-- END Multivariate diffusion model -->

</likelihood>

</posterior>

<operators idref="operators"/>

<!-- write log to screen -->

<log id="screenLog" logEvery="100000">

<column label="Posterior" dp="4" width="12">

<posterior idref="posterior"/>

</column>

<column label="Prior" dp="4" width="12">

<prior idref="prior"/>

</column>

<column label="Likelihood" dp="4" width="12">

<likelihood idref="likelihood"/>

</column>

<column label="rootHeight" sf="6" width="12">

<parameter idref="treeModel.rootHeight"/>

</column>

<column label="alt_1.rate" sf="6" width="18">

<parameter idref="alt_1.clock.rate"/>

</column>

<column label="ecology.rate" sf="6" width="18">

<parameter idref="ecology.clock.rate"/>

</column>

</log>

write log to file -->

<log id="fileLog" logEvery="100000" fileName="Choero_run_2_new.log" overwrite="false">

<posterior idref="posterior"/>

<prior idref="prior"/>

<likelihood idref="likelihood"/> <!-- total likelihood of molec and phenotypic data -->

<parameter idref="treeModel.rootHeight"/>

<tmrcaStatistic idref="tmrca(Albericus)"/>

<tmrcaStatistic idref="tmrca(Choerophryne)"/>

<tmrcaStatistic idref="tmrca(Ingroup)"/>

<parameter idref="birthDeath.meanGrowthRate"/>

<parameter idref="birthDeath.relativeDeathRate"/>

<parameter idref="ac"/>

<parameter idref="ag"/>

<parameter idref="at"/>

<parameter idref="cg"/>

<parameter idref="gt"/>

<parameter idref="frequencies"/>

<parameter idref="alpha"/>

<parameter idref="pInv"/>

<parameter idref="ucld.mean"/> <!-- fixed -->

<parameter idref="ucld.stdev"/>

<rateStatistic idref="meanRate"/>

<rateStatistic idref="coefficientOfVariation"/>

<rateCovarianceStatistic idref="covariance"/>

<parameter idref="alt_1.clock.rate"/>

<parameter idref="ecology.clock.rate"/>

<!-- START Multivariate diffusion model -->

<matrixParameter idref="size.precision"/>

<!-- END Multivariate diffusion model -->

<!-- START Multivariate diffusion model -->

<multivariateTraitLikelihood idref="size.traitLikelihood"/>

<!-- END Multivariate diffusion model -->

<treeLikelihood idref="molec.treeLikelihood"/>

<treeLikelihood idref="alt_1.treeLikelihood"/>

<treeLikelihood idref="ecology.treeLikelihood"/>

<speciationLikelihood idref="speciation"/>

</log>

<!-- write tree log to file -->

<logTree id="treeFileLog" logEvery="100000" nexusFormat="true" fileName="Choero_run_2_time.trees" sortTranslationTable="true">

<treeModel idref="treeModel"/>

<ancestralTreeLikelihood idref="alt_1.treeLikelihood"/>

<ancestralTreeLikelihood idref="ecology.treeLikelihood"/>

<multivariateTraitLikelihood idref="size.traitLikelihood"/>

<trait name="rate" tag="rate">

<discretizedBranchRates idref="molec.branchRates"/>

</trait>

<posterior idref="posterior"/>

</logTree>

<logTree id="substTreeFileLog" logEvery="100000" nexusFormat="true" fileName="Choero_run_2_sub.trees" branchLengths="substitutions">

<treeModel idref="treeModel"/>

<discretizedBranchRates idref="molec.branchRates"/>

<trait name="rate" tag="rate">

<discretizedBranchRates idref="molec.branchRates"/>

</trait>

</logTree>

</mcmc>

<!-- START Marginal Likelihood Estimator -->

<!-- Define marginal likelihood estimator settings -->

<marginalLikelihoodEstimator chainLength="1000" pathSteps="100" pathScheme="betaquantile" alpha="0.3">

<samplers>

<mcmc idref="mcmc"/>

</samplers>

<pathLikelihood id="pathLikelihood">

<source>

<posterior idref="posterior"/>

</source>

<destination>

<prior idref="prior"/>

</destination>

</pathLikelihood>

<log id="MLELog" logEvery="1000" fileName="Choero_run_2.log">

<pathLikelihood idref="pathLikelihood"/>

</log>

</marginalLikelihoodEstimator>

<!-- Path sampling estimator from collected samples -->

<pathSamplingAnalysis fileName="Exemplar_ConcMuscGB_new.mle.log">

<likelihoodColumn name="pathLikelihood.delta"/>

<thetaColumn name="pathLikelihood.theta"/>

</pathSamplingAnalysis>

<!-- Stepping-stone sampling estimator from collected samples -->

<steppingStoneSamplingAnalysis fileName="Exemplar_ConcMuscGB_new.mle.log">

<likelihoodColumn name="pathLikelihood.delta"/>

<thetaColumn name="pathLikelihood.theta"/>

</steppingStoneSamplingAnalysis>

<!-- END Marginal Likelihood Estimator -->

<report>

<property name="timer">

<mcmc idref="mcmc"/>

</property>

</report>

</beast>
